# Association of Racial and Socioeconomic Disparities With Outcomes Among Patients Hospitalized With Acute Myocardial Infarction, Heart Failure, and Pneumonia: An Analysis of Within- and Between-Hospital Variation

**DOI:** 10.1001/jamanetworkopen.2018.2044

**Published:** 2018-09-07

**Authors:** Nicholas S. Downing, Changqin Wang, Aakriti Gupta, Yongfei Wang, Sudhakar V. Nuti, Joseph S. Ross, Susannah M. Bernheim, Zhenqiu Lin, Sharon-Lise T. Normand, Harlan M. Krumholz

**Affiliations:** 1 Brigham and Women’s Hospital, Boston, Massachusetts; 2 Center for Outcomes Research and Evaluation, Yale-New Haven Hospital, New Haven, Connecticut; 3 Department of Internal Medicine, Yale School of Medicine, New Haven, Connecticut; 4 Section of Cardiovascular Medicine, Department of Internal Medicine, Yale School of Medicine, New Haven, Connecticut; 5 Yale School of Medicine, New Haven, Connecticut; 6 Section of General Internal Medicine, Yale School of Medicine, New Haven, Connecticut; 7 National Clinician Scholars Program, Yale School of Medicine, New Haven, Connecticut; 8 Department of Health Policy and Management, Yale School of Public Health, New Haven, Connecticut; 9 Department of Health Care Policy, Harvard Medical School, Boston, Massachusetts; 10 Department of Biostatistics, Harvard T.H. Chan School of Public Health, Boston, Massachusetts; zoi18011411 Dr Changqin Wang is now with Pratt & Whitney, East Hartford, Connecticut.; zoi18011412 Dr Gupta is now with Division of Cardiology, Department of Medicine, Columbia University, New York, New York.

## Abstract

**Importance:**

Although studies have described differences in hospital outcomes by patient race and socioeconomic status, it is not clear whether such disparities are driven by hospitals themselves or by broader systemic effects.

**Objective:**

To determine patterns of racial and socioeconomic disparities in outcomes within and between hospitals for patients with acute myocardial infarction, heart failure, and pneumonia.

**Design, Setting, and Participants:**

Retrospective cohort study initiated before February 2013, with additional analyses conducted during the peer-review process. Hospitals in the United States treating at least 25 Medicare fee-for-service beneficiaries aged 65 years or older in each race (ie, black and white) and neighborhood income level (ie, higher income and lower income) for acute myocardial infarction, heart failure, and pneumonia between 2009 and 2011 were included.

**Main Outcomes and Measures:**

For within-hospital analyses, risk-standardized mortality rates and risk-standardized readmission rates for race and neighborhood income subgroups were calculated at each hospital. The corresponding ratios using intraclass correlation coefficients were then compared. For between-hospital analyses, risk-standardized rates were assessed according to hospitals’ proportion of patients in each subgroup. These analyses were performed for each of the 12 analysis cohorts reflecting the unique combinations of outcomes (mortality and readmission), demographics (race and neighborhood income), and conditions (acute myocardial infarction, heart failure, and pneumonia).

**Results:**

Between 74% (3545 of 4810) and 91% (4136 of 4554) of US hospitals lacked sufficient racial and socioeconomic diversity to be included in this analysis, with the number of hospitals eligible for analysis varying among cohorts. The 12 analysis cohorts ranged in size from 418 to 1265 hospitals and from 144 417 to 703 324 patients. Within included hospitals, risk-standardized mortality rates tended to be lower among black patients (mean [SD] difference between risk-standardized mortality rates in black patients compared with white patients for acute myocardial infarction, −0.57 [1.1] [*P* = .47]; for heart failure, −4.7 [1.3] [*P* < .001]; and for pneumonia, −1.0 [2.0] [*P* = .05]). However, risk-standardized readmission rates among black patients were higher (mean [SD] difference between risk-standardized readmission rates in black patients compared with white patients for acute myocardial infarction, 4.3 [1.4] [*P *< .001]; for heart failure, 2.8 [1.8] [*P* < .001], and for pneumonia, 3.7 [1.3] [*P* < .001]). Intraclass correlation coefficients ranged from 0.68 to 0.79, indicating that hospitals generally delivered consistent quality to patients of differing races. While the coefficients in the neighborhood income analysis were slightly lower (0.46-0.60), indicating some heterogeneity in within-hospital performance, differences in mortality rates and readmission rates between the 2 neighborhood income groups were small. There were no strong, consistent associations between risk-standardized outcomes for white or higher-income neighborhood patients and hospitals’ proportion of black or lower-income neighborhood patients.

**Conclusions and Relevance:**

Hospital performance according to race and socioeconomic status was generally consistent within and between hospitals, even as there were overall differences in outcomes by race and neighborhood income. This finding indicates that disparities are likely to be systemic, rather than localized to particular hospitals.

## Key Points

**Question:**

What is the source of known disparities in outcomes after hospitalization according to patients’ race and socioeconomic status?

**Findings:**

This cohort study found differences in outcomes by race and neighborhood income, but hospital performance within and between hospitals by patient race and neighborhood income was generally consistent.

**Meaning:**

There was no evidence that specific hospitals that treated patients from a range of racial and socioeconomic backgrounds contributed to observed disparities after hospitalization, suggesting a systemic effect may be contributory.

## Teaser

This cohort study uses Medicare claims data to compare performance within and between US hospitals concerning the association of race and neighborhood income with patient outcomes after hospitalization for acute myocardial infarction (AMI), heart failure, and pneumonia.

## Introduction

Although achieving health equity is a long-standing goal for the American health care system, disparities in health outcomes persist.^1,2^ Hospitals can play a key role in influencing patients’ outcomes, and encouraging trends in hospital performance have been documented^3,4,5^; however, it is important to know whether hospital performance differs according to patients’ race and socioeconomic status. A key question is whether disparities in outcomes vary within hospitals, and if so, whether their magnitude is consistent across all hospitals, suggesting a systemic effect, or varies, with some hospitals having larger differences in performance by race and income than others. A closely related question is whether disparities are driven by the concentration of patients of different races and socioeconomic status in high- or low-performing hospitals. To determine whether disparities were concentrated in specific institutions or represented a more systemic problem, we used patient-level Medicare claims data to investigate differences in outcomes in acute myocardial infarction (AMI), heart failure (HF), and pneumonia within and between hospitals.

First, we determined whether there were within-hospital differences in performance between patient groups defined by race and socioeconomic status. We used risk-standardized outcome measures that control for age, sex, and clinical covariates to characterize within-hospital differences in performance by comparing the performance that each hospital achieved among its black patients with that among its white patients, and among patients from lower-income neighborhoods vs those from higher-income neighborhoods. Specifically, we used 30-day risk-standardized mortality rates (RSMRs) and risk-standardized readmission rates (RSRRs) to determine absolute differences in outcomes between race and neighborhood income subgroups and the corresponding risk-standardized ratios, which describe hospitals’ expected performance relative to their predicted performance reflecting patient mix, to determine whether performance was similar across patient subgroups treated at the same hospital relative to how other hospitals treated those groups.

Second, we sought to determine whether black patients and patients from lower-income neighborhoods were disproportionately treated at lower-performing hospitals. To do this, we described between-hospital differences in quality by assessing whether the outcomes of white patients varied by the proportion of black patients at a given hospital. Similarly, we determined whether the quality of care provided to patients from higher-income neighborhoods varied by the proportion of patients from lower-income neighborhoods seen at the hospital. The simultaneous evaluation for the presence of within-hospital and between-hospital differences can help clarify the source of these disparities, thereby guiding efforts to eliminate disparities associated with these common diagnoses.

## Methods

### Sample Construction

Using the Medicare Standard Analytic Files, claims involving Medicare fee-for-service beneficiaries aged 65 years or older discharged from a nonfederal acute care hospital between January 1, 2009, and December 31, 2011, with a principal diagnosis of AMI, HF, or pneumonia were identified following methods adopted by the Centers for Medicare & Medicaid Services in the calculation of publicly reported quality measures for these conditions.^6,7,8,9,10,11^ Next, the Medicare Enrollment Database was used to identify patients who died during the study period, and the Medicare Standard Analytic Files were used to determine whether patients were readmitted to an acute care hospital within 30 days of their discharge for any cause. Patients’ race was determined using the Medicare Standard Analytic Files; information about race was self-reported by patients to the Centers for Medicare & Medicaid Services. To allow the classification of claims according to neighborhood income level, the median income for the zip code associated with each claim was determined using data from the 2011 American Community Survey.^12^ This study followed the Strengthening the Reporting of Observational Studies in Epidemiology (STROBE) reporting guideline. The Yale University Human Investigation Committee reviewed the study and waived the requirement for informed consent.

### Analysis Cohorts

To measure differences in mortality and readmission among race and neighborhood income subgroups for each of the 3 conditions, we constructed 12 analysis cohorts, again following the approach used by the Centers for Medicare & Medicaid Services in its computation of risk-standardized rates (eFigure 1 in the Supplement): the mortality analyses excluded patients with a length of stay of 1 day or less, those transferred from another hospital, and those enrolled in a hospice program; the readmission analyses excluded patients transferred to another acute care facility, those who died during hospitalization or within 30 days of discharge, those who were discharged against medical advice, and those who had been readmitted from an index admission that occurred in the prior 30 days. The analyses according to race focused on black patients and white patients; all others were excluded. Claims were then aggregated to the hospital level. Hospitals discharging fewer than 25 white patients and 25 black patients for each condition over the study period were excluded to reflect the minimum volume of patients needed to reliably compute risk-standardized measures. For analyses according to neighborhood income, approximately 2% of patients were excluded because the median income of their zip code could not be ascertained. Patients were assigned to the higher-income neighborhood subgroup if the median neighborhood income associated with their zip code fell in the highest tertile of neighborhood income for their condition, and into the lower-income neighborhood subgroup if it fell into the lowest tertile for their condition. Patients with a median neighborhood income falling between the 2 thresholds were excluded. The number of discharges for each of the 3 conditions at each hospital were counted, and subsequently hospitals that discharged fewer than 25 patients from each of the 2 neighborhood income groups for each condition were excluded.

### Risk-Standardized Mortality and Readmission Measures

Thirty-day mortality was defined as death due to any cause in the 30 days following the date of the initial hospitalization. Thirty-day readmission was defined as the occurrence of a subsequent hospital admission for any cause in the 30 days following the date of discharge from the initial hospitalization. Thirty-day RSMRs and RSRRs and the corresponding standardized ratios were estimated for the 2 race subgroups and the 2 neighborhood income subgroups in each of the analysis cohorts by fitting a series of established hierarchical generalized linear models.^6,7,8,9,10,11^ This method simultaneously uses 2 levels (patient and hospital) to partition the variance in outcomes within and between hospitals. At the first level, the log-odds of mortality or of readmission were modeled for a patient treated in a particular hospital as a function of age (as a continuous variable), sex, clinical covariates that were associated with the outcome of interest during the initial development of these models, and a hospital-specific intercept. At the second level, the hospital-specific intercepts were modeled as an overall mean and a random error term, assumed to be normal with a mean of 0 and unknown variance. Hospital-specific intercepts are included to accommodate the clustering of outcomes for patients treated at the same hospital.^13^ Using these models, 30-day risk-standardized mortality and readmission ratios, defined as the ratio of predicted to expected outcomes, were calculated separately for race and neighborhood income subgroups for all hospitals in each of the 12 analysis cohorts. The predicted outcome describes the number of outcomes predicted by the model at each hospital that reflects each hospital’s patient mix and its individual hospital-specific effect. In contrast, the expected outcome describes the number of outcomes expected by the model at each hospital that reflects each hospital’s patient mix but uses an average for all hospital-specific effects. In addition, the corresponding RSMRs and RSRRs were computed by multiplying the ratios of predicted to expected outcomes by the observed rate of each outcome in the appropriate subgroup.

### Statistical Analysis

Descriptive statistics were used to characterize the hospitals that make up the 12 analysis cohorts using the 2010 American Hospital Association Annual Survey of Hospitals.^14^ In addition, several patient characteristics, including age, sex, race, neighborhood income level, and comorbid conditions, were summarized at the hospital level for each of the 12 analysis cohorts, stratifying by race and neighborhood income subgroup as appropriate.

To understand within-hospital differences in outcomes, risk-standardized mortality rates and ratios and risk-standardized readmission rates and ratios were computed for each hospital’s black patients and separately for each hospital’s white patients, and the differences between the 2 groups were calculated; *t* tests weighted according to the proportion of patients in each group were performed to assess the magnitude of the differences. A similar approach to characterize within-hospital differences between patients from lower-income neighborhoods and higher-income neighborhoods was used. The measurement of risk-standardized ratios is important because these measures compare the predicted number of outcomes at each hospital with the expected number, without considering the absolute rates in each group, which could have been influenced by subgroups of hospitals. Intraclass correlation coefficients (ICCs) were estimated as an additional measure of consistency of hospital performance for patients in different race subgroups: a low absolute value of the ICC would suggest that hospitals’ ratios of predicted to expected outcomes among black patients were not associated with the corresponding ratios among white patients or, in other words, that hospitals’ proportion of predicted outcomes over expected outcomes varied by racial groups. We used a similar approach to characterize within-hospital differences in quality between patients from lower-income neighborhoods and higher-income neighborhoods.

To describe between-hospital differences according to race, the risk-standardized rates were calculated in each cohort for black patients, for white patients, and for all patients combined. Next, these rates were plotted stratifying hospitals by the decile of admissions of black patients and using the Kruskal-Wallis test to determine whether there were differences in the rates among deciles; additionally, Pearson correlation coefficients were calculated to assess associations between risk-standardized rates and the proportion of black patients or patients from lower-income neighborhoods treated as a continuous variable. A similar approach was used to describe between-hospital differences according to neighborhood income.

Last, we repeated all analyses using more permissive inclusion criteria that allowed hospitals treating at least 10 patients in both race and neighborhood income subgroups to be included in these respective sensitivity analyses. Analyses were conducted with SAS statistical software version 9.3 (SAS Institute Inc) and initiated before February 2013. Additional analyses were conducted during the peer-review process.

## Results

### Hospital and Patient Characteristics

A significant proportion of hospitals in the United States (between 74% [3545 of 4810] and 91% [4136 of 4554], depending on the cohort) lacked sufficient diversity in their patient population (ie, treatment of ≥25 patients in each race or income subgroup during the 3-year study period) to be included in our analysis (Table 1). The size of the resulting analysis cohorts varied from 418 hospitals in the analysis of AMI mortality according to race to 1265 hospitals in the analysis of HF readmission according to neighborhood income. The hospitals included in our analysis tended to be large and were more frequently teaching hospitals than those that were excluded (eTables 1-4 in the Supplement). In the analyses by race, a high proportion of hospitals from the South region were included in the sample, while the regional distribution of hospitals included in the analysis of neighborhood income was not different from the excluded hospitals. Mortality rates tended to be slightly lower and readmission rates slightly higher in included hospitals across all analyses.

**Table 1.** Sample Construction

Outcome and Analysis Cohort	No./Total No. (%)	
	Hospitals	Admissions
**Race**		
Mortality		
Acute myocardial infarction	418/4554 (9.2)	144 417/493 164 (29.3)
Heart failure	1077/4762 (22.6)	507 799/1 013 864 (50.1)
Pneumonia	840/4793 (17.5)	335 659/1 000 573 (33.5)
Readmission		
Acute myocardial infarction	424/4451 (9.5)	174 719/505 508 (34.6)
Heart failure	1257/4772 (26.3)	703 324/1 254 861 (56.0)
Pneumonia	907/4803 (18.9)	378 496/1 068 546 (35.4)
**Neighborhood Income**		
Mortality		
Acute myocardial infarction	741/4587 (16.2)	161 142/506 436 (31.8)
Heart failure	1147/4797 (23.9)	371 102/1 040 886 (35.7)
Pneumonia	1160/4831 (24.0)	308 079/1 029 366 (29.9)
Readmission		
Acute myocardial infarction	764/4484 (17.0)	192 435/518 696 (37.1)
Heart failure	1265/4810 (26.3)	494 811/1 288 840 (38.4)
Pneumonia	1227/4842 (25.3)	342 284/1 099 264 (31.2)

Hospitals included in our analysis accounted for a disproportionate number of admissions: for example, 18.9% of hospitals (907 of 4803) were included in the analysis of pneumonia readmission by race, yet these hospitals accounted for 35.4% of admissions (378 496 of 1 068 546) in the study period. The number of hospital admissions ranged from 144 417 in the analysis of AMI mortality by race to 703 324 in the analysis of HF readmissions by race.

Among included admissions in the race analyses, the median proportion of black patients at the hospital level among these cohorts ranged from 15.9% to 19.0%, with white patients making up the remainder. A smaller proportion of black patients were male (40.8%-43.9%) than of white patients (46.1%-53.4%) (eTables 5 and 6 in the Supplement). Black patients also tended to be younger (mean age of each analysis cohort, 77.0-78.4 years) than white patients (mean age of each analysis cohort, 78.2-81.3 years). The proportion of patients from the higher-income neighborhoods and lower-income neighborhoods was approximately equal in all the income cohorts, with the median proportion of patients from lower-income neighborhoods ranging from 47.9% to 53.9%. A smaller proportion of patients from lower-income neighborhoods were male (45.4%-51.8%) than of patients from higher-income neighborhoods (46.5%-53.4%) (eTables 7 and 8 in the Supplement). Patients from lower-income neighborhoods also tended to be slightly younger (mean age of each analysis cohort, 77.6-79.8 years) than those from higher-income neighborhoods (mean age of each analysis cohort, 78.8-81.8 years).

### Within-Hospital Performance

#### Race

For all 3 conditions, black patients had lower 30-day RSMRs than white patients treated at the same hospital (Table 2). The mean (SD) within-hospital difference in 30-day RSMR between black patients and white patients was −0.57% (1.1%) (*P* = .47) for AMI, −4.7% (1.3%) (*P* < .001) for HF, and −1.0% (2.0%) (*P* = .05) for pneumonia. In contrast, RSRRs for black patients were higher than those of white patients for all 3 conditions: mean (SD) within-hospital difference in 30-day rates between black patients and white patients was 4.3% (1.4%) (*P* < .001) for AMI, 2.8% (1.8%) (*P* < .001) for HF, and 3.7% (1.3%) (*P* < .001) for pneumonia.

**Table 2.** Risk-Standardized Ratios and Rates Characterizing Within-Hospital Differences in Mortality and Readmission.

Outcome and Analysis Cohort	Risk-Standardized Rates		Risk-Standardized Ratios		Intraclass Correlation Coefficient (95% CI)
	Within-Hospital Difference, Mean (SD), %^a^	*P* Value^b^	Within-Hospital Difference, Mean (SD)^a^	*P* Value^a^	
**Race**					
Mortality					
Acute myocardial infarction	−0.57 (1.1)	.47	−9.1 × 10^−4^ (0.076)	.91	0.68 (0.64-0.72)
Heart failure	−4.7 (1.3)	<.001	−2.1 × 10^−3^ (0.12)	.89	0.72 (0.69-0.75)
Pneumonia	−1.0 (2.0)	.05	−2.4 × 10^−3^ (0.16)	.50	0.70 (0.67-0.73)
Readmission					
Acute myocardial infarction	4.3 (1.4)	<.001	6.3 × 10^−4^ (0.069)	.83	0.73 (0.69-0.77)
Heart failure	2.8 (1.8)	<.001	6.2 × 10^−4^ (0.073)	.74	0.73 (0.71-0.75)
Pneumonia	3.7 (1.3)	<.001	1.3 × 10^−3^ (0.068)	.87	0.79 (0.76-0.82)
**Neighborhood Income**					
Mortality					
Acute myocardial infarction	0.06 (0.87)	.83	−6.7 × 10^−4^ (0.062)	.91	0.46 (0.42-0.50)
Heart failure	−1.1 (1.3)	.04	3.3 × 10^−3^ (0.12)	.75	0.59 (0.56-0.62)
Pneumonia	0.15 (1.5)	.41	−1.8 × 10^−3^ (0.13)	.66	0.57 (0.54-0.60)
Readmission					
Acute myocardial infarction	0.74 (1.1)	.41	−3.2 × 10^−4^ (0.060)	.97	0.60 (0.57-0.63)
Heart failure	1.1 (1.8)	.26	2.0 × 10^−4^ (0.075)	.96	0.60 (0.57-0.63)
Pneumonia	1.2 (1.4)	.10	2.4 × 10^−4^ (0.073)	.92	0.57 (0.54-0.60)

^a^ In race analyses, differences are reported as the rate or ratio among black patients less the rate or ratio among white patients. In neighborhood income analyses, differences are reported as the rate or ratio among patients from lower-income neighborhoods less the rate or ratio among patients from higher-income neighborhoods.

^b^ *P* values calculated using *t* tests weighted by the proportion of patients in each subgroup treated at each hospital.

**Figure 1.**
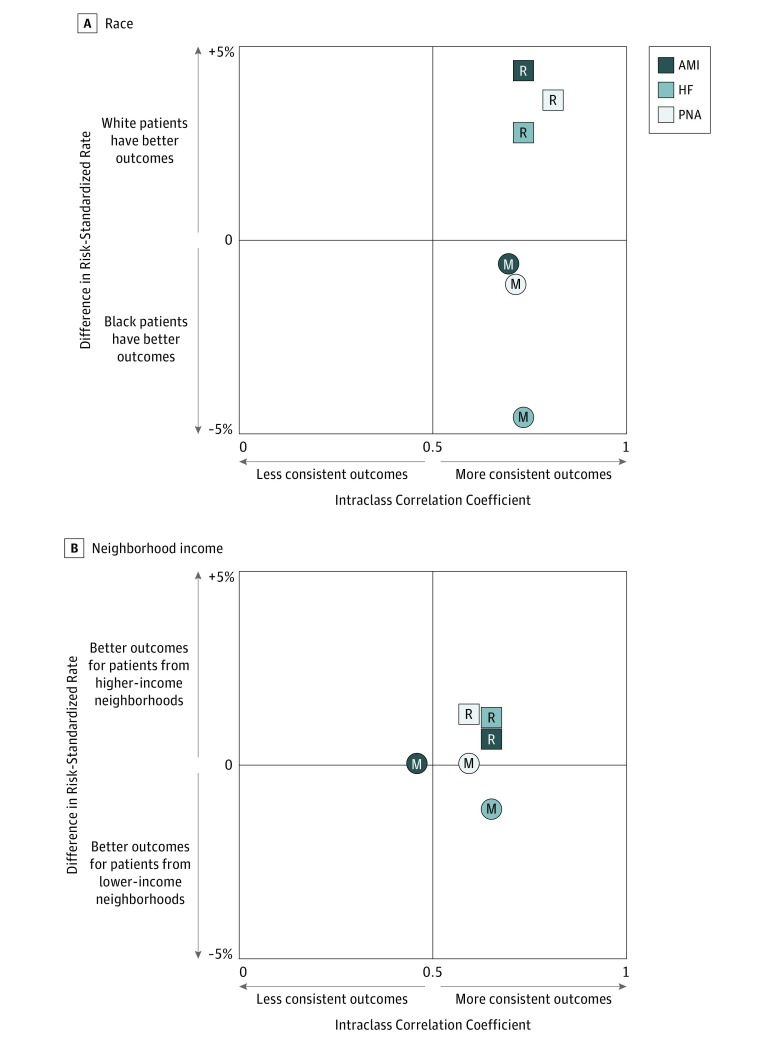
Consistency of Hospital Quality and Differences in Rates of Risk-Standardized Outcomes The matrix depicts mortality analyses as circles and readmission analyses as squares. Comparisons are shown for race (A) and neighborhood income (B). AMI indicates acute myocardial infarction; HF, heart failure; M, mortality; PNA, pneumonia; and R, readmission.

The mean within-hospital differences in risk-standardized mortality and readmission ratios for black patients and for white patients were small (Table 2). The ICC of the mortality ratios by race was 0.68 (95% CI, 0.64-0.72) for AMI, 0.72 (95% CI, 0.69-0.75) for HF, and 0.70 (95% CI, 0.67-0.73) for pneumonia, indicating that those hospitals that produced low risk-standardized mortality ratios among their white patients also had low risk-standardized mortality ratios for their black patients, even though the risk-standardized rates differed (Figure 1A). Hospital performance was particularly consistent with respect to readmissions: the ICC was 0.73 (95% CI, 0.69-0.77) for AMI, 0.73 (95% CI, 0.71-0.75) for HF, and 0.79 (95% CI, 0.76-0.82) for pneumonia.

#### Neighborhood Income

Although there were small differences in RSMRs and RSRRs between neighborhood income groups, with patients from lower-income neighborhoods appearing to have slightly worse outcomes, the differences generally were not significant. The mean (SD) within-hospital differences in RSMRs between patients from lower-income neighborhoods and those from higher-income neighborhoods were 0.06% (0.87%) (*P* = .83) for AMI, −1.1% (1.3%) (*P* = .04) for HF, and 0.15% (1.5%) (*P* = .41) for pneumonia; the mean (SD) within-hospital differences in RSRRs were 0.74% (1.1%) (*P* = .41) for AMI, 1.1% (1.8%) (*P* = .26) for HF, and 1.2% (1.4%) (*P* = .10) for pneumonia.

Within-hospital differences in risk-standardized mortality and readmission ratios were small (Table 2) even though some of the ICCs were lower (Figure 1B). The ICC of mortality ratios by neighborhood income was 0.46 (95% CI, 0.42-0.50) for AMI, 0.59 (95% CI, 0.56-0.62) for HF, and 0.57 (95% CI, 0.54-0.60) for pneumonia. The ICCs comparing risk-standardized readmission ratios among patients from lower-income neighborhoods were 0.60 (95% CI, 0.57-0.63) for AMI, 0.60 (95% CI, 0.57-0.63) for HF, and 0.57 (95% CI, 0.54-0.60) for pneumonia.

### Between-Hospital Performance

#### Race

**Figure 2.**
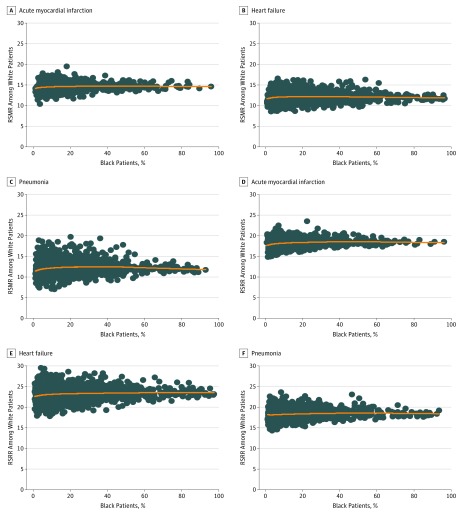
Between-Hospital Variation in Risk-Standardized Outcomes According to Race A, B, and C, Risk-standardized mortality rates (RSMRs) for acute myocardial infarction, heart failure, and pneumonia among white patients plotted against the proportion of black patients treated at each hospital for these conditions. D, E, and F, Risk-standardized readmission rates (RSRRs) for acute myocardial infarction, heart failure, and pneumonia among white patients plotted against the proportion of black patients treated at each hospital for these conditions.

Although there was variation in RSMRs between hospitals, the proportion of black patients admitted to each hospital was not associated with hospitals’ overall RSMR or that among white patients or black patients (Figure 2; eFigure 2A in the Supplement). After grouping hospitals into deciles according to the proportion of black patients treated for each condition, RSMRs among all patients, white patients, and black patients were generally consistent across deciles in most comparisons (eFigure 3A-I in the Supplement). Similarly, despite significant variation in RSRRs between hospitals, these variations were not associated with the proportion of black patients admitted to each hospital (Figure 2; eFigure 2B in the Supplement). The RSRRs for all patients and the 2 race subgroups did appear to be weakly associated with the proportion of black patients admitted to each hospital (eFigure 4A-I in the Supplement).

#### Neighborhood Income

**Figure 3.**
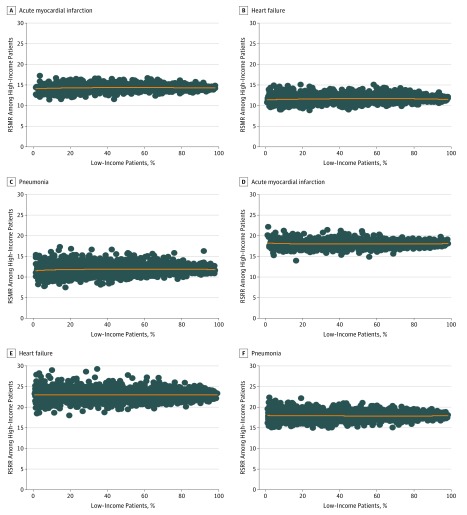
Between-Hospital Variation in Risk-Standardized Outcomes According to Patients’ Neighborhood Income A, B, and C, Risk-standardized mortality rates (RSMRs) for acute myocardial infarction, heart failure, and pneumonia among patients from higher-income neighborhoods plotted against the proportion of patients from lower-income neighborhoods treated at each hospital for these conditions. D, E, and F, Risk-standardized readmission rates (RSRRs) for acute myocardial infarction, heart failure, and pneumonia among patients from higher-income neighborhoods plotted against the proportion of patients from lower-income neighborhoods treated at each hospital for these conditions.

The proportion of patients from lower-income neighborhoods was not associated with hospitals’ overall RSMRs or that among the 2 neighborhood income subgroups (Figure 3; eFigure 5A in the Supplement). After grouping hospitals into deciles according to the proportion of patients from lower-income neighborhoods, some RSMRs for all patients, for patients from higher-income neighborhoods, and for patients from lower-income neighborhoods were weakly associated with the proportion of patients from lower-income neighborhoods (eFigure 6A-6I in the Supplement). In addition, comparisons of RSRRs between hospitals did not reveal a strong association with the proportion of patients living in lower-income neighborhoods admitted to each hospital (Figure 3; eFigure 5B in the Supplement). The RSRRs did not differ according to the proportion of patients from lower-income neighborhoods (eFigure 7A-I in the Supplement).

### Sensitivity Analysis

When hospitals treating at least 10 patients in each race and neighborhood income subgroup were added into the respective analysis cohorts, up to 40% of hospitals in the United States would be included. In these sensitivity analyses, within-hospital differences were extremely consistent with those observed in the original analysis (eTables 9 and 10 in the Supplement). Similarly, there was no evidence of meaningful between-hospital differences (eFigures 8 and 9 in the Supplement).

## Discussion

This study suggests that any differences in hospital outcomes by race and neighborhood income may be systemic, rather than localized within particular hospitals. Three key findings support this conclusion. First, we observed differences in the RSMRs and RSRRs for black patients and white patients treated for 3 common medical conditions. In the neighborhood income analyses, patients from lower-income neighborhoods tended to experience slightly worse outcomes, although few differences were significant. Second, the minimal difference in risk-standardized mortality and readmission ratios between the race and income groups, and the high ICCs, indicate that hospitals that performed better than predicted for one race and neighborhood income subgroup also did so for the other. Third, black patients and those from lower-income neighborhoods were not disproportionately concentrated in worse-performing hospitals. Together, these findings indicate relatively consistent performance within and between hospitals, thereby suggesting that systemic differences affecting all hospitals are driving the observed disparities in mortality and readmission rates.

The marked within-hospital differences in outcomes by race are not the result of individual institutions performing differently for black patients compared with white patients among our sample of hospitals. Although the risk-standardized rates are different, the ratios are similar: the interpretation is that hospital performance for white patients, relative to the performance of other hospitals for white patients, is similar to hospital performance for black patients, relative to other hospitals’ performance for black patients. Thus, the difference in rates by race appears to be a systemic issue, rather than localized to certain hospitals that perform differently for patients of different races.

We found no evidence of significant within-hospital differences in the outcomes achieved by patients from lower-income neighborhoods compared with those from higher-income neighborhoods. A persistent question is whether factors extrinsic to the hospital are responsible for observed differences in the outcomes of patients by income.^15^ However, our study does not support the community explanation, namely, that extrinsic factors drive differences in outcomes, because we did not observe significant differences in outcomes between patients with different neighborhood income groups who were treated at the same hospital. In addition, because communities are diverse across the country, the community explanation would require that black patients living in rural and urban areas be exposed to similar community issues that are not associated with income, yet influence their rates equally, independent of health care quality. An alternative explanation for these differences in outcomes is that there are patterns of care within the hospital and postdischarge environments that are similar across health care, perhaps reflecting nationwide institutional biases.

The key finding of the between-hospital analysis was that hospital performance was not consistently associated with patient mix. This analysis addressed the question of whether differences in outcomes between race and neighborhood income groups could be explained by a disproportionate number of patients in certain subgroups receiving care at high-performing or poor-performing hospitals. Prior reports suggested the possibility that this was occurring; for example, it has been observed that the care of black patients is concentrated in a relatively small number of hospitals^16^ with lower volumes,^17^ lower adherence to process measures,^18,19^ and higher risk-adjusted mortality.^20,21^ Our analysis, which modeled risk-standardized outcomes for 3 conditions using National Quality Forum–approved measures of performance, suggests that between-hospital differences, if any, are small at hospitals treating a minimum volume of patients in each race and neighborhood-income subgroup.

Lastly, we found that mortality rates for black patients were lower than those for white patients, and readmission rates were higher. The explanation for these finding is not known, although the lower mortality rates have been observed previously.^22,23,24,25^ While our study suggests that racial disparities are likely due to systemic effects, it is possible that black patients have an unmeasured predisposition for lower short-term mortality after hospitalization through a mechanism that has not yet been elucidated. Similarly, there may be a survivorship effect among our sample of Medicare patients: black patients who survive to be elderly may be healthier, on average, than white patients in ways that models are not measuring. The finding of lower mortality and higher readmission rates among black patients is consistent across the 3 common yet different conditions that we studied; thus, it cannot be explained by differential use of procedures as occurs in AMI. Interestingly, it has been shown that this early mortality advantage does not persist over the long term.^23^

### Limitations

Our study has several limitations. First, it excludes almost three-quarters of hospitals because they lacked sufficient diversity in their patient populations to allow risk-standardized outcomes to be measured in the race and neighborhood-income subgroups. While this is a limitation of the study, the exclusion of so many hospitals highlights the relatively segregated nature of hospital care in the United States. Nonetheless, our results were extremely consistent in sensitivity analyses that allowed the inclusion of hospitals treating as few as 10 patients in each race and neighborhood income subgroup. Additionally, the question of whether there are variations in quality according to patients’ race and neighborhood income group at hospitals excluded from our principal analysis, which tended to be smaller and more homogeneous, is not particularly germane. Second, our measures of neighborhood income are based on estimates at the level of zip codes, which is a relatively crude approximation of socioeconomic status. However, at the hospital level, it is a reasonable assumption that our approach can produce 2 groups that should differ markedly in their socioeconomic status; prior studies have used similar approaches in the absence of individual patient-level data.^26,27,28^ Third, national performance measures are based on administrative claims data. More information on disease severity and functional status might have influenced our findings. Fourth, our analysis focused on the quality of care for 3 common conditions (AMI, HF, and pneumonia) and the Medicare population; it is not clear whether our findings could be broadly applicable to other conditions or to younger patients. While we cannot say whether our findings apply universally across hospitals of any size, it is not possible, with any statistical accuracy, to measure disparities in hospital outcomes in smaller hospitals using current quality measures.

## Conclusions

We found evidence that differences in the performance of hospitals in the United States according to patients’ race and neighborhood-income level may be systemic. Consequently, initiatives seeking to address these differences likely will require far-reaching interventions in and out of the health care system.

## Notes

Supplement. **eTable 1.** Comparison of Characteristics of Hospitals Included in the Analysis of Mortality After Admission for Myocardial Infarction, Heart Failure, and Pneumonia by Race **eTable 2.** Comparison of Characteristics of Hospitals Included in the Analysis of Mortality After Admission for Myocardial Infarction, Heart Failure, and Pneumonia by Neighborhood Income **eTable 3.** Comparison of Characteristics of Hospitals Included in the Analysis of Readmission After Admission for Myocardial Infarction, Heart Failure, and Pneumonia by Race **eTable 4.** Comparison of Characteristics of Hospitals Included in the Analysis of Readmission After Admission for Myocardial Infarction, Heart Failure, and Pneumonia by Neighborhood Income **eTable 5.** Patient Characteristics Among Hospitals Included in Mortality Analyses by Race **eTable 6.** Patient Characteristics Among Hospitals Included in Readmission Analyses by Race **eTable 7.** Patient Characteristics Among Hospitals Included in Mortality Analyses **eTable 8.** Patient Characteristics Among Hospitals Included in Readmission Analyses by Neighborhood Income **eTable 9.** Within-Hospital Differences in Risk-Standardized Mortality and Readmission Rates in a Sensitivity Analysis in Which Any Hospital With at Least 10 Patients in Each Race or Neighborhood Income Subgroup Was Included **eTable 10.** Within-Hospital Differences in Risk-Standardized Mortality and Readmission Ratios in a Sensitivity Analysis in Which Any Hospital With at Least 10 Patients in Each Race or Neighborhood Income Subgroup Was Included **eFigure 1.** Approach to Identification of Mortality and Readmission Cohorts for Each of the 3 Conditions of Interest: Acute Myocardial Infarction, Heart Failure, and Pneumonia **eFigure 2.** Scatterplots Showing Between-Hospital Variation in Risk-Standardized Mortality Rates (RSMRs) and Risk-Standardized Readmission Rates (RSRRs) According to Race. Hospital RSMRs and RSRRs for Acute Myocardial Infarction (AMI), Heart Failure, and Pneumonia Among All Patients and Among Black Patients Are Plotted Against the Proportion of Black Patients Treated at Each Hospital for These Conditions **eFigure 3.** Risk-Standardized Mortality Rates (RSMRs) for All Patients, White Patients and Black Patients Treated for Acute Myocardial Infarction, Heart Failure and Pneumonia by Decile of the Proportion of Black Patients Treated at Each Hospital **eFigure 4.** Risk-Standardized Readmission Rates (RSRRs) for All Patients, White Patients and Black Patients Treated for Acute Myocardial Infarction, Heart Failure and Pneumonia by Decile of the Proportion of Black Patients Treated at Each Hospital **eFigure 5.** Scatterplots Showing Between-Hospital Variation in Risk-Standardized Mortality Rates (RSMRs) and Risk-Standardized Readmission Rates (RSRRs) According to Neighborhood Income **eFigure 6.** Risk-Standardized Mortality Rates (RSMRs) for All Patients, Patients From Higher-Income Neighborhoods and Patients From Lower-Income Neighborhoods Treated for Acute Myocardial Infarction, Heart Failure and Pneumonia by Decile of the Proportion of Patients From Lower-Income Neighborhoods Treated at Each Hospital **eFigure 7.** Risk-Standardized Readmission Rates (RSRRs) for All Patients, Patients From Higher-Income Neighborhoods, and Patients From Lower-Income Neighborhoods Treated for Acute Myocardial Infarction, Heart Failure and Pneumonia by Decile of the Proportion of Patients From Lower-Income Neighborhoods Treated at Each Hospital **eFigure 8.** Between-Hospital Differences in Risk-Standardized Mortality and Readmission Rates According to Race in a Sensitivity Analysis in Which Any Hospital With at Least 10 Eligible White Patients and 10 Eligible Black Patients Was Included **eFigure 9.** Between-Hospital Differences in Risk-Standardized Mortality and Readmission Rates According to Neighborhood Income in a Sensitivity Analysis in Which Any Hospital With at Least 10 Eligible Patients From Higher-Income Neighborhoods and 10 Eligible Patients From Lower-Income Neighborhoods Was Included

## References

[1] Institute of Medicine Crossing the Quality Chasm: A New Health System for the 21st Century. Washington, DC: National Academies Press; 2001. 25057539

[2] Institute of Medicine How Far Have We Come in Reducing Health Disparities? Progress Since 2000. Washington, DC: National Academies Press; 2012. 23193624

[3] Centers for Medicare & Medicaid Services Medicare hospital quality chartbook: performance report on outcome measures. https://www.cms.gov/medicare/quality-initiatives-patient-assessment-instruments/hospitalqualityinits/downloads/medicare-hospital-quality-chartbook-2014.pdf. Published September 2014. Accessed May 25, 2018.

[4] Schmaltz SP, Williams SC, Chassin MR, Loeb JM, Wachter RM Hospital performance trends on national quality measures and the association with Joint Commission accreditation. J Hosp Med. 2011;6(8):-. doi:10.1002/jhm.905 21990175 PMC3265714

[5] The Joint Commission *America's Hospitals: Improving Quality and Safety* https://www.jointcommission.org/assets/1/18/TJC_Annual_Report_2015_EMBARGOED_11_9_15.pdf. Published 2015. Accessed May 25, 2018.

[6] Bratzler DW, Normand SL, Wang Y, An administrative claims model for profiling hospital 30-day mortality rates for pneumonia patients. PLoS One. 2011;6(4):e17401. doi:10.1371/journal.pone.0017401 21532758 PMC3075250

[7] Keenan PS, Normand SL, Lin Z, An administrative claims measure suitable for profiling hospital performance on the basis of 30-day all-cause readmission rates among patients with heart failure. Circ Cardiovasc Qual Outcomes. 2008;1(1):29-37. doi:10.1161/CIRCOUTCOMES.108.802686 20031785

[8] Krumholz HM, Lin Z, Drye EE, An administrative claims measure suitable for profiling hospital performance based on 30-day all-cause readmission rates among patients with acute myocardial infarction. Circ Cardiovasc Qual Outcomes. 2011;4(2):243-252. doi:10.1161/CIRCOUTCOMES.110.957498 21406673 PMC3350811

[9] Krumholz HM, Wang Y, Mattera JA, An administrative claims model suitable for profiling hospital performance based on 30-day mortality rates among patients with an acute myocardial infarction. Circulation. 2006;113(13):1683-1692. doi:10.1161/CIRCULATIONAHA.105.611186 16549637

[10] Lindenauer PK, Normand SL, Drye EE, Development, validation, and results of a measure of 30-day readmission following hospitalization for pneumonia. J Hosp Med. 2011;6(3):142-150. doi:10.1002/jhm.890 21387551

[11] Krumholz HM, Wang Y, Mattera JA, An administrative claims model suitable for profiling hospital performance based on 30-day mortality rates among patients with heart failure. Circulation. 2006;113(13):1693-1701. doi:10.1161/CIRCULATIONAHA.105.611194 16549636

[12] US Census Bureau 2011 American Community Survey 5-year estimates, table S1903. https://factfinder2.census.gov. Accessed May 25, 2018.

[13] Bradley EH, Herrin J, Wang Y, Racial and ethnic differences in time to acute reperfusion therapy for patients hospitalized with myocardial infarction. JAMA. 2004;292(13):1563-1572. doi:10.1001/jama.292.13.1563 15467058

[14] American Hospital Association 2010 American Hospital Association annual survey of hospitals. http://www.ahadata.com/aha-annual-survey-database-asdb/. Accessed May 29, 2018.

[15] National Quality Forum Risk adjustment for socioeconomic status or other sociodemographic factors—technical report. http://www.qualityforum.org/Publications/2014/08/Risk_Adjustment_for_Socioeconomic_Status_or_Other_Sociodemographic_Factors.aspx. Published August 15, 2014. Accessed May 25, 2018.

[16] Jha AK, Orav EJ, Li Z, Epstein AM Concentration and quality of hospitals that care for elderly black patients. Arch Intern Med. 2007;167(11):1177-1182. doi:10.1001/archinte.167.11.1177 17563027

[17] Trivedi AN, Sequist TD, Ayanian JZ Impact of hospital volume on racial disparities in cardiovascular procedure mortality. J Am Coll Cardiol. 2006;47(2):417-424. doi:10.1016/j.jacc.2005.08.068 16412871

[18] Hasnain-Wynia R, Baker DW, Nerenz D, Disparities in health care are driven by where minority patients seek care: examination of the hospital quality alliance measures. Arch Intern Med. 2007;167(12):1233-1239. doi:10.1001/archinte.167.12.1233 17592095

[19] Trivedi AN, Nsa W, Hausmann LRM, Quality and equity of care in U.S. hospitals. N Engl J Med. 2014;371(24):2298-2308. doi:10.1056/NEJMsa1405003 25494269

[20] Haider AH, Ong’uti S, Efron DT, Association between hospitals caring for a disproportionately high percentage of minority trauma patients and increased mortality: a nationwide analysis of 434 hospitals. Arch Surg. 2012;147(1):63-70. doi:10.1001/archsurg.2011.254 21930976 PMC3684151

[21] Skinner J, Chandra A, Staiger D, Lee J, McClellan M Mortality after acute myocardial infarction in hospitals that disproportionately treat black patients. Circulation. 2005;112(17):2634-2641. doi:10.1161/CIRCULATIONAHA.105.543231 16246963 PMC1626584

[22] Barnato AE, Lucas FL, Staiger D, Wennberg DE, Chandra A Hospital-level racial disparities in acute myocardial infarction treatment and outcomes. Med Care. 2005;43(4):308-319. doi:10.1097/01.mlr.0000156848.62086.06 15778634 PMC2121607

[23] Polsky D, Jha AK, Lave J, Short- and long-term mortality after an acute illness for elderly whites and blacks. Health Serv Res. 2008;43(4):1388-1402. doi:10.1111/j.1475-6773.2008.00837.x 18355259 PMC2517279

[24] Polsky D, Lave J, Klusaritz H, Is lower 30-day mortality posthospital admission among blacks unique to the Veterans Affairs health care system? Med Care. 2007;45(11):1083-1089. doi:10.1097/MLR.0b013e3180ca960e 18049349

[25] Thomas KL, Hernandez AF, Dai D, Association of race/ethnicity with clinical risk factors, quality of care, and acute outcomes in patients hospitalized with heart failure. Am Heart J. 2011;161(4):746-754. doi:10.1016/j.ahj.2011.01.012 21473975

[26] Kimmel PL, Fwu CW, Eggers PW Segregation, income disparities, and survival in hemodialysis patients. J Am Soc Nephrol. 2013;24(2):293-301. doi:10.1681/ASN.2012070659 23334394 PMC3559484

[27] Woodward RS, Page TF, Soares R, Schnitzler MA, Lentine KL, Brennan DC Income-related disparities in kidney transplant graft failures are eliminated by Medicare’s immunosuppression coverage. Am J Transplant. 2008;8(12):2636-2646. doi:10.1111/j.1600-6143.2008.02422.x 19032227 PMC3189683

[28] Yoo HY, Thuluvath PJ Outcome of liver transplantation in adult recipients: influence of neighborhood income, education, and insurance. Liver Transpl. 2004;10(2):235-243. doi:10.1002/lt.20069 14762861

