# Supplementary material for: Association of Racial and Socioeconomic Disparities With Outcomes Among Patients Hospitalized With Acute Myocardial Infarction, Heart Failure, and Pneumonia: An Analysis of Within- and Between-Hospital Variation
Source: JAMA Netw Open. 2018 Sep 7;1(5):e182044. doi: 10.1001/jamanetworkopen.2018.2044 (PMC6324513; doi:10.1001/jamanetworkopen.2018.2044)
Supplement: Supplement. — eTable 1. Comparison of Characteristics of Hospitals Included in the Analysis of Mortality After Admission for Myocardial Infarction, Heart Failure, and Pneumonia by Race eTable 2. Comparison of Characteristics of Hospitals Included in the Analysis of Mortality After Admission for Myocardial Infarction, Heart Failure, and Pneumonia by Neighborhood Income eTable 3. Comparison of Characteristics of Hospitals Included in the Analysis of Readmission After Admission for Myocardial Infarction, Heart Failure, and Pneumonia by Race eTable 4. Comparison of Characteristics of Hospitals Included in the Analysis of Readmission After Admission for Myocardial Infarction, Heart Failure, and Pneumonia by Neighborhood Income eTable 5. Patient Characteristics Among Hospitals Included in Mortality Analyses by Race eTable 6. Patient Characteristics Among Hospitals Included in Readmission Analyses by Race eTable 7. Patient Characteristics Among Hospitals Included in Mortality Analyses eTable 8. Patient Characteristics Among Hospitals Included in Readmission Analyses by Neighborhood Income eTable 9. Within-Hospital Differences in Risk-Standardized Mortality and Readmission Rates in a Sensitivity Analysis in Which Any Hospital With at Least 10 Patients in Each Race or Neighborhood Income Subgroup Was Included eTable 10. Within-Hospital Differences in Risk-Standardized Mortality and Readmission Ratios in a Sensitivity Analysis in Which Any Hospital With at Least 10 Patients in Each Race or Neighborhood Income Subgroup Was Included eFigure 1. Approach to Identification of Mortality and Readmission Cohorts for Each of the 3 Conditions of Interest: Acute Myocardial Infarction, Heart Failure, and Pneumonia eFigure 2. Scatterplots Showing Between-Hospital Variation in Risk-Standardized Mortality Rates (RSMRs) and Risk-Standardized Readmission Rates (RSRRs) According to Race. Hospital RSMRs and RSRRs for Acute Myocardial Infarction (AMI), Heart Failure, and Pneumonia Among All Patients and Among [file jamanetwopen-1-e182044-s001.pdf]

Supplementary Online Content

Downing NS, Wang C, Gupta A, et al. Association of Racial and Socioeconomic Disparities With Outcomes Among Patients Hospitalized With Acute Myocardial Infarction, Heart Failure, and Pneumonia: An Analysis of Within- and Between-Hospital Variation. *JAMA Netw Open.* 2018;1(5):e182044. doi:10.1001/jamanetworkopen.2018.2044

eTable 1. Comparison of Characteristics of Hospitals Included in the Analysis of Mortality After Admission for Myocardial Infarction, Heart Failure, and Pneumonia by Race

eTable 2. Comparison of Characteristics of Hospitals Included in the Analysis of Mortality After Admission for Myocardial Infarction, Heart Failure, and Pneumonia by Neighborhood Income

eTable 3. Comparison of Characteristics of Hospitals Included in the Analysis of Readmission After Admission for Myocardial Infarction, Heart Failure, and Pneumonia by Race

eTable 4. Comparison of Characteristics of Hospitals Included in the Analysis of Readmission After Admission for Myocardial Infarction, Heart Failure, and Pneumonia by Neighborhood Income

eTable 5. Patient Characteristics Among Hospitals Included in Mortality Analyses by Race

eTable 6. Patient Characteristics Among Hospitals Included in Readmission Analyses by Race

eTable 7. Patient Characteristics Among Hospitals Included in Mortality Analyses

eTable 8. Patient Characteristics Among Hospitals Included in Readmission Analyses by Neighborhood Income

eTable 9. Within-Hospital Differences in Risk-Standardized Mortality and Readmission Rates in a Sensitivity Analysis in Which Any Hospital With at Least 10 Patients in Each Race or Neighborhood Income Subgroup Was Included

eTable 10. Within-Hospital Differences in Risk-Standardized Mortality and Readmission Ratios in a Sensitivity Analysis in Which Any Hospital With at Least 10 Patients in Each Race or Neighborhood Income Subgroup Was Included

eFigure 1. Approach to Identification of Mortality and Readmission Cohorts for Each of the 3 Conditions of Interest: Acute Myocardial Infarction, Heart Failure, and Pneumonia

eFigure 2. Scatterplots Showing Between-Hospital Variation in Risk-Standardized Mortality Rates (RSMRs) and Risk-Standardized Readmission Rates (RSRRs) According to Race. Hospital RSMRs and RSRRs for Acute Myocardial Infarction (AMI), Heart Failure, and Pneumonia Among All Patients and Among Black Patients Are Plotted Against the Proportion of Black Patients Treated at Each Hospital for These Conditions

eFigure 3. Risk-Standardized Mortality Rates (RSMRs) for All Patients, White Patients and Black Patients Treated for Acute Myocardial Infarction, Heart Failure and Pneumonia by Decile of the Proportion of Black Patients Treated at Each Hospital

© 2018 Downing NS et al. *JAMA Network Open.*

eFigure 4. Risk-Standardized Readmission Rates (RSRRs) for All Patients, White Patients and Black Patients Treated for Acute Myocardial Infarction, Heart Failure and Pneumonia by Decile of the Proportion of Black Patients Treated at Each Hospital

eFigure 5. Scatterplots Showing Between-Hospital Variation in Risk-Standardized Mortality Rates (RSMRs) and Risk-Standardized Readmission Rates (RSRRs) According to Neighborhood Income

eFigure 6. Risk-Standardized Mortality Rates (RSMRs) for All Patients, Patients From Higher-Income Neighborhoods and Patients From Lower-Income Neighborhoods Treated for Acute Myocardial Infarction, Heart Failure and Pneumonia by Decile of the Proportion of Patients From Lower-Income Neighborhoods Treated at Each Hospital

eFigure 7. Risk-Standardized Readmission Rates (RSRRs) for All Patients, Patients From Higher-Income Neighborhoods, and Patients From Lower-Income Neighborhoods Treated for Acute Myocardial Infarction, Heart Failure and Pneumonia by Decile of the Proportion of Patients From Lower-Income Neighborhoods Treated at Each Hospital

eFigure 8. Between-Hospital Differences in Risk-Standardized Mortality and Readmission Rates According to Race in a Sensitivity Analysis in Which Any Hospital With at Least 10 Eligible White Patients and 10 Eligible Black Patients Was Included

eFigure 9. Between-Hospital Differences in Risk-Standardized Mortality and Readmission Rates According to Neighborhood Income in a Sensitivity Analysis in Which Any Hospital With at Least 10 Eligible Patients From Higher-Income Neighborhoods and 10 Eligible Patients From Lower-Income Neighborhoods Was Included

This supplementary material has been provided by the authors to give readers additional information about their work.

Appendix Table 1. Comparison of characteristics of hospitals included in the analysis of mortality after admission for myocardial infarction, heart failure, and pneumonia by race.

Panel A: Myocardial infarction

	All (4591)		Excluded (4173)		Included (418)	
**Region**	#	%	#	%	#	%
Missing	258	5.62	244	5.85	14	3.35
Northeast	566	12.33	500	11.98	66	15.79
Midwest	1307	28.47	1230	29.48	77	18.42
South	1635	35.61	1392	33.36	243	58.13
West	825	17.97	807	19.34	18	4.31
**Teaching status**	#	%	#	%	#	%
Missing	210	4.57	196	4.70	14	3.35
Non-teaching	3573	77.83	3410	81.72	163	39.00
Teaching	808	17.60	567	13.59	241	57.66
**Number of beds**	#	%	#	%	#	%
Missing	210	4.57	196	4.70	14	3.35
<300	3591	78.22	3479	83.37	112	26.79
300 to <600	617	13.44	427	10.23	190	45.45
>=600	173	3.77	71	1.70	102	24.40
**30-day mortality rates**	*Mean*	*SD*	*Mean*	*SD*	*Mean*	*SD*
Observed	22.0%	17.4%	22.7%	18.1%	14.7%	3.4%
Risk-standardized	15.5%	1.1%	15.5%	1.0%	15.1%	1.5%

Panel B: Heart failure

	All (4800)		Excluded (3723)		Included (1077)	
**Region**	#	%	#	%	#	%
Missing	285	5.94	218	5.86	67	6.22
Northeast	571	11.90	404	10.85	167	15.51
Midwest	1360	28.33	1173	31.51	187	17.36
South	1702	35.46	1117	30.00	585	54.32
West	882	18.38	811	21.78	71	6.59
**Teaching status**	#	%	#	%	#	%
Missing	234	4.88	167	4.49	67	6.22
Non-teaching	3747	78.06	3179	85.39	568	52.74
Teaching	819	17.06	377	10.13	442	41.04
**Number of beds**	#	%	#	%	#	%
Missing	234	4.88	167	4.49	67	6.22
<300	3771	78.56	3261	87.59	510	47.35
300 to <600	622	12.96	269	7.23	353	32.78
>=600	173	3.60	26	0.70	147	13.65
**30-day mortality rates**	*Mean*	*SD*	*Mean*	SD	Mean	SD
Observed	12.5%	6.6%	12.9%	7.2%	10.9%	2.7%
Risk-standardized	11.8%	1.4%	11.9%	1.3%	11.3%	1.6%

© 2018 Downing NS et al. *JAMA Network Open.*

Panel C: Pneumonia

	All (4834)		Excluded (3994)		Included (840)	
**Region**	#	%	#	%	#	%
Missing	290	6.00	238	5.96	52	6.19
Northeast	576	11.92	458	11.47	118	14.05
Midwest	1362	28.18	1209	30.27	153	18.21
South	1710	35.37	1227	30.72	483	57.50
West	896	18.54	862	21.58	34	4.05
**Teaching status**	#	%	#	%	#	%
Missing	240	4.96	189	4.73	51	6.07
Non-teaching	3771	78.01	3333	83.45	438	52.14
Teaching	823	17.03	472	11.82	351	41.79
**Number of beds**	#	%	#	%	#	%
Missing	240	4.96	189	4.73	51	6.07
<300	3796	78.53	3424	85.73	372	44.29
300 to <600	624	12.91	333	8.34	291	34.64
>=600	174	3.60	48	1.20	126	15.00
**30-day mortality rates**	*Mean*	*SD*	*Mean*	*SD*	*Mean*	*SD*
Observed	12.0%	5.6%	11.9%	6.0%	12.3%	3.0%
Risk-standardized	12.1%	1.7%	12.1%	1.6%	12.0%	1.9%

© 2018 Downing NS et al. *JAMA Network Open.*

Appendix Table 2. Comparison of characteristics of hospitals included in the analysis of mortality after admission for myocardial infarction, heart failure, and pneumonia by neighborhood income.

Panel A: Myocardial infarction

	All (4591)		Excluded (3850)		Included (741)	
**Region**	#	%	#	%	#	%
Missing	258	5.62	231	6.00	27	3.64
Northeast	566	12.33	452	11.74	114	15.38
Midwest	1307	28.47	1105	28.70	202	27.26
South	1635	35.61	1345	34.94	290	39.14
West	825	17.97	717	18.62	108	14.57
**Teaching status**	#	%	#	%	#	%
Missing	210	4.57	183	4.75	27	3.64
Non-teaching	3573	77.83	3220	83.64	353	47.64
Teaching	808	17.60	447	11.61	361	48.72
**Number of beds**	#	%	#	%	#	%
Missing	210	4.57	183	4.75	27	3.64
<300	3591	78.22	3324	86.34	267	36.03
300 to <600	617	13.44	304	7.90	313	42.24
>=600	173	3.77	39	1.01	134	18.08
**30-day mortality rates**	*Mean*	*SD*	*Mean*	*SD*	*Mean*	*SD*
Observed	22.0%	17.4%	23.5%	18.6%	14.3%	3.3%
Risk-standardized	15.5%	1.1%	15.5%	1.0%	15.0%	1.5%

Panel B: Heart failure

	All (4800)		Excluded (3653)		Included (1147)	
**Region**	#	%	#	%	#	%
Missing	285	5.94	211	5.78	74	6.45
Northeast	571	11.90	382	10.46	189	16.48
Midwest	1360	28.33	1068	29.24	292	25.46
South	1702	35.46	1268	34.71	434	37.84
West	882	18.38	724	19.82	158	13.78
**Teaching status**	#	%	#	%	#	%
Missing	234	4.88	160	4.38	74	6.45
Non-teaching	3747	78.06	3155	86.37	592	51.61
Teaching	819	17.06	338	9.25	481	41.94
**Number of beds**	#	%	#	%	#	%
Missing	234	4.88	160	4.38	74	6.45
<300	3771	78.56	3247	88.89	524	45.68
300 to <600	622	12.96	222	6.08	400	34.87
>=600	173	3.60	24	0.66	149	12.99
**30-day mortality rates**	*Mean*	*SD*	*Mean*	*SD*	*Mean*	*SD*
Observed	12.5%	6.6%	12.8%	7.3%	11.3%	2.7%
Risk-standardized	11.8%	1.4%	11.9%	1.3%	11.4%	1.7%

© 2018 Downing NS et al. *JAMA Network Open.*

Panel C: Pneumonia

	All (4834)		Excluded (3674)		Included (1160)	
**Region**	#	%	#	%	#	%
Missing	290	6.00	214	5.82	76	6.55
Northeast	576	11.92	396	10.78	180	15.52
Midwest	1362	28.18	1072	29.18	290	25.00
South	1710	35.37	1263	34.38	447	38.53
West	896	18.54	729	19.84	167	14.40
**Teaching status**	#	%	#	%	#	%
Missing	240	4.96	164	4.46	76	6.55
Non-teaching	3771	78.01	3152	85.79	619	53.36
Teaching	823	17.03	358	9.74	465	40.09
**Number of beds**	#	%	#	%	#	%
Missing	240	4.96	164	4.46	76	6.55
<300	3796	78.53	3247	88.38	549	47.33
300 to <600	624	12.91	235	6.40	389	33.53
>=600	174	3.60	28	0.76	146	12.59
**30-day mortality rates**	*Mean*	*SD*	*Mean*	*SD*	*Mean*	*SD*
Observed	12.0%	5.6%	12.0%	6.3%	12.0%	2.8%
Risk-standardized	12.1%	1.7%	12.2%	1.6%	11.8%	1.8%

© 2018 Downing NS et al. *JAMA Network Open.*

Appendix Table 3. Comparison of characteristics of hospitals included in the analysis of readmission after admission for myocardial infarction, heart failure, and pneumonia by race.

Panel A: Myocardial infarction

	All (4492)		Excluded (4068)		Included (424)	
**Region**	#	%	#	%	#	%
Missing	251	5.59	235	5.78	16	3.77
Northeast	563	12.53	493	12.12	70	16.51
Midwest	1277	28.43	1200	29.50	77	18.16
South	1596	35.53	1355	33.31	241	56.84
West	805	17.92	785	19.30	20	4.72
**Teaching status**	#	%	#	%	#	%
Missing	203	4.52	187	4.60	16	3.77
Non-teaching	3485	77.58	3330	81.86	155	36.56
Teaching	804	17.90	551	13.54	253	59.67
**Number of beds**	#	%	#	%	#	%
Missing	203	4.52	187	4.60	16	3.77
<300	3499	77.89	3398	83.53	101	23.82
300 to <600	617	13.74	421	10.35	196	46.23
>=600	173	3.85	62	1.52	111	26.18
**30-day readmission rates**	*Mean*	*SD*	*Mean*	*SD*	*Mean*	*SD*
Observed	19.2%	14.1%	19.1%	14.7%	20.0%	4.4%
Risk-standardized	18.5%	1.0%	18.5%	0.9%	19.0%	1.5%

Panel B: Heart failure

	All (4812)		Excluded (3555)		Included (1257)	
**Region**	#	%	#	%	#	%
Missing	289	6.01	212	5.96	77	6.13
Northeast	572	11.89	379	10.66	193	15.35
Midwest	1363	28.33	1152	32.41	211	16.79
South	1703	35.39	1024	28.80	679	54.02
West	885	18.39	788	22.17	97	7.72
**Teaching status**	#	%	#	%	#	%
Missing	238	4.95	161	4.53	77	6.13
Non-teaching	3754	78.01	3061	86.10	693	55.13
Teaching	820	17.04	333	9.37	487	38.74
**Number of beds**	#	%	#	%	#	%
Missing	238	4.95	161	4.53	77	6.13
<300	3779	78.53	3146	88.50	633	50.36
300 to <600	62200.0%	1292.6%	23000.0%	647.0%	39200.0%	3118.5%
>=600	17300.0%	359.5%	1800.0%	50.6%	15500.0%	1233.1%
**30-day readmission rates**	*Mean*	*SD*	*Mean*	*SD*	*Mean*	*SD*
Observed	22.1%	7.9%	21.4%	8.8%	24.0%	3.8%
Risk-standardized	23.2%	1.7%	23.1%	1.6%	23.6%	2.1%

© 2018 Downing NS et al. *JAMA Network Open.*

Panel C: Pneumonia

	All (4846)		Excluded (3939)		Included (907)	
**Region**	#	%	#	%	#	%
Missing	299	6.17	245	6.22	54	5.95
Northeast	576	11.89	448	11.37	128	14.11
Midwest	1362	28.11	1199	30.44	163	17.97
South	1711	35.31	1192	30.26	519	57.22
West	898	18.53	855	21.71	43	4.74
**Teaching status**	#	%	#	%	#	%
Missing	249	5.14	196	4.98	53	5.84
Non-teaching	3775	77.90	3294	83.63	481	53.03
Teaching	822	16.96	449	11.40	373	41.12
**Number of beds**	#	%	#	%	#	%
Missing	249	5.14	196	4.98	53	5.84
<300	3799	78.39	3380	85.81	419	46.20
300 to <600	624	12.88	321	8.15	303	33.41
>=600	174	3.59	42	1.07	132	14.55
**30-day readmission rates**	*Mean*	*SD*	*Mean*	*SD*	*Mean*	*SD*
Observed	16.6%	6.3%	16.0%	6.6%	19.1%	3.7%
Risk-standardized	17.7%	1.4%	17.6%	1.3%	18.4%	1.6%

© 2018 Downing NS et al. *JAMA Network Open.*

Appendix Table 4. Comparison of characteristics of hospitals included in the analysis of readmission after admission for myocardial infarction, heart failure, and pneumonia by neighborhood income.

Panel A: Myocardial infarction

	All (4492)		Excluded (3728)		Included (764)	
**Region**	#	%	#	%	#	%
Missing	251	5.59	219	5.87	32	4.19
Northeast	563	12.53	447	11.99	116	15.18
Midwest	1277	28.43	1071	28.73	206	26.96
South	1596	35.53	1305	35.01	291	38.09
West	805	17.92	686	18.40	119	15.58
**Teaching status**	#	%	#	%	#	%
Missing	203	4.52	171	4.59	32	4.19
Non-teaching	3485	77.58	3137	84.15	348	45.55
Teaching	804	17.90	420	11.27	384	50.26
**Number of beds**	#	%	#	%	#	%
Missing	203	4.52	171	4.59	32	4.19
<300	3499	77.89	3237	86.83	262	34.29
300 to <600	617	13.74	288	7.73	329	43.06
>=600	173	3.85	32	0.86	141	18.46
**30-day readmission rates**	*Mean*	*SD*	*Mean*	*SD*	*Mean*	*SD*
Observed	19.2%	14.1%	19.4%	15.3%	18.2%	4.2%
Risk-standardized	18.5%	1.0%	18.5%	0.8%	18.5%	1.6%

Panel B: Heart failure

	All (4812)		Excluded (3547)		Included (1265)	
**Region**	#	%	#	%	#	%
Missing	289	6.01	203	5.72	86	6.80
Northeast	572	11.89	362	10.21	210	16.60
Midwest	1363	28.33	1045	29.46	318	25.14
South	1703	35.39	1227	34.59	476	37.63
West	885	18.39	710	20.02	175	13.83
**Teaching status**	#	%	#	%	#	%
Missing	238	4.95	152	4.29	86	6.80
Non-teaching	3754	78.01	3086	87.00	668	52.81
Teaching	820	17.04	309	8.71	511	40.40
**Number of beds**	#	%	#	%	#	%
Missing	238	4.95	152	4.29	86	6.80
<300	3779	78.53	3179	89.63	600	47.43
300 to <600	622	12.93	198	5.58	424	33.52
>=600	173	3.60	18	0.51	155	12.25
**30-day readmission rates**	*Mean*	*SD*	*Mean*	*SD*	*Mean*	*SD*
Observed	22.1%	7.9%	21.6%	8.9%	23.3%	3.7%
Risk-standardized	23.2%	1.7%	23.2%	1.6%	23.2%	2.0%

© 2018 Downing NS et al. *JAMA Network Open.*

Panel C: Pneumonia

	All (4846)		Excluded (3619)		Included (1227)	
**Region**	#	%	#	%	#	%
Missing	299	6.17	219	6.05	80	6.52
Northeast	576	11.89	392	10.83	184	15.00
Midwest	1362	28.11	1058	29.23	304	24.78
South	1711	35.31	1237	34.18	474	38.63
West	898	18.53	713	19.70	185	15.08
**Teaching status**	#	%	#	%	#	%
Missing	249	5.14	169	4.67	80	6.52
Non-teaching	3775	77.90	3109	85.91	666	54.28
Teaching	822	16.96	341	9.42	481	39.20
**Number of beds**	#	%	#	%	#	%
Missing	249	5.14	169	4.67	80	6.52
<300	3799	78.39	3202	88.48	597	48.66
300 to <600	624	12.88	221	6.11	403	32.84
>=600	174	3.59	27	0.75	147	11.98
**30-day readmission rates**	*Mean*	*SD*	*Mean*	*SD*	*Mean*	*SD*
Observed	16.6%	6.3%	16.0%	6.9%	18.4%	3.7%
Risk-standardized	17.7%	1.4%	17.6%	1.3%	18.0%	1.6%

© 2018 Downing NS et al. *JAMA Network Open.*

Appendix Table 5. Patient characteristics among hospitals included in mortality analyses by race.

	Acute myocardial infarction				Heart failure				Pneumonia			
	All	White	Black	p	All	White	Black	p	All	White	Black	p
**Admissions - n (%)**	144417 (100%)	119570 (82.7%)	24847 (17.3%)	n/a	507799 (100%)	409572 (80.7%)	98227 (19.3%)	n/a	335659 (100%)	283140 (84.4%)	52519 (15.6%)	n/a
**Demographics**												
Age(SD)	78.4(8.1)	78.7(8.1)	77.4(8.3)	<0.0001	80.6(8.2)	81.3(8.0)	77.8(8.3)	<0.0001	80.0(8.3)	80.3(8.2)	78.4(8.6)	<0.0001
Male (%)	50.57	52.44	41.56	<0.0001	45.68	46.84	40.84	<0.0001	45.69	46.08	43.6	<0.0001
**Medical history (%)***												
Acute myocardial infarction†	14.08	13.58	16.49	<0.0001	9.82	9.88	9.56	0.0025	3.99	3.88	4.6	<0.0001
Congestive heart failure	31.08	28.93	41.41	<0.0001	74.89	74.18	77.83	<0.0001	39.03	38.18	43.63	<0.0001
Pneumonia	23.06	22.55	25.54	<0.0001	43.38	44.04	40.63	<0.0001	42.65	42.51	43.44	<0.0001
Coronary artery bypass grafting	7.49	7.82	5.86	<0.0001	12.37	13.59	7.26	<0.0001	6.15	6.68	3.29	<0.0001
Percutaneous coronary intervention	10.95	11.13	10.03	<0.0001	8.57	8.98	6.87	<0.0001	4.75	5.04	3.2	<0.0001
**Comorbidities (%)**												
Diabetes	45.51	42.8	58.54	<0.0001	52.91	50.4	63.4	<0.0001	n/a	n/a	n/a	n/a
Valvular and rheumatic heart disease	28.01	28.01	27.99	0.9514	48.59	50.01	42.67	<0.0001	n/a	n/a	n/a	n/a
Stroke	8.36	7.51	12.43	<0.0001	10.68	10.18	12.8	<0.0001	11.04	10.16	15.8	<0.0001
Renal failure	24.38	21.53	38.14	<0.0001	46.23	43.86	56.15	<0.0001	28.37	26.11	40.53	<0.0001
Asthma	n/a	n/a	n/a	n/a	n/a	n/a	n/a	n/a	11.08	10.75	12.86	<0.0001
COPD	28.01	28.37	26.25	<0.0001	45.72	46.5	42.48	<0.0001	52.62	53.98	45.32	<0.0001
Other chronic lung disease	n/a	n/a	n/a	n/a	n/a	n/a	n/a	n/a	16.68	17.19	13.91	<0.0001
Lung cancer, metastatic cancer and acute leukemia†	4.02	3.98	4.19	0.1202	4.58	4.68	4.14	<0.0001	10.68	10.6	11.08	0.001
Dementia	18.48	17.32	24.1	<0.0001	22.81	22.62	23.61	<0.0001	30.62	29.62	35.99	<0.0001
Major psychiatric disorder	6.88	6.72	7.67	<0.0001	9.61	9.8	8.8	<0.0001	12.87	12.92	12.65	0.0919

* i.e., prior to current admission

† Medical history and comorbidity variables reflect defintion used in the models predicting mortality/readmission. While the vast majority of variables are defined similarly in these models, there are some differences and two such differences are shown in these tables: 1) acute myocardial infarction alone is a predictor in the mortality models, while acute coronary syndrome (i.e., the composite of acute myocardial infarction and unstable angina) is the corresponding predictor in the readmission models. 2) the composite of lung cancer, cancer of the upper gastrointestinal tract, metastatic cancer and acute leukemia is a predictor in the mortality models, while the composite of metastatic cancer and acute leukemia serves as the corresponding predictor in the readmission models

© 2018 Downing NS et al. *JAMA Network Open.*

Appendix Table 6. Patient characteristics among hospitals included in readmission analyses by race.

	Acute myocardial infarction				Heart failure				Pneumonia			
	All	White	Black	p	All	White	Black	p	All	White	Black	p
**Admissions - n (%)**	174719 (100%)	147712 (84.5%)	27007 (15.5%)	n/a	703324 (100%)	564960 (80.3%)	138364 (19.7%)	n/a	378496 (100%)	320593 (84.7%)	57903 (15.3%)	n/a
**Demographics**												
Age (SD)	78.0(8.0)	78.2(7.9)	77.0(8.1)	<0.0001	80.3(8.2)	81.0(8.0)	77.6(8.2)	<0.0001	79.8(8.2)	80.1(8.2)	78.3(8.5)	<0.0001
Male (%)	51.66	53.38	42.22	<0.0001	46.27	47.49	41.27	<0.0001	46.02	46.4	43.89	<0.0001
**Medical history (%)***												
Acute coronary syndrome[†]	23.26	22.59	26.9	<0.0001	17.95	17.74	18.82	<0.0001	8.2	8.02	9.18	<0.0001
Congestive heart failure	33.64	31.88	43.26	<0.0001	77.52	76.65	81.08	<0.0001	39.44	38.6	44.09	<0.0001
Pneumonia	22.6	22.14	25.09	<0.0001	43.92	44.31	42.33	<0.0001	44.11	44.01	44.67	0.0033
Coronary artery bypass grafting	7.68	7.96	6.15	<0.0001	13.06	14.38	7.67	<0.0001	6.26	6.8	3.29	<0.0001
Percutaneous coronary intervention	12.54	12.72	11.55	<0.0001	n/a	n/a	n/a	n/a	n/a	n/a	n/a	n/a
**Comorbidities (%)**												
Diabetes	46.49	44.11	59.47	<0.0001	53.9	51.38	64.19	<0.0001	42.13	39.65	55.83	<0.0001
Valvular and rheumatic heart disease	29.2	29.3	28.7	0.0457	49.9	51.17	44.69	<0.0001	24.55	24.93	22.43	<0.0001
Stroke	8.08	7.35	12.11	<0.0001	10.67	10.14	12.86	<0.0001	10.94	10.07	15.79	<0.0001
Renal failure	25.58	23.08	39.26	<0.0001	48.01	45.46	58.42	<0.0001	28.45	26.25	40.64	<0.0001
Asthma	6.4	5.98	8.69	<0.0001	9.21	8.35	12.71	<0.0001	11.36	11.02	13.23	<0.0001
COPD	29.01	29.36	27.1	<0.0001	46.99	47.54	44.74	<0.0001	53.6	54.92	46.26	<0.0001
Other chronic lung disease	n/a	n/a	n/a	n/a	12.03	12.57	9.83	<0.0001	17.09	17.58	14.34	<0.0001
Metastatic cancer and acute leukemia[†]	2.08	2.03	2.33	0.0019	2.29	2.32	2.17	0.0009	5.73	5.61	6.35	<0.0001
Dementia	17.06	16.02	22.77	<0.0001	22.23	22	23.16	<0.0001	30.47	29.47	36.03	<0.0001
Major psychiatric disorder	n/a	n/a	n/a	n/a	9.63	9.82	8.82	<0.0001	13.02	13.06	12.79	0.0726

* i.e., prior to current admission

[†] Medical history and comorbidity variables reflect defintion used in the models predicting mortality/readmission. While the vast majority of variables are defined similarly in these models, there are some differences and two such differences are shown in these tables: 1) acute myocardial infarction alone is a predictor in the mortality models, while acute coronary syndrome (i.e., the composite of acute myocardial infarction and unstable angina) is the corresponding predictor in the readmission models. 2) the composite of lung cancer, cancer of the upper gastrointestinal tract, metastatic cancer and acute leukemia is a predictor in the mortality models, while the composite of metastatic cancer and acute leukemia serves as the corresponding predictor in the readmission models

© 2018 Downing NS et al. *JAMA Network Open.*

Appendix Table 7. Patient characteristics among hospitals included in mortality analyses by neighborhood income.

	Acute myocardial infarction				Heart failure				Pneumonia			
	All	Higher income	Lower income	P-value	All	Higher income	Lower income	P-value	All	Higher income	Lower income	P-value
**Admissions - n (%)**	161142 (100%)	77410 (48.0%)	83732 (52.0%)	n/a	371102 (100%)	193576 (52.2%)	177526 (47.8%)	n/a	308079 (100%)	169514 (55.0%)	138565 (45.0%)	n/a
**Demographics**												
Age(SD)	78.7(8.1)	79.3(8.2)	78.0(8.0)	<0.0001	80.8(8.1)	81.8(8.0)	79.8(8.2)	<0.0001	80.3(8.2)	80.9(8.1)	79.5(8.3)	<0.0001
Male (%)	51.95	52.57	51.38	<0.0001	46.19	46.91	45.4	<0.0001	46.65	46.53	46.79	0.146
**Medical history (%)***												
Acute myocardial infarction[†]	13.63	13	14.21	<0.0001	9.93	9.62	10.26	<0.00	3.92	3.77	4.1	<0.0001
Congestive heart failure	29.3	27.97	30.53	<0.0001	74.79	73.91	75.74	<0.0001	38.65	37.5	40.06	<0.0001
Pneumonia	21.91	21.52	22.28	0.0002	43.25	43.4	43.09	0.0552	42.37	41.53	43.39	<0.0001
Coronary artery bypass grafting	7.38	7.33	7.42	0.476	12.41	12.63	12.17	<0.0001	6.27	6.42	6.1	0.0002
Percutaneous coronary intervention	11.21	10.9	11.5	0.0001	8.76	8.69	8.84	0.1129	4.82	4.84	4.79	0.5675
**Comorbidities (%)***												
Diabetes	44.13	41.49	46.57	<0.0001	52.43	49.09	56.07	<0.0001	n/a	n/a	n/a	n/a
Valvular and rheumatic heart disease	28.02	28.28	27.78	0.0248	49.48	51.21	47.6	<0.0001	n/a	n/a	n/a	n/a
Stroke	7.53	7.52	7.54	0.844	10.26	10.27	10.25	0.8557	10.39	10.25	10.56	0.0046
Renal failure	23.2	22.08	24.24	<0.0001	46.48	44.95	48.16	<0.0001	28.31	26.87	30.08	<0.0001
Asthma	n/a	n/a	n/a	n/a	n/a	n/a	n/a	n/a	11.31	11.21	11.44	0.0405
COPD	27.46	25.03	29.71	<0.0001	45.26	43.14	47.57	<0.0001	52.72	50.35	55.63	<0.0001
Other chronic lung disease	n/a	n/a	n/a	n/a	n/a	n/a	n/a	n/a	16.91	16.98	16.82	0.248
Lung cancer, metastatic cancer and acute leukemia[†]	3.83	3.95	3.73	0.0228	4.57	4.82	4.3	<0.0001	10.43	10.39	10.47	0.4818
Dementia	17.27	18.08	16.52	<0.0001	22.23	23.12	21.25	<0.0001	29.6	30.14	28.94	<0.0001
Major psychiatric disorder	6.74	6.77	6.71	0.6618	9.64	9.85	9.41	<0.0001	12.97	12.94	13.01	0.5504

* i.e., prior to current admission

† Medical history and comorbidity variables reflect defintion used in the models predicting mortality/readmission. While the vast majority of variables are defined similarly in these models, there are some differences and two such differences are shown in these tables: 1) acute myocardial infarction alone is a predictor in the mortality models, while acute coronary syndrome (i.e., the composite of acute myocardial infarction and unstable angina) is the corresponding predictor in the readmission models. 2) the composite of lung cancer, cancer of the upper gastrointestinal tract, metastatic cancer and acute leukemia is a predictor in the mortality models, while the composite of metastatic cancer and acute leukemia serves as the corresponding predictor in the readmission models

© 2018 Downing NS et al. *JAMA Network Open.*

Appendix Table 8. Patient characteristics among hospitals included in readmission analyses by neighborhood income.

	Acute myocardial infarction				Heart failure				Pneumonia			
	All	Higher income	Lower income	P-value	All	Higher income	Lower income	P-value	All	Higher income	Lower income	P-value
**Admissions - n (%)**	192435 (100%)	92275 (48.0%)	100160 (52.0%)	n/a	494811 (100%)	258610 (52.3%)	236201 (47.7%)	n/a	343284 (100%)	188029 (54.8%)	155255 (45.2%)	n/a
**Demographics**												
Age(SD)	78.1(8.0)	78.8(8.0)	77.6(7.9)	<0.0001	80.5(8.1)	81.5(8.0)	79.4(8.2)	<0.0001	80.1(8.2)	80.7(8.1)	79.3(8.2)	<0.0001
Male (%)	52.57	53.39	51.81	<0.0001	46.81	47.59	45.96	<0.0001	46.98	46.86	47.13	0.1203
**Medical history (%)***												
Acute coronary syndrome[†]	22.75	21.62	23.79	<0.0001	18.18	17.17	19.29	<0.0001	8.02	7.52	8.62	<0.0001
Congestive heart failure	31.86	30.42	33.19	<0.0001	77.5	76.5	78.6	<0.0001	38.86	37.49	40.53	<0.0001
Pneumonia	21.7	21.01	22.33	<0.0001	43.7	43.53	43.89	0.0099	43.7	42.84	44.73	<0.0001
Coronary artery bypass grafting	7.57	7.52	7.61	0.4558	13.21	13,51	12.89	<0.0001	6.42	6.53	6.28	0.0034
Percutaneous coronary intervention	12.59	12.32	12.83	0.0007	n/a	n/a	n/a	n/a	n/a	n/a	n/a	n/a
**Comorbidities (%)***												
Diabetes	45.3	42.69	47.71	<0.0001	53.55	50.2	57.22	<0.0001	41.51	38.49	45.16	<0.0001
Valvular and rheumatic heart disease	28.73	29.38	28.13	<0.0001	50.85	52.62	48.93	<0.0001	24.84	25.46	24.08	<0.0001
Stroke	7.32	7.37	7.27	0.3576	10.39	10.38	10.39	0.9219	10.26	10.08	10.48	0.0001
Renal failure	24.4	23.37	25.35	<0.0001	48.37	46.57	50.35	<0.0001	28.37	26.89	30.16	<0.0001
Asthma	6.29	6.24	6.34	0.3984	9.23	8.68	9.84	<0.0001	11.62	11.49	11.77	0.0105
COPD	28.57	25.66	31.25	<0.0001	46.47	44.1	49.07	<0.0001	53.52	50.95	56.63	<0.0001
Other chronic lung disease	n/a	n/a	n/a	n/a	11.93	12.23	11.61	<0.0001	17.4	17.36	17.46	0.4562
Metastatic cancer and acute leukemia[†]	1.93	2.02	1.86	0.011	2.31	2.47	2.14	<0.0001	5.54	5.6	5.47	0.1092
Dementia	16.1	16.69	15.55	<0.0001	21.72	22.47	20.91	<0.0001	29.32	29.88	28.63	<0.0001
Major psychiatric disorder	n/a	n/a	n/a	n/a	9.63	9.79	9.45	<0.0001	13.02	12.97	13.08	0.3313

* i.e., prior to current admission

[†] Medical history and comorbidity variables reflect defintion used in the models predicting mortality/readmission. While the vast majority of variables are defined similarly in these models, there are some differences and two such differences are shown in these tables: 1) acute myocardial infarction alone is a predictor in the mortality models, while acute coronary syndrome (i.e., the composite of acute myocardial infarction and unstable angina) is the corresponding predictor in the readmission models. 2) the composite of lung cancer, cancer of the upper gastrointestinal tract, metastatic cancer and acute leukemia is a predictor in the mortality models, while the composite of metastatic cancer and acute leukemia serves as the corresponding predictor in the readmission models

© 2018 Downing NS et al. *JAMA Network Open.*

Appendix Table 9: Within-hospital differences in risk-standardized mortality and readmission rates in a sensitivity analysis in which any hospital with at least 10 patients in each race or neighborhood income subgroup was included.

Race				
	Main analysis (≥ 25 patients in each subgroup)		Sensitivity analysis (≥ 10 patients in each subgroup)	
	Mean (SD) within-hospital difference (rate among black patients less that among white patients)	*P value**	*Mean (SD) within-hospital difference (rate among black patients less that among white patients)*	*P value**
Mortality				
AMI**	-0.6% (1.1%)	0.47	-0.7% (1.1%)	0.33
Heart Failure	-4.7% (1.3%)	<0.001	-4.7% (1.2%)	<0.001
Pneumonia	-1.0% (2.0%)	0.05	-0.9% (1.8%)	0.05
Readmission				
AMI**	4.3% (1.4%)	<0.001	4.4% (1.2%)	<0.001
Heart Failure	2.8% (1.8%)	<0.001	2.8% (1.7%)	<0.001
Pneumonia	3.7% (1.3%)	<0.001	3.7% (1.2%)	<0.001
Neighborhood income				
	Main analysis (≥ 25 patients in each subgroup)		Sensitivity analysis (≥ 10 patients in each subgroup)	
	Mean (SD) within-hospital difference (rate among patients from lower-income neighborhood less that among patients from higher-income neighborhood)	*P value**	*Mean (SD) within-hospital difference (ratio among patients from lower-income neighborhood less that among patients from higher-income neighborhood)*	*P value**
Mortality				
AMI**	0.1% (0.9%)	0.83	-0.1% (0.9%)	0.95
Heart Failure	-1.1% (1.3%)	0.04	-1.0% (1.3%)	0.06
Pneumonia	0.1% (1.5%)	0.41	0.2% (1.4%)	0.37
Readmission				
AMI**	0.7% (1.1%)	0.41	0.7% (1.0%)	0.45
Heart Failure	1.1% (1.8%)	0.26	1.2% (1.7%)	0.20
Pneumonia	1.2% (1.4%)	0.10	1.1% (1.2%)	0.14

*****P values calculated using t-tests weighted by the proportion of patients in each subgroup treated at each hospital**.**
**AMI, acute myocardial infarction

© 2018 Downing NS et al. *JAMA Network Open.*

Appendix Table 10: Within-hospital differences in risk-standardized mortality and readmission ratios in a sensitivity analysis in which any hospital with at least 10 patients in each race or neighborhood income subgroup was included.

Race						
	Main analysis (≥ 25 patients in each subgroup)			Sensitivity analysis (≥ 10 patients in each subgroup)		
	Mean (SD) within-hospital difference (ratio among black patients less that among white patients)	*P value**	*Intra-class correlation coefficient (95% confidence interval)*	*Mean (SD) within-hospital difference (ratio among black patients less that among white patients)*	*P value**	*Intra-class correlation coefficient (95% confidence interval)*
Mortality						
AMI**	-9.1×10^{-4} (0.076)	0.91	0.68 (0.64-0.72)	-1.7×10^{-3} (0.079)	0.84	0.78 (0.75-0.80)
Heart Failure	-2.1×10^{-3} (0.12)	0.89	0.72 (0.69-0.75)	-2.3×10^{-3} (0.11)	0.95	0.76 (0.74-0.78)
Pneumonia	-2.4×10^{-3} (0.16)	0.50	0.70 (0.67-0.73)	-5.7×10^{-3} (0.15)	0.64	0.79 (0.77-0.81)
Readmission						
AMI**	-6.3×10^{-4} (0.069)	0.83	0.73 (0.69-0.77)	-9.0×10^{-4} (0.065)	0.86	0.80 (0.77-0.82)
Heart failure	-6.2×10^{-4} (0.073)	0.73	0.73 (0.71-0.75)	-8.5×10^{-4} (0.072)	0.70	0.77 (0.75-0.79)
Pneumonia	-1.3×10^{-3} (0.068)	0.87	0.79 (0.76-0.82)	-1.3×10^{-3} (0.066)	0.87	0.83 (0.81-0.85)
Neighborhood income						
	Main analysis (≥ 25 patients in each subgroup)			Sensitivity analysis (≥ 10 patients in each subgroup)		
	Mean (SD) within-hospital difference (ratio among patients from lower-income neighborhood less that among patients from higher-income neighborhood)	*P value**	*Intra-class correlation coefficient (95% confidence interval)*	*Mean (SD) within-hospital difference (ratio among patients from lower-income neighborhood less that among patients from higher-income neighborhood)*	*P value**	*Intra-class correlation coefficient (95% confidence interval)*
Mortality						
AMI**	-6.7×10^{-4} (0.062)	0.91	0.46 (0.42-0.50)	-3.3×10^{-4} (0.061)	0.88	0.57 (0.53-0.60)
Heart Failure	3.3×10^{-3} (0.12)	0.75	0.59 (0.56-0.62)	3.2×10^{-3} (0.12)	0.71	0.64 (0.61-0.67)
Pneumonia	-1.8×10^{-3} (0.13)	0.66	0.57 (0.54-0.60)	-1.1×10^{-3} (0.12)	0.68	0.65 (0.62-0.67)
Readmission						
AMI**	-3.2×10^{-4} (0.060)	0.97	0.60 (0.57-0.63)	-1.9×10^{-4} (0.055)	0.96	0.67 (0.63-0.70)
Heart Failure	2.0×10^{-4} (0.075)	0.96	0.60 (0.57-0.63)	2.5×10^{-4} (0.074)	0.98	0.66 (0.64-0.69)
Pneumonia	2.4×10^{-4} (0.073)	0.92	0.57 (0.54-0.60)	-3.7×10^{-5} (0.067)	0.93	0.66 (0.63-0.68)

***P values calculated using t-tests weighted by the proportion of patients in each subgroup treated at each hospital.**
**AMI, acute myocardial infarction

© 2018 Downing NS et al. *JAMA Network Open.*

APPENDIX FIGURE 1. Approach to identification of mortality and readmission cohorts for each of the 3 conditions of interest: acute myocardial infarction, heart failure, and pneumonia.

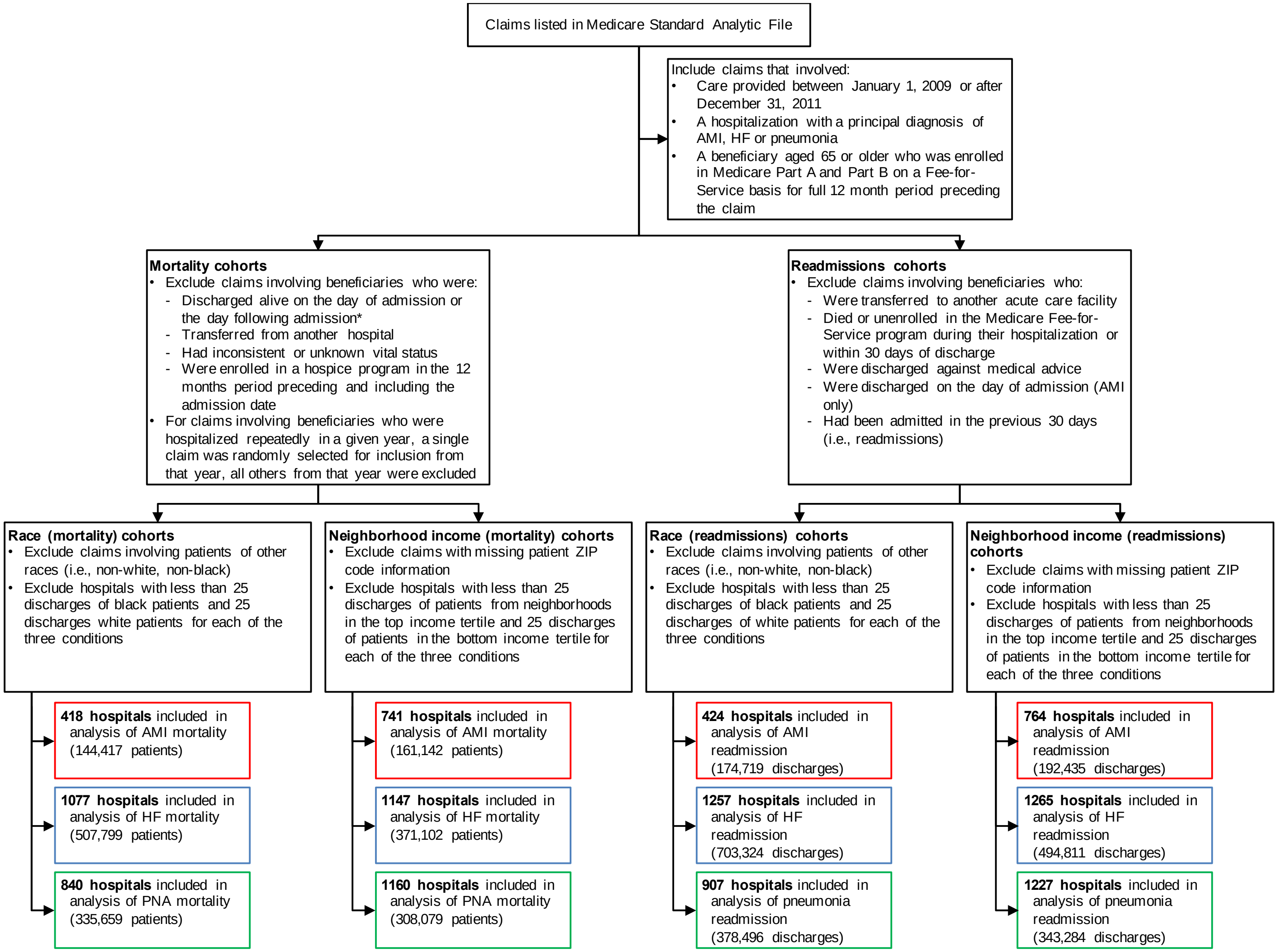

© 2018 Downing NS et al. *JAMA Network Open.*

APPENDIX FIGURE 2. Scatterplots showing between-hospital variation in risk-standardized mortality rates (RSMRs) and risk-standardized readmission rates (RSRRs) according to race. Hospital RSMRs and RSRRs for acute myocardial infarction (AMI), heart failure, and pneumonia among all patients and among black patients are plotted against the proportion of black patients treated at each hospital for these conditions.

Panel A: Risk-standardized mortality rates

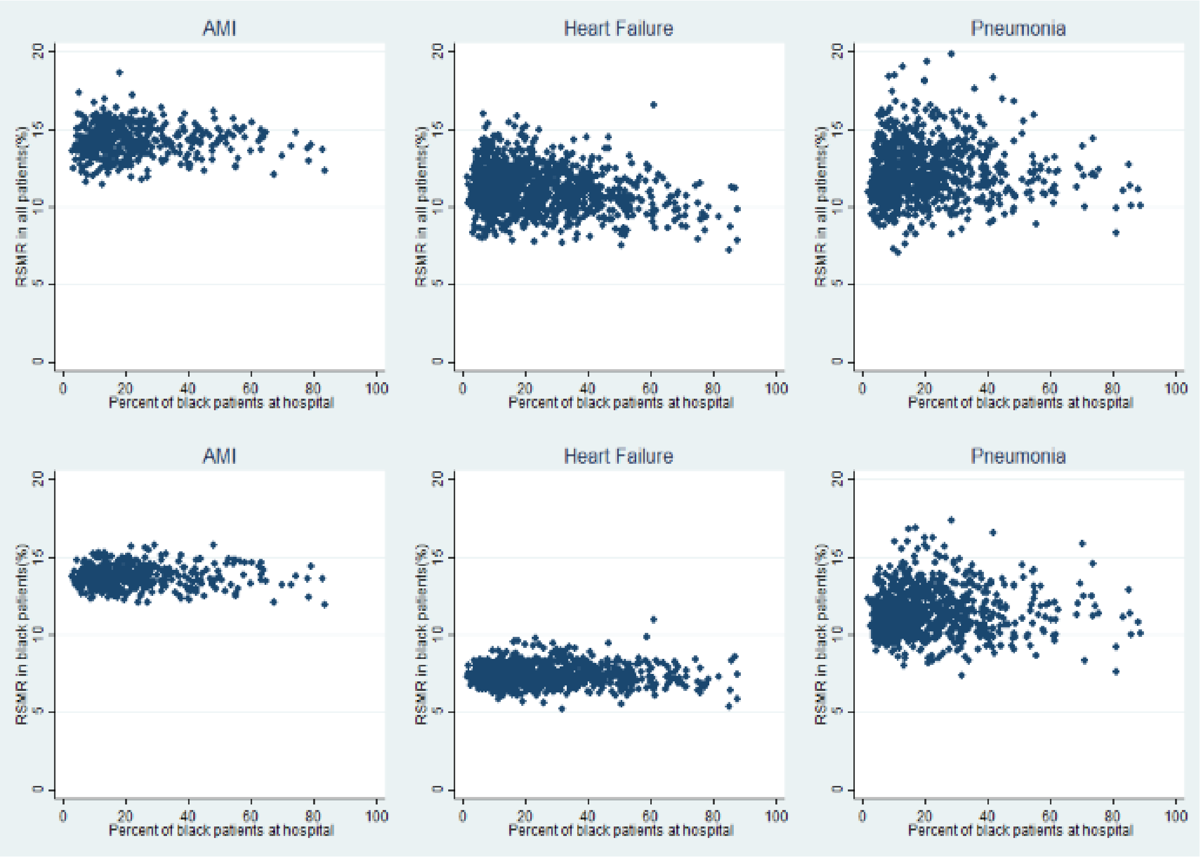

© 2018 Downing NS et al. *JAMA Network Open.*

Panel B: Risk-standardized readmission rates

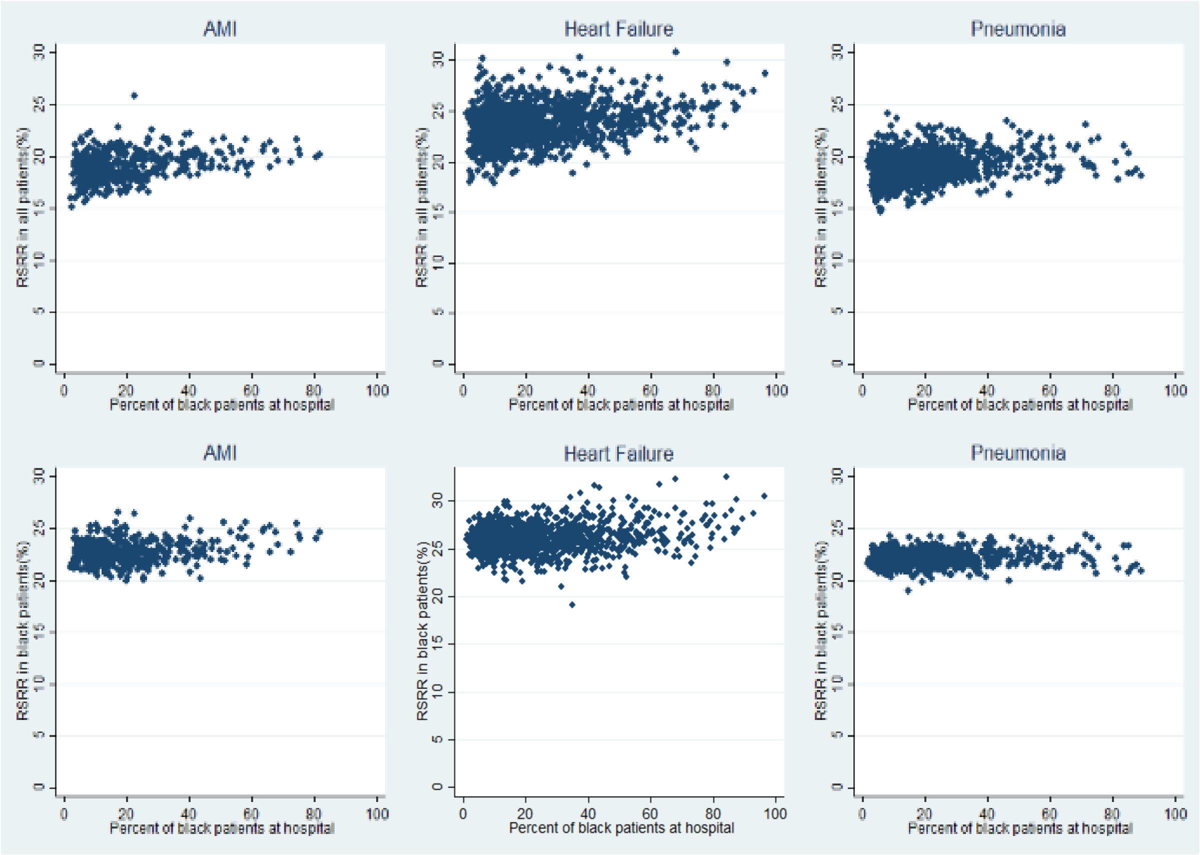

© 2018 Downing NS et al. *JAMA Network Open.*

APPENDIX FIGURE 3. Risk-standardized mortality rates (RSMRs) for all patients, white patients and black patients treated for acute myocardial infarction, heart failure and pneumonia by decile of the proportion of black patients treated at each hospital.

Panel A: Acute myocardial infarction – all patients

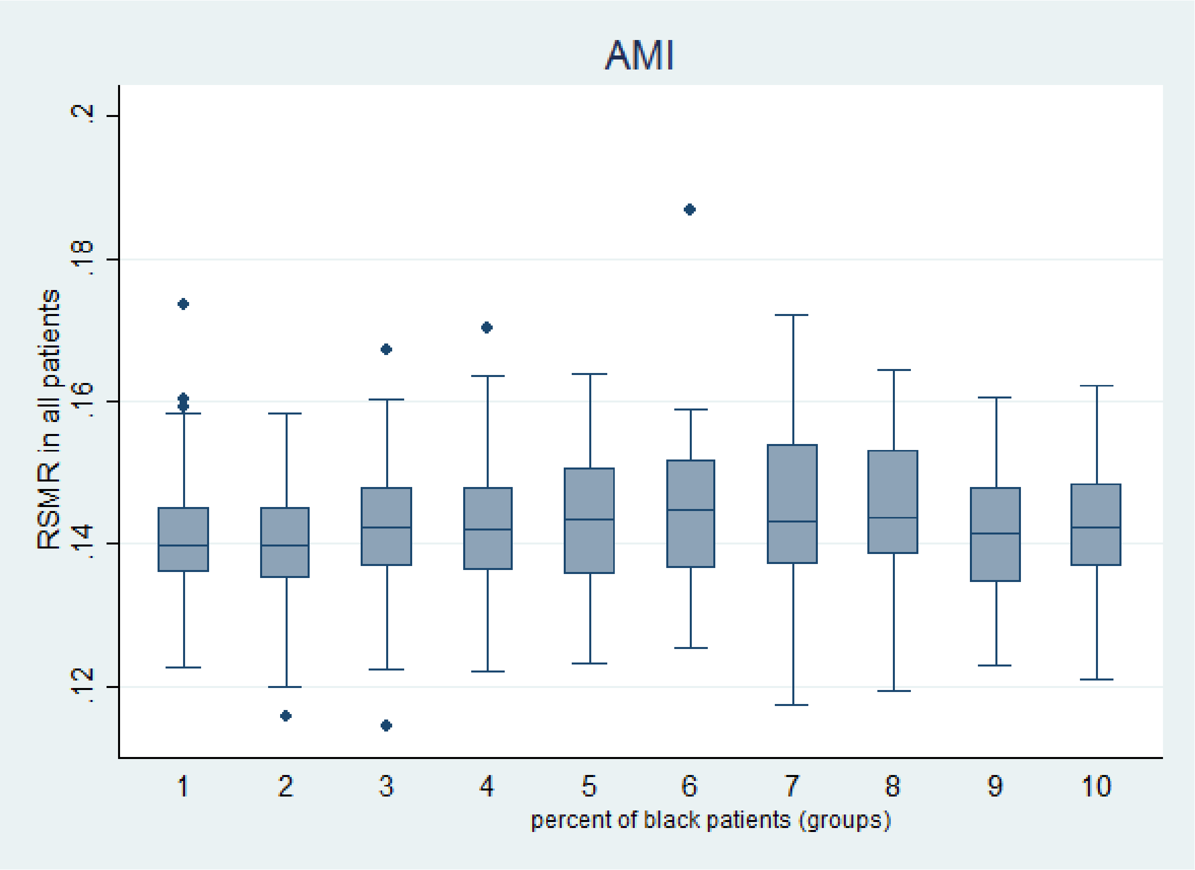

Kruskal-Wallis test P-value: 0.12
Pearson correlation coefficient: 0.04 (P=0.48)

© 2018 Downing NS et al. *JAMA Network Open.*

Panel B: Acute myocardial infarction – white patients

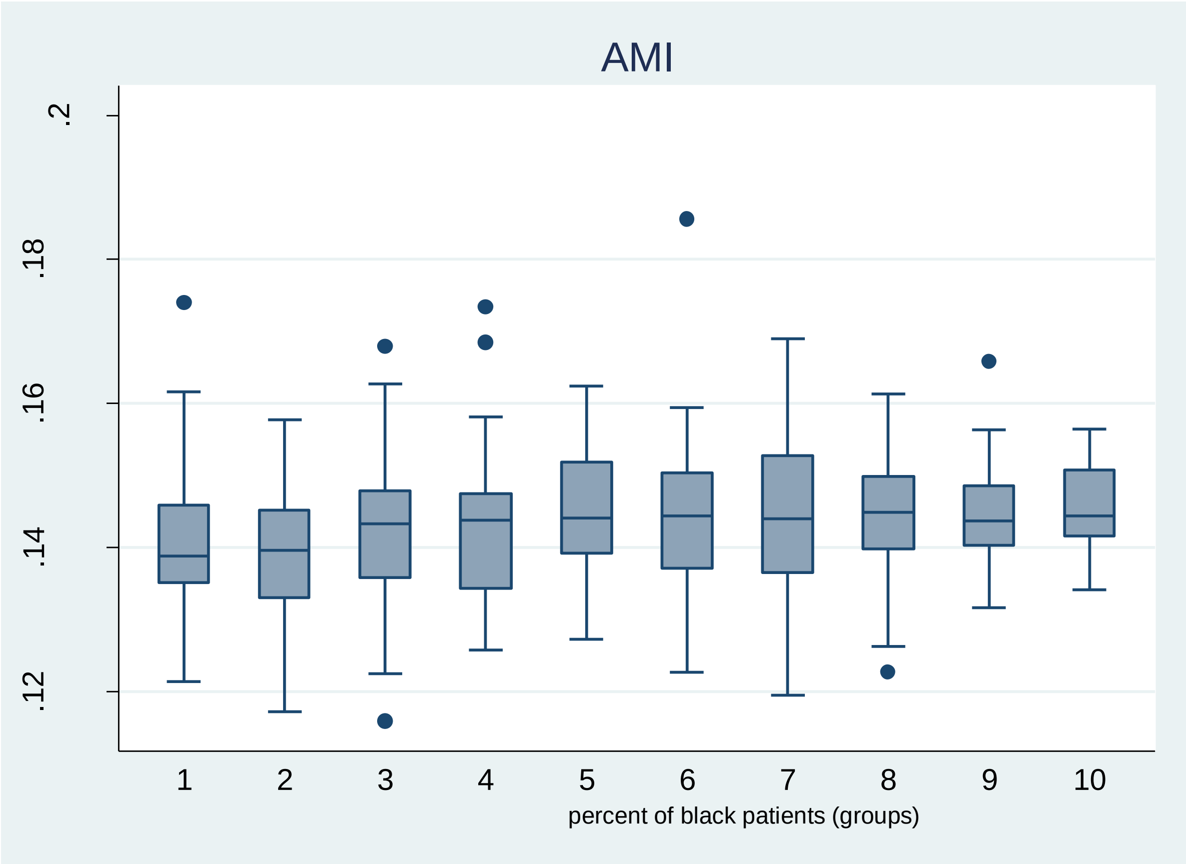

Kruskal-Wallis test P-value: 0.07
Pearson correlation coefficient: 0.12 (P=0.01)

© 2018 Downing NS et al. *JAMA Network Open.*

Panel C: Acute myocardial infarction – black patients

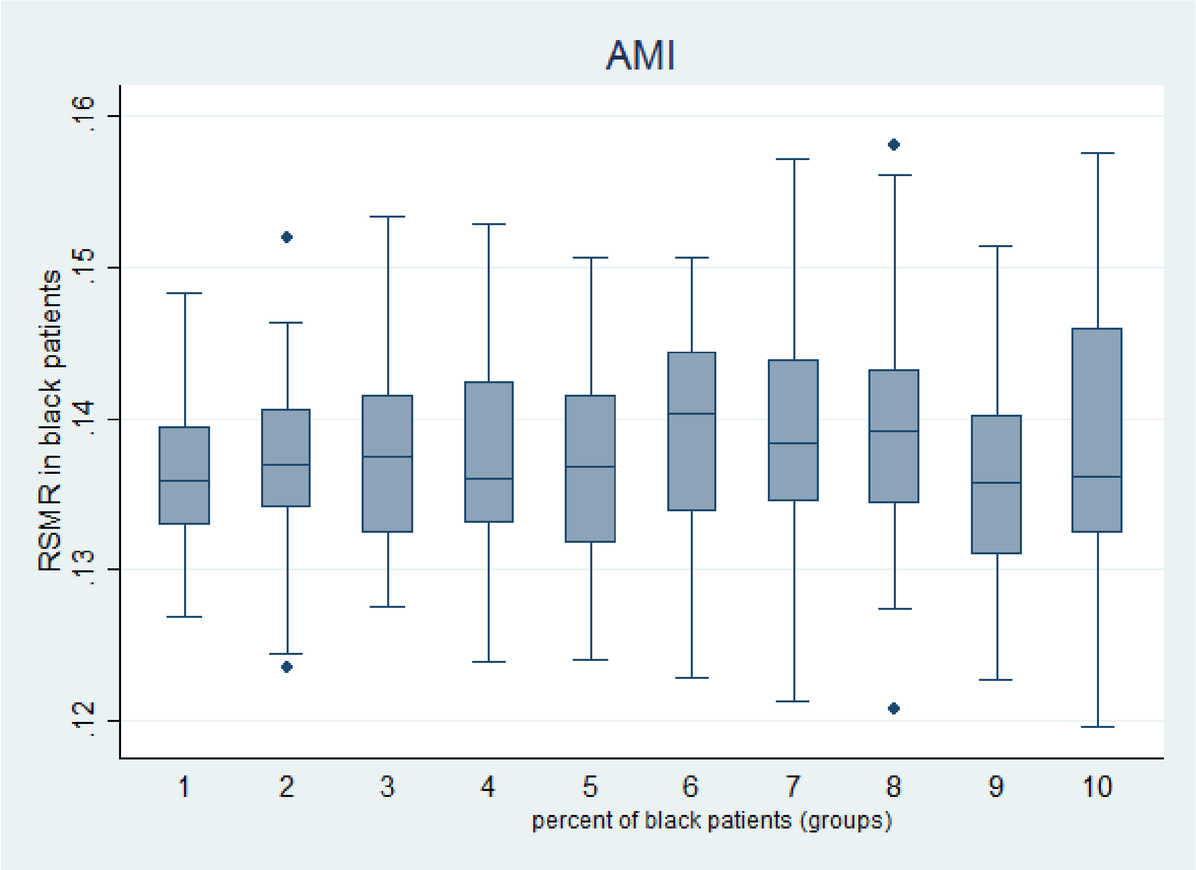

Kruskal-Wallis test P-value: 0.40
Pearson correlation coefficient: -0.01 (P=0.86)

© 2018 Downing NS et al. *JAMA Network Open.*

Panel D: Heart failure – all patients

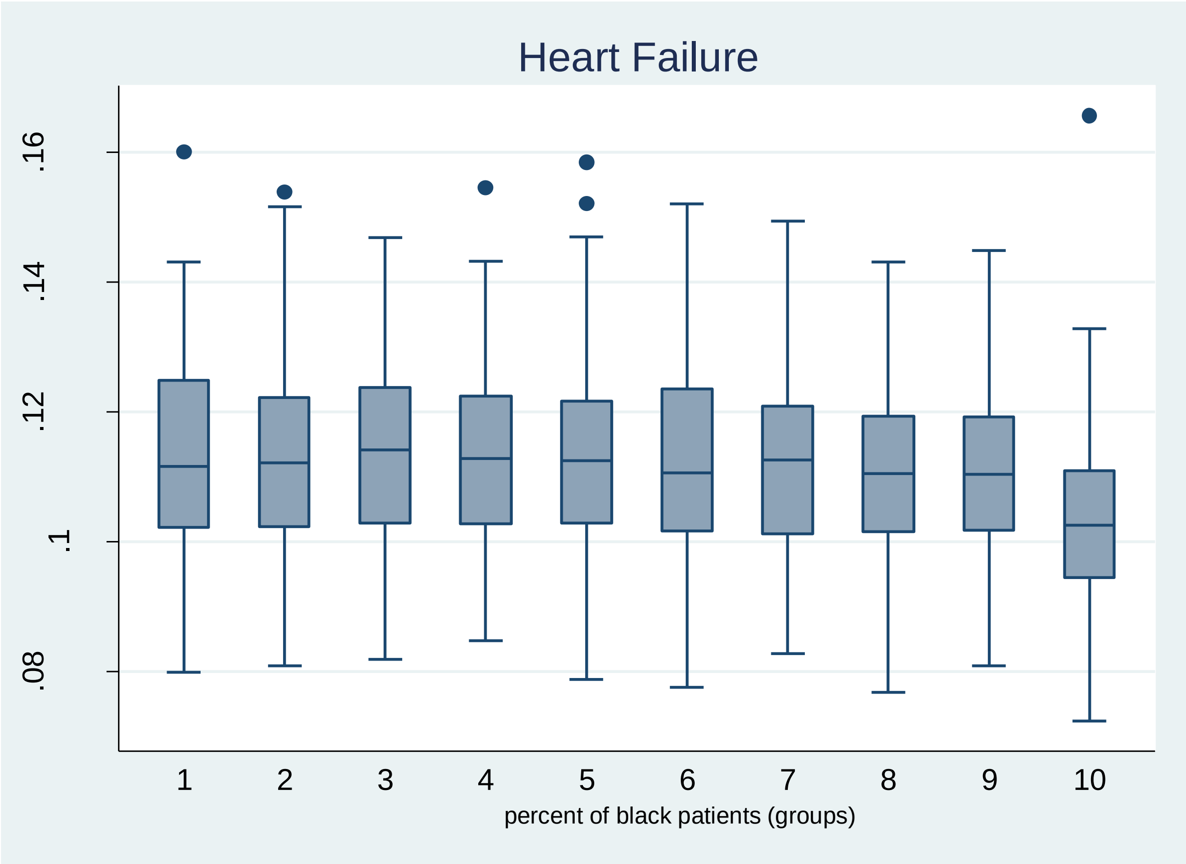

Kruskal-Wallis Test P-value: <0.001
Pearson correlation coefficient: -0.17 (P<0.001)

© 2018 Downing NS et al. *JAMA Network Open.*

Panel E: Heart failure – white patients

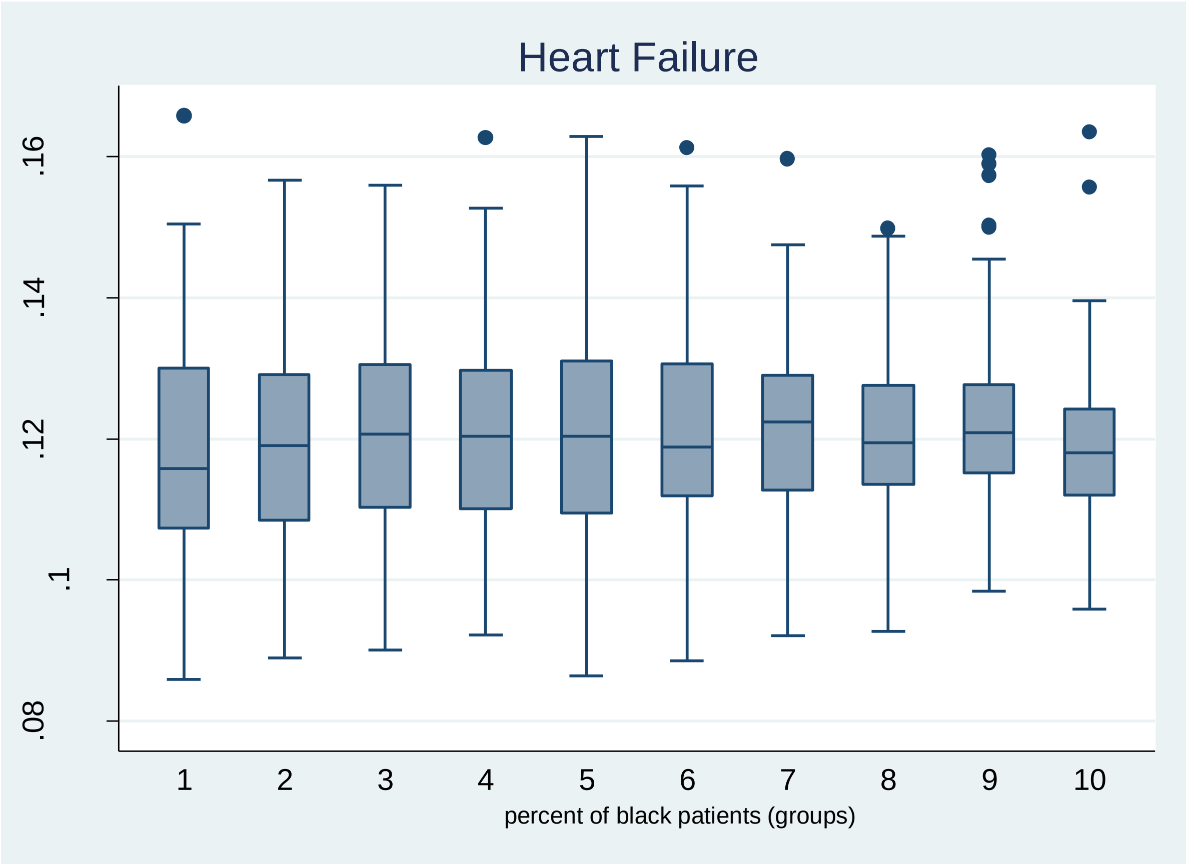

Kruskal-Wallis test P-value: 0.61
Pearson correlation coefficient: 0.01 (P=0.72)

© 2018 Downing NS et al. *JAMA Network Open.*

Panel F: Heart failure – black patients

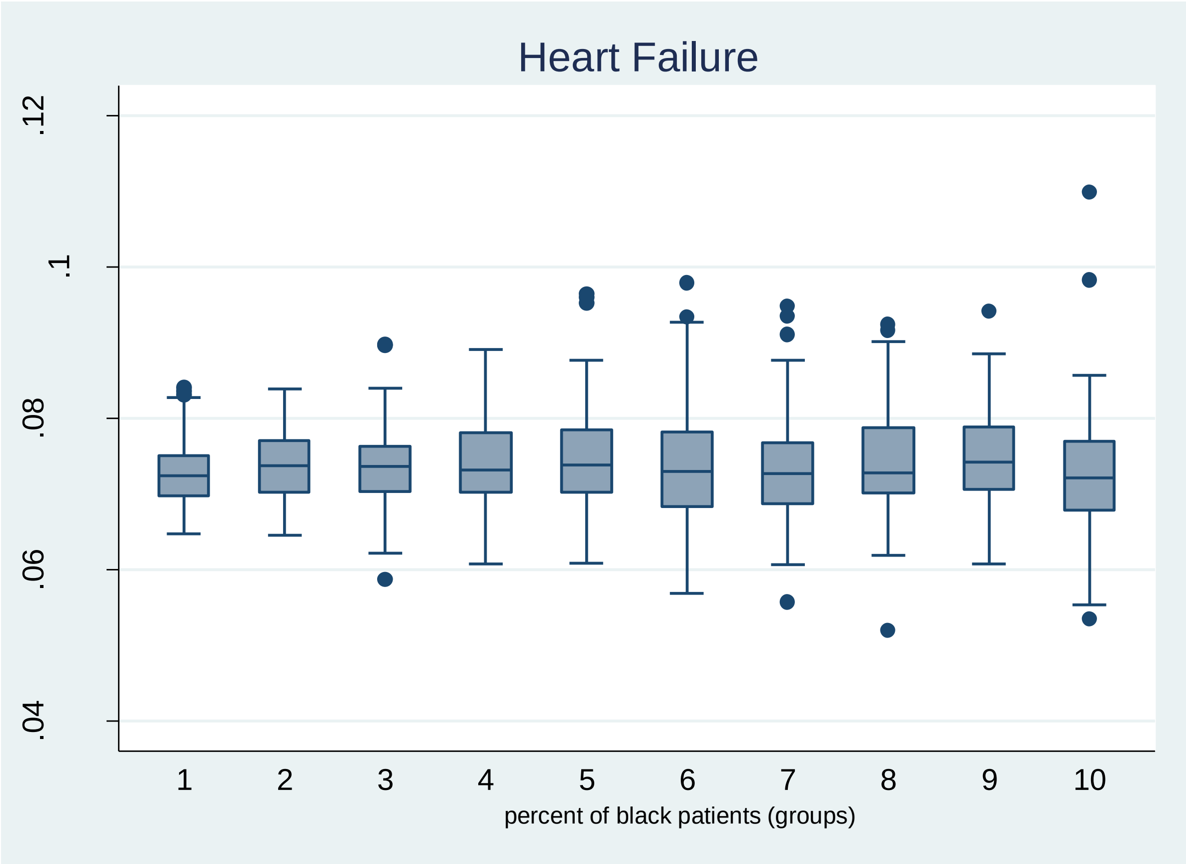

Kruskal-Wallis test P-value: 0.19
Pearson correlation coefficient: -0.02 (P=0.56)

© 2018 Downing NS et al. *JAMA Network Open.*

Panel G: Pneumonia – all patients

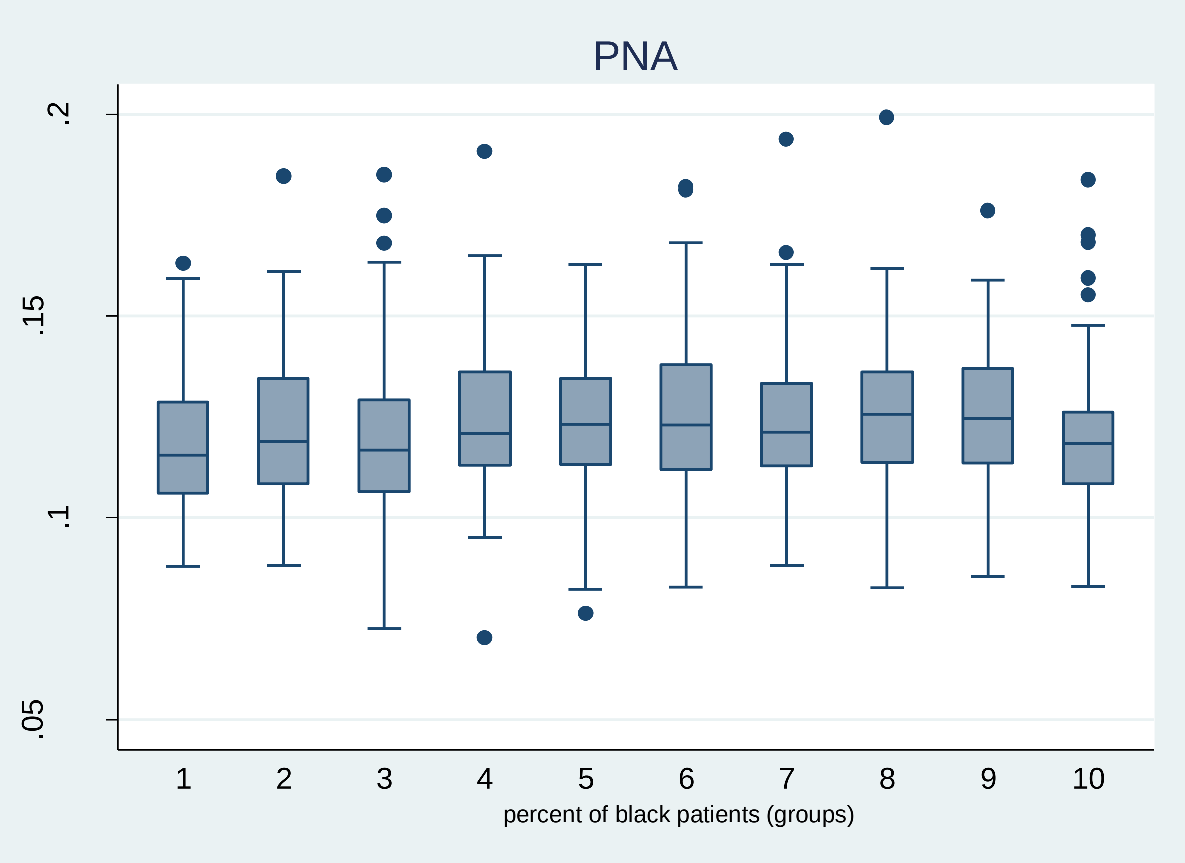

Kruskal-Wallis test P-value: 0.004
Pearson correlation coefficient: 0.002 (P=0.96)

© 2018 Downing NS et al. *JAMA Network Open.*

Panel H: Pneumonia – white patients

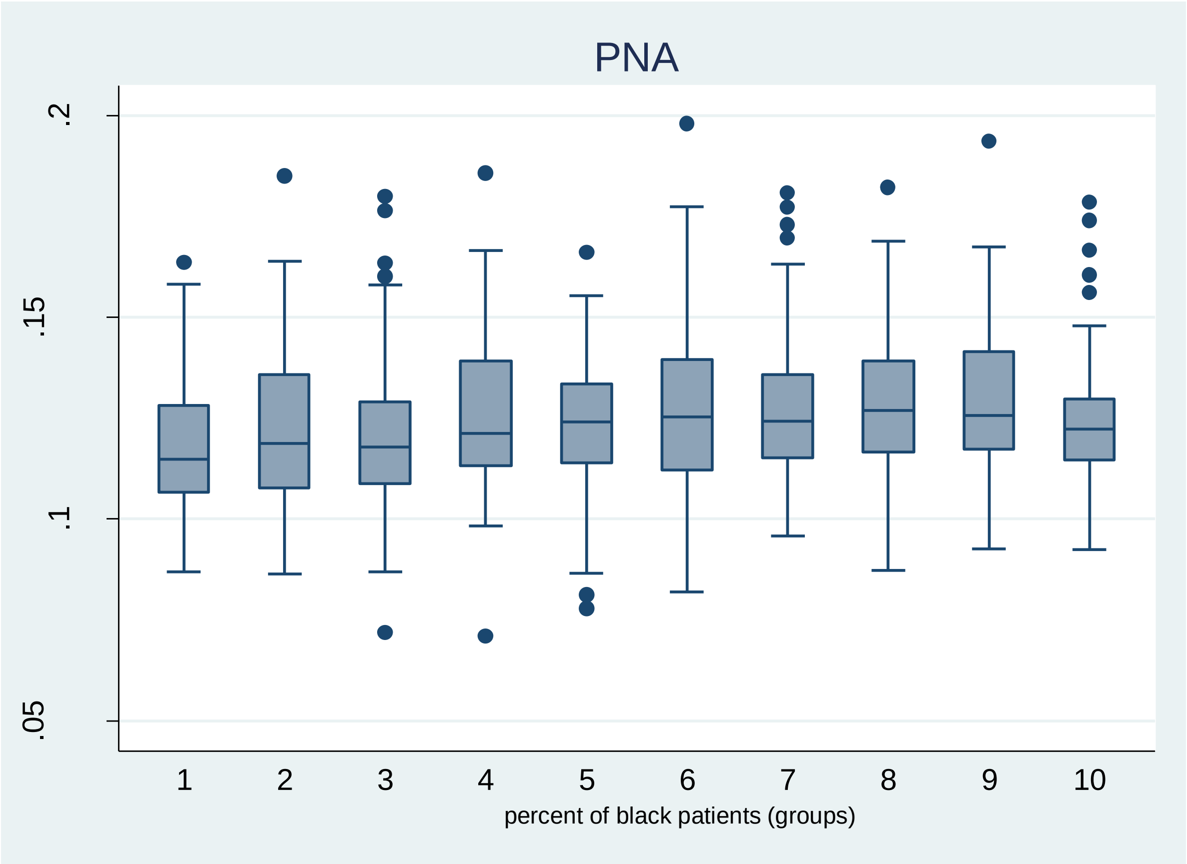

Kruskal-Wallis test P-value: <0.001
Pearson correlation coefficient: 0.09 (P=0.01)

© 2018 Downing NS et al. *JAMA Network Open.*

Panel I: Pneumonia – black patients

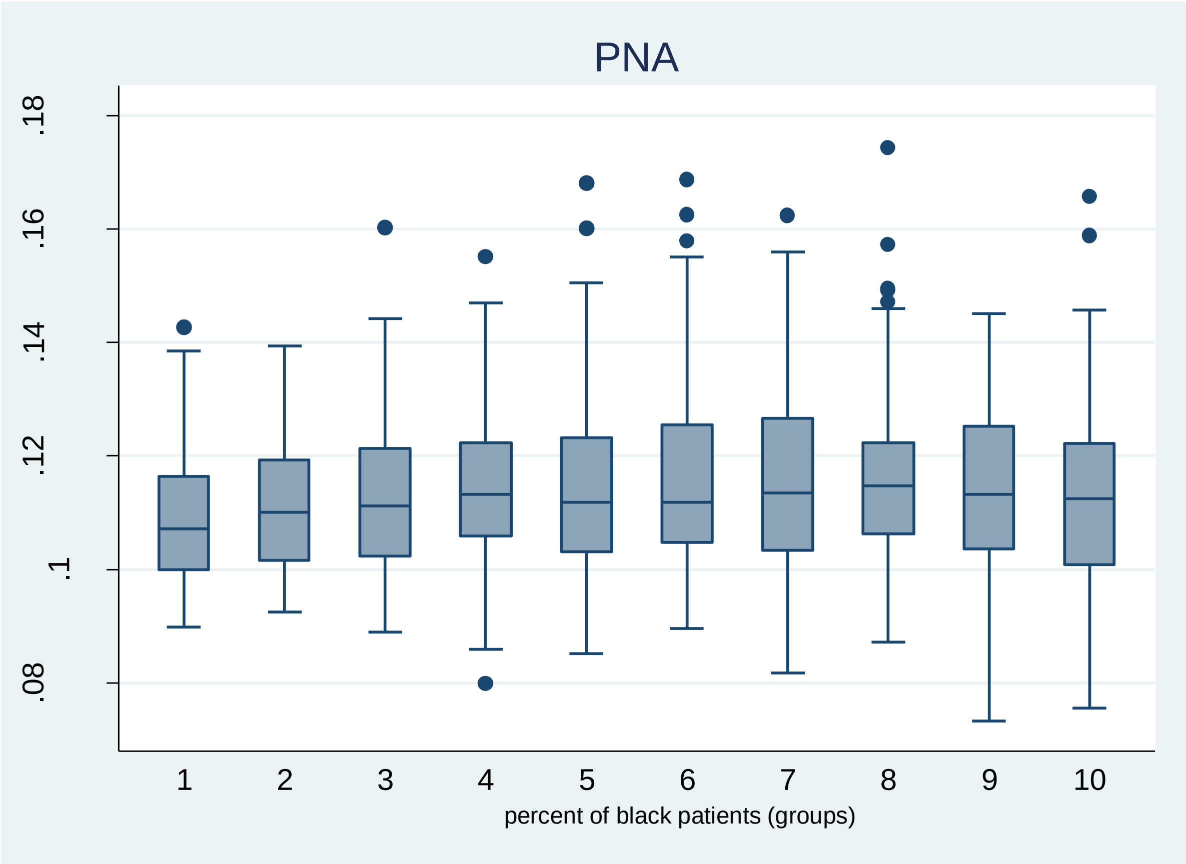

Kruskal-Wallis test P-value: 0.06
Pearson correlation coefficient: 0.04 (P=0.26)

© 2018 Downing NS et al. *JAMA Network Open.*

APPENDIX FIGURE 4. Risk-standardized readmission rates (RSRRs) for all patients, white patients and black patients treated for acute myocardial infarction, heart failure and pneumonia by decile of the proportion of black patients treated at each hospital.

Panel A: Acute myocardial infarction – all patients

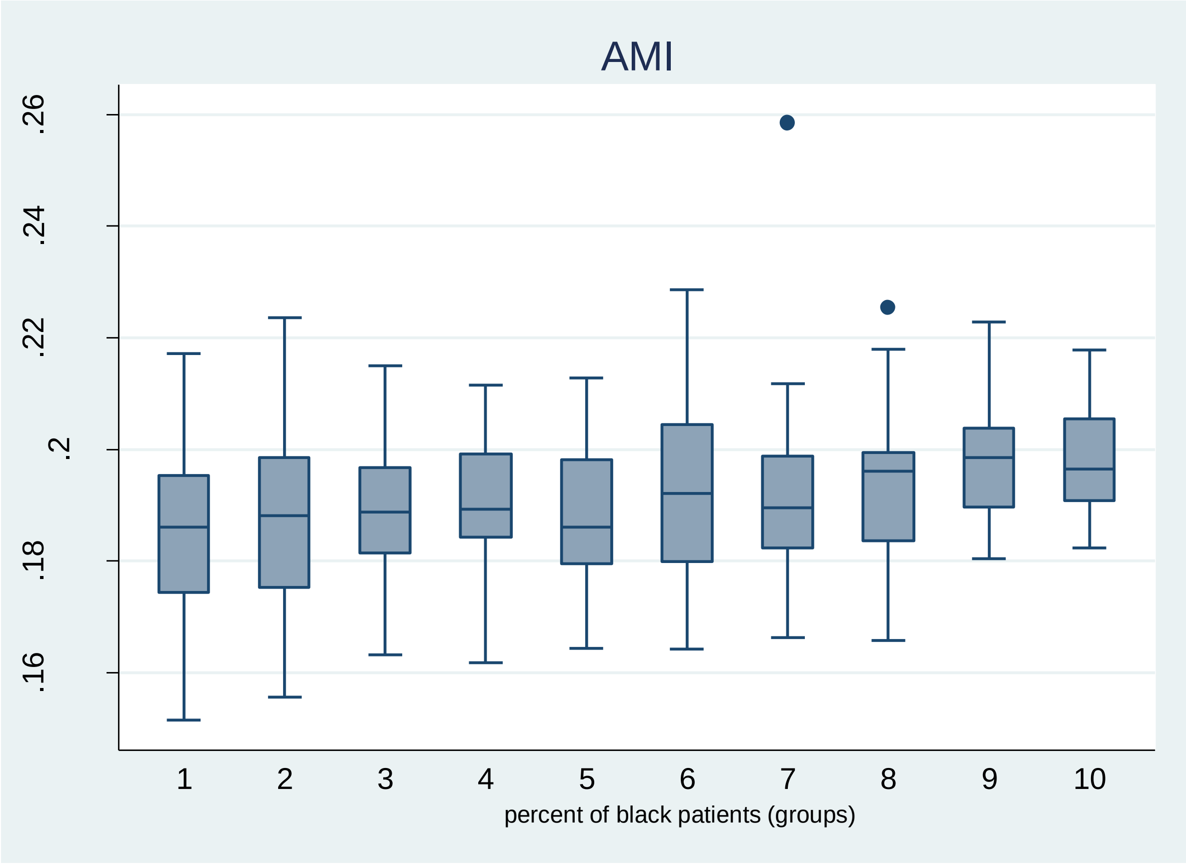

Kruskal-Wallis test P-value: <0.001
Pearson correlation coefficient: 0.28 (P<0.001)

© 2018 Downing NS et al. *JAMA Network Open.*

Panel B: Acute myocardial infarction – white patients

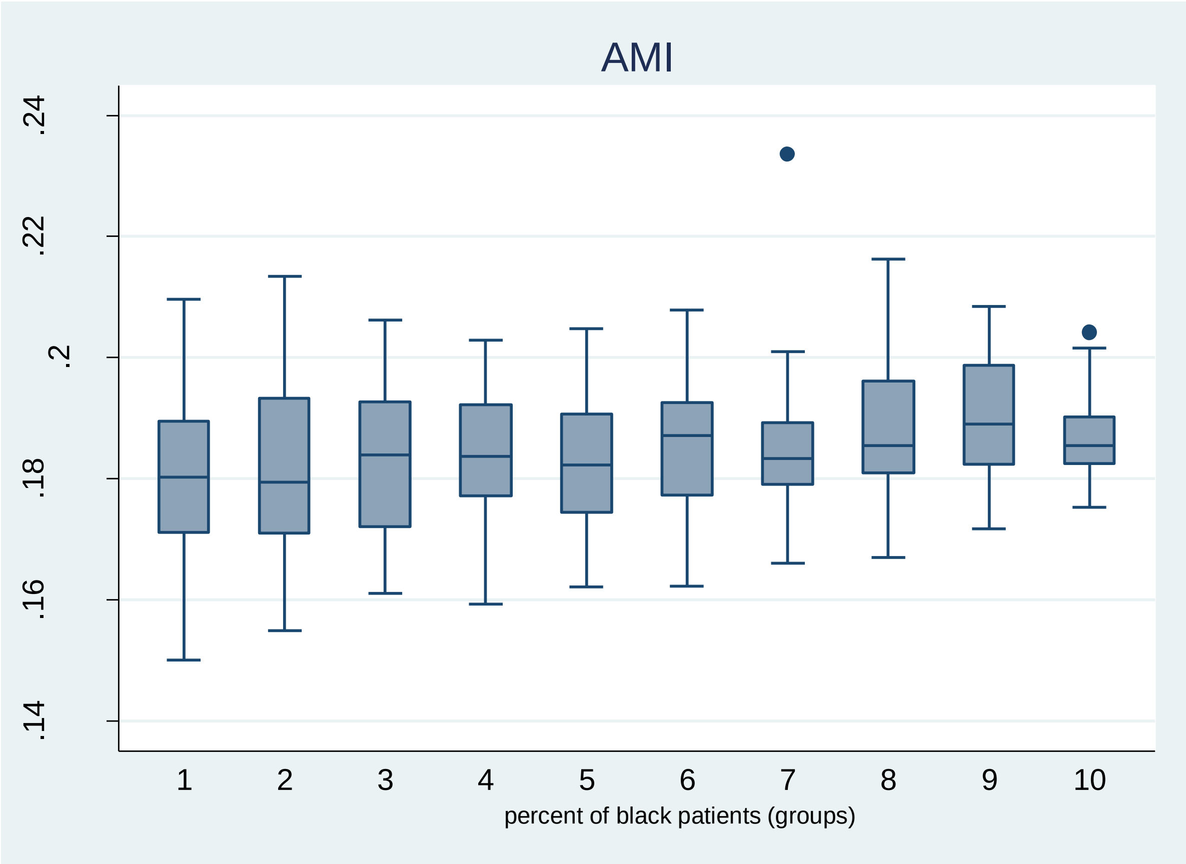

Kruskal-Wallis test P-value: 0.005
Pearson correlation coefficient: 0.18 (P=0.002)

© 2018 Downing NS et al. *JAMA Network Open.*

Panel C: Acute myocardial infarction – black patients

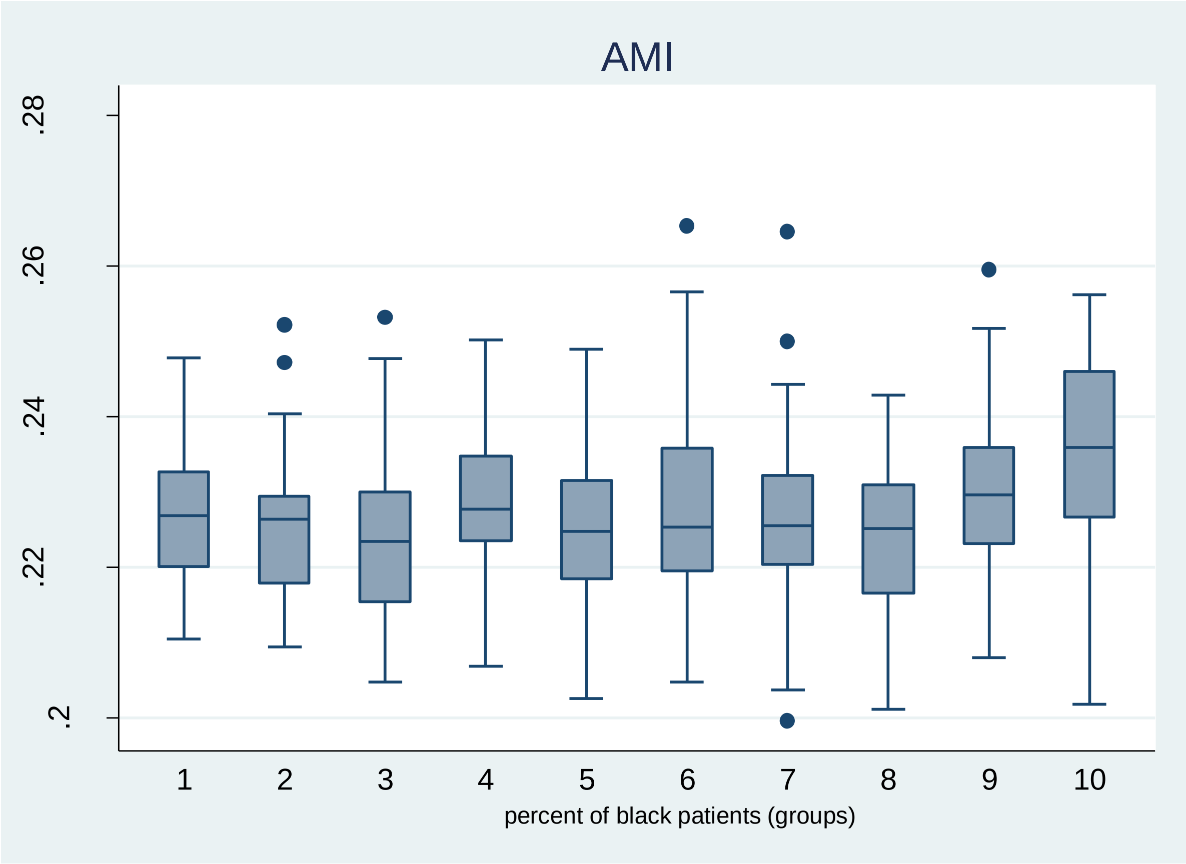

Kruskal-Wallis test P-value: 0.001
Pearson correlation coefficient: 0.25 (P<0.001)

© 2018 Downing NS et al. *JAMA Network Open.*

Panel D: Heart failure – all patients

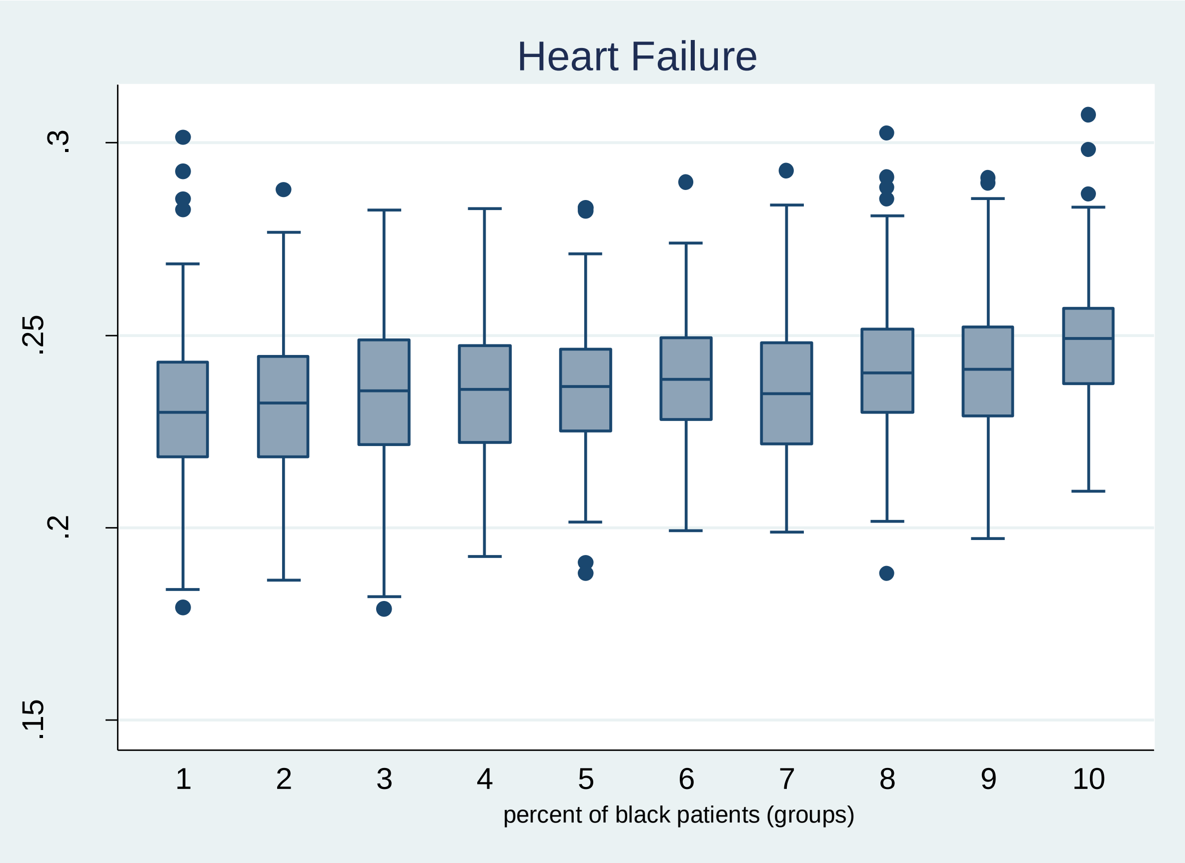

Kruskal-Wallis test P-value: <0.001
Pearson correlation coefficient: 0.26 (P<0.001)

© 2018 Downing NS et al. *JAMA Network Open.*

Panel E: Heart failure – white patients

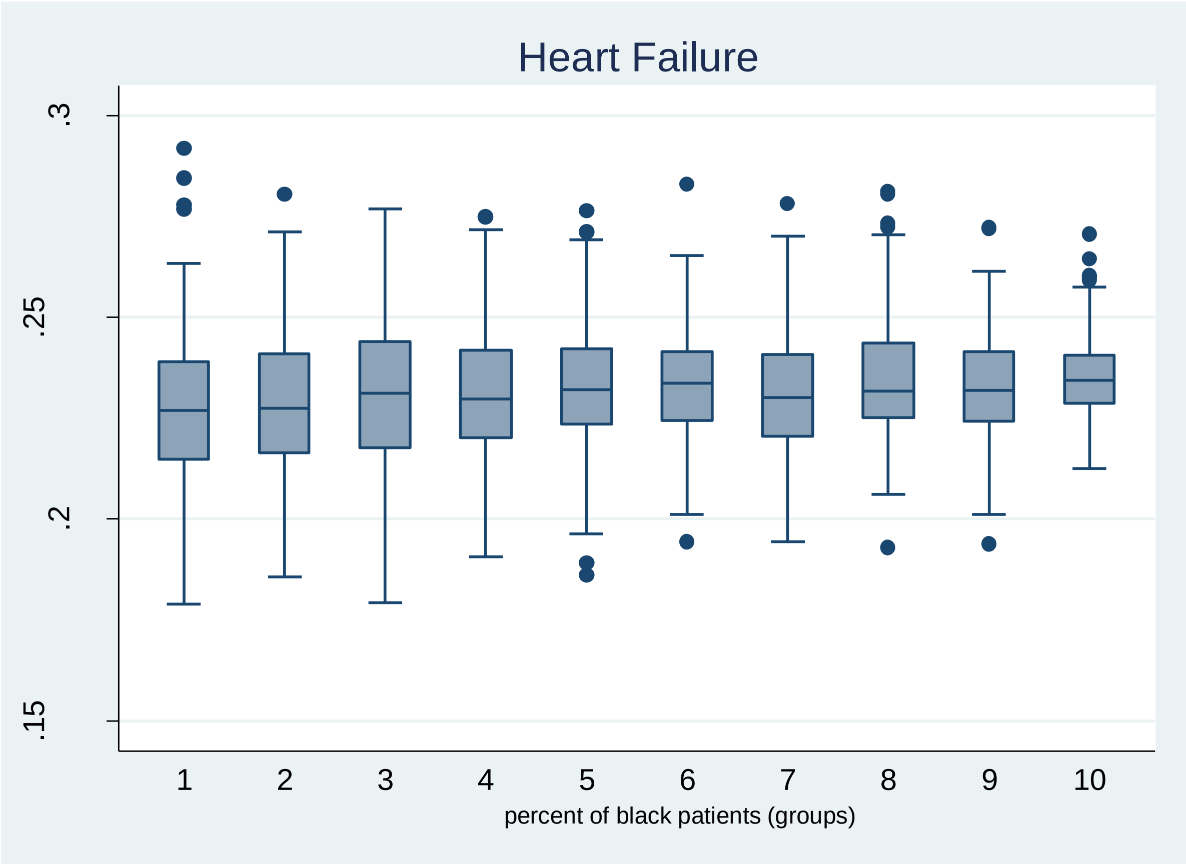

Kruskal-Wallis test P-value: 0.001
Pearson correlation coefficient: 0.12 (P<0.001)

© 2018 Downing NS et al. *JAMA Network Open.*

Panel F: Heart failure – black patients

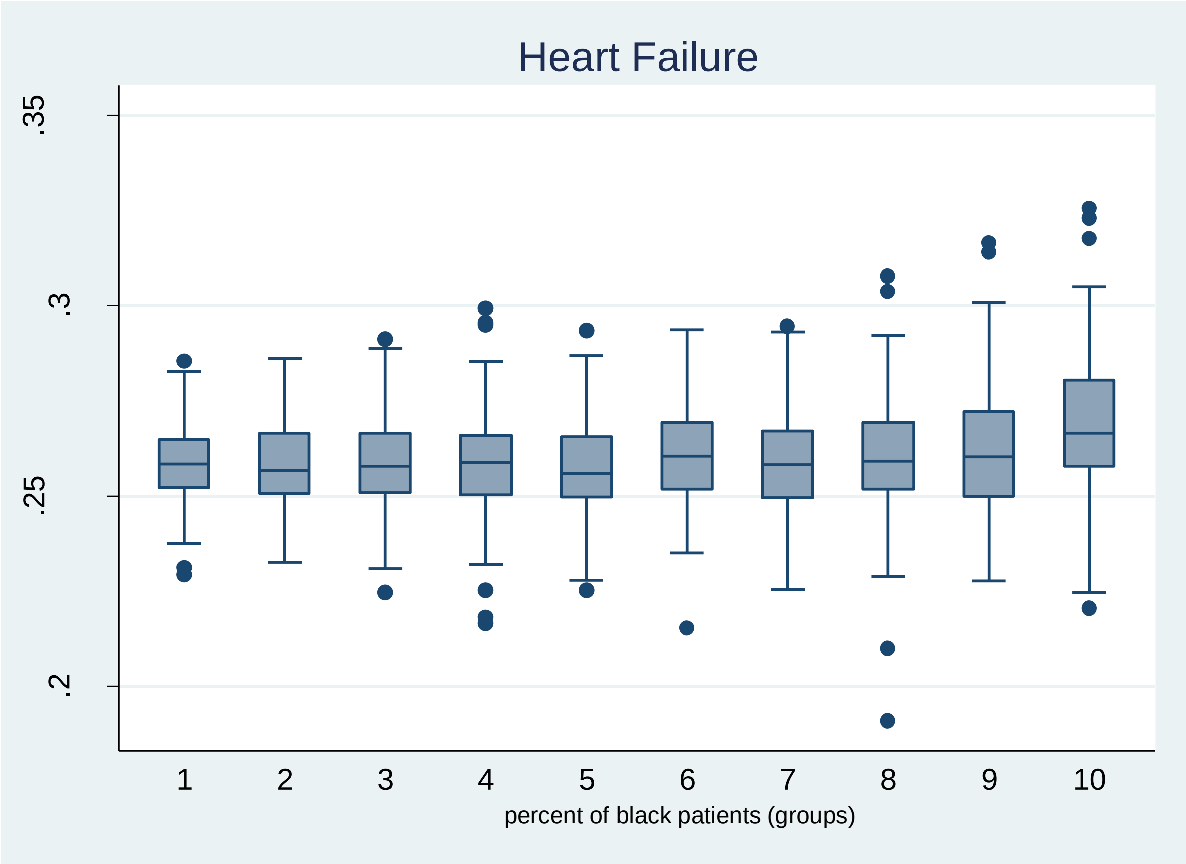

Kruskal-Wallis test P-value: <0.01
Pearson correlation coefficient: 0.22 (P<0.001)

© 2018 Downing NS et al. *JAMA Network Open.*

Panel G: Pneumonia – all patients

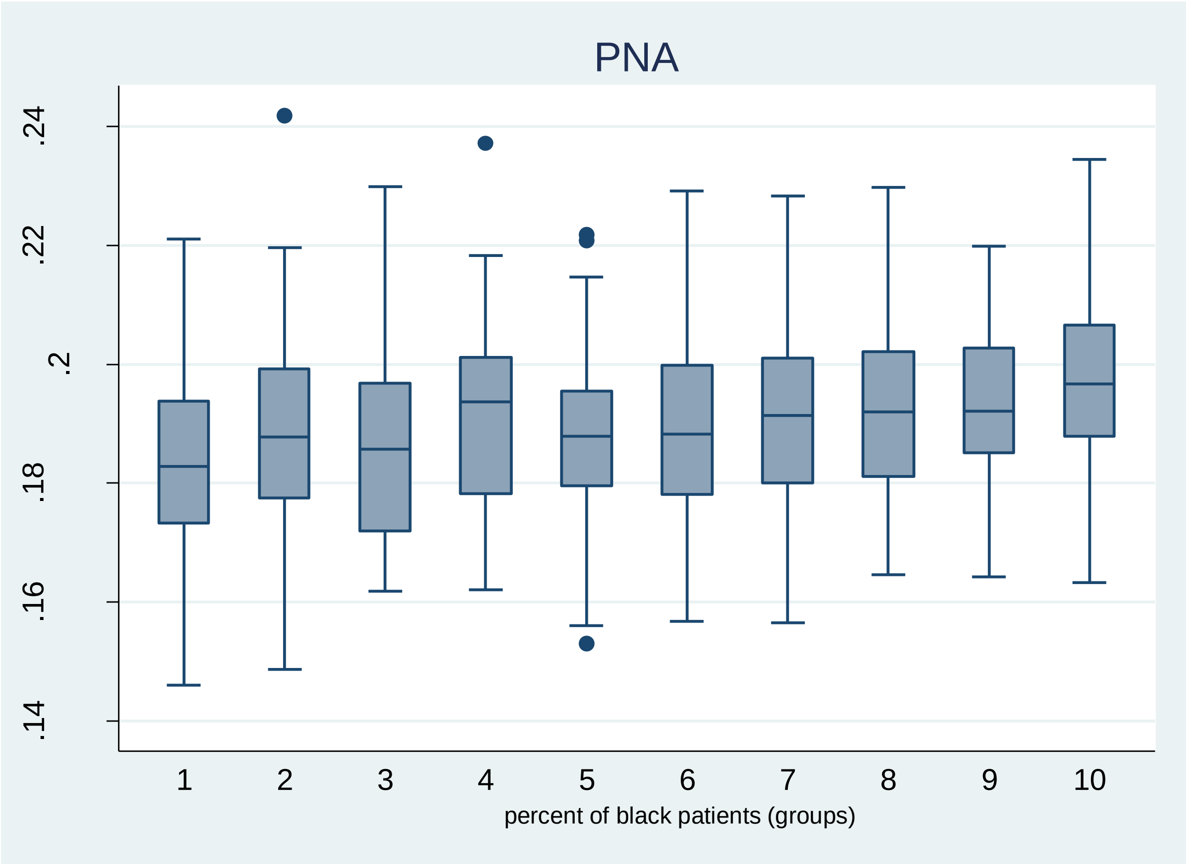

Kruskal-Wallis test P-value: 0.002
Pearson correlation coefficient: 0.17 (P<0.001)

© 2018 Downing NS et al. *JAMA Network Open.*

Panel H: Pneumonia – white patients

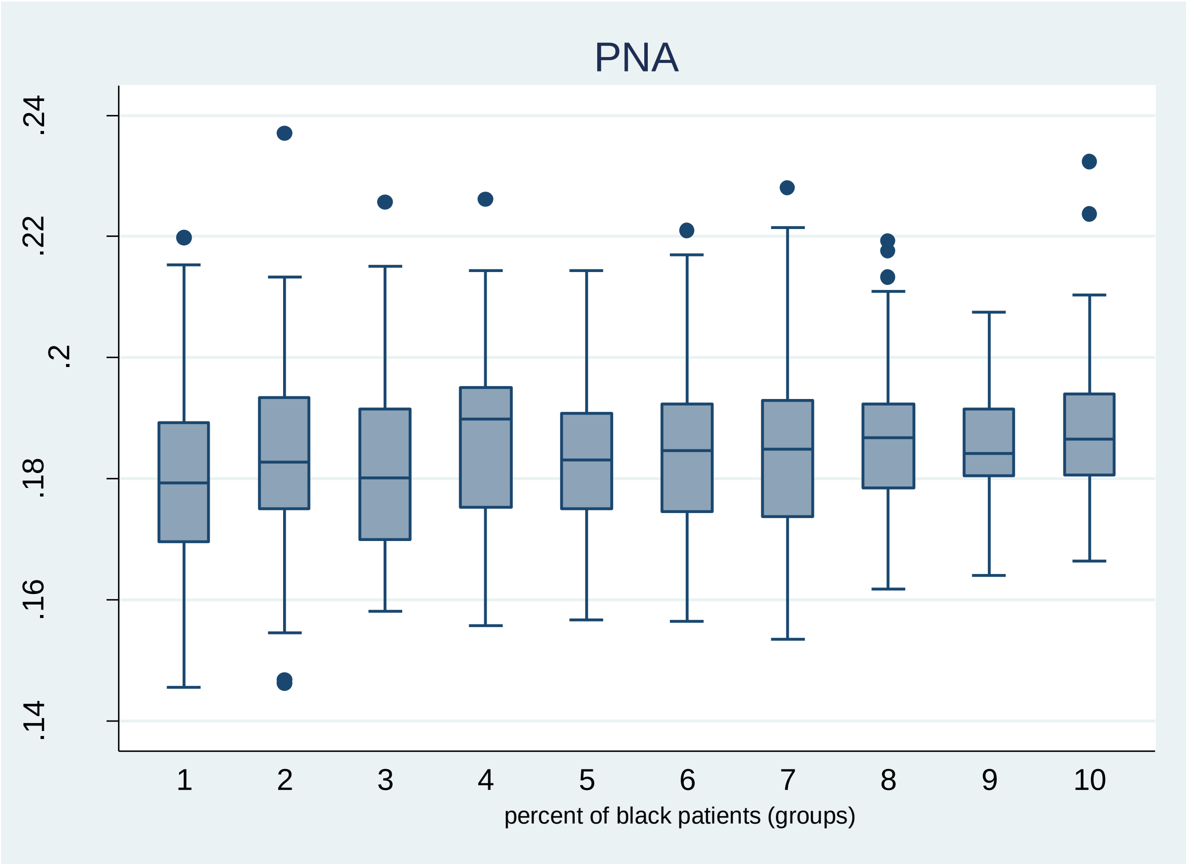

Kruskal-Wallis test P-value: 0.001
Pearson correlation coefficient: 0.12 (P=0.001)

© 2018 Downing NS et al. *JAMA Network Open.*

Panel I: Pneumonia – black patients

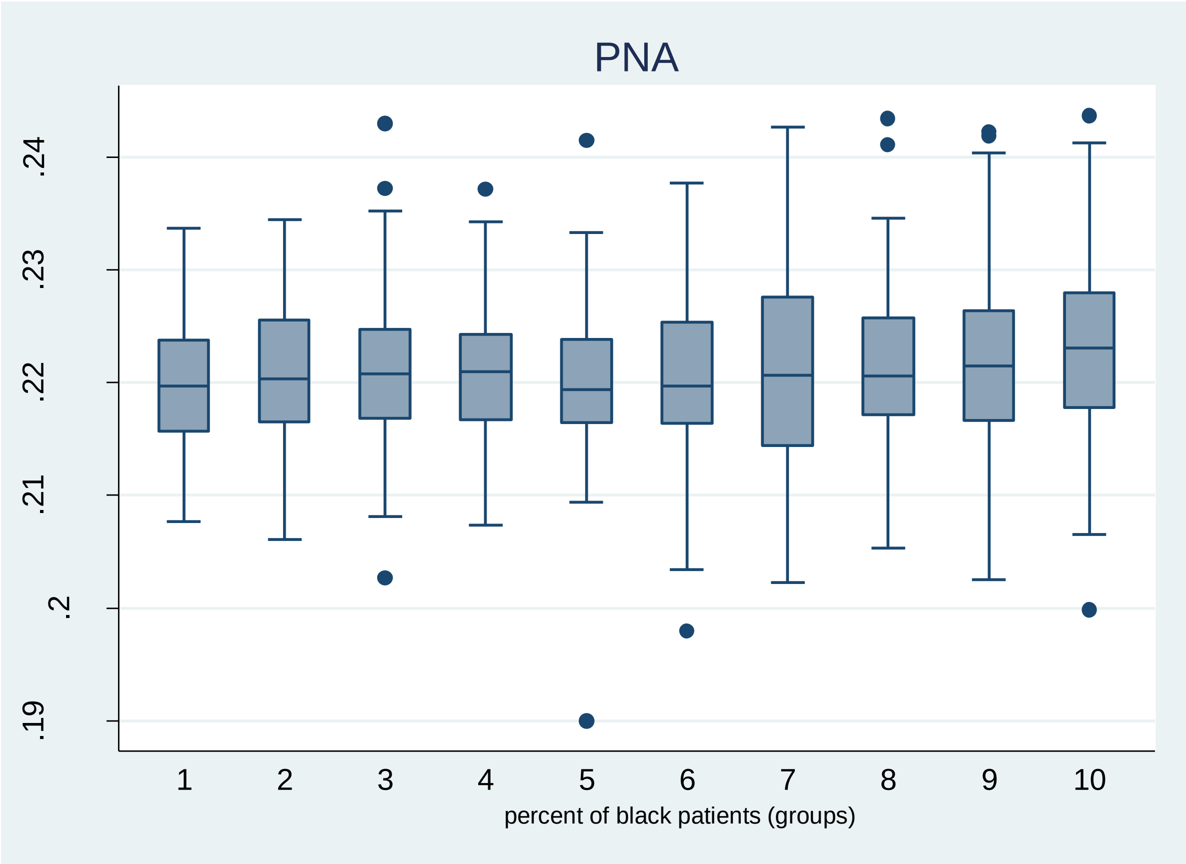

Kruskal-Wallis test P-value: 0.20
Pearson correlation coefficient: 0.10 (P=0.002)

© 2018 Downing NS et al. *JAMA Network Open.*

APPENDIX FIGURE 5. Scatterplots showing between-hospital variation in risk-standardized mortality rates (RSMRs) and risk-standardized readmission rates (RSRRs) according to neighborhood income. Hospital RSMRs and RSRRs for acute myocardial infarction (AMI), heart failure, and pneumonia among all patients and among patients from lower-income neighborhoods are plotted against the proportion of patients from lower-income neighborhoods treated at each hospital for these conditions.

Panel A: Risk-standardized mortality rates

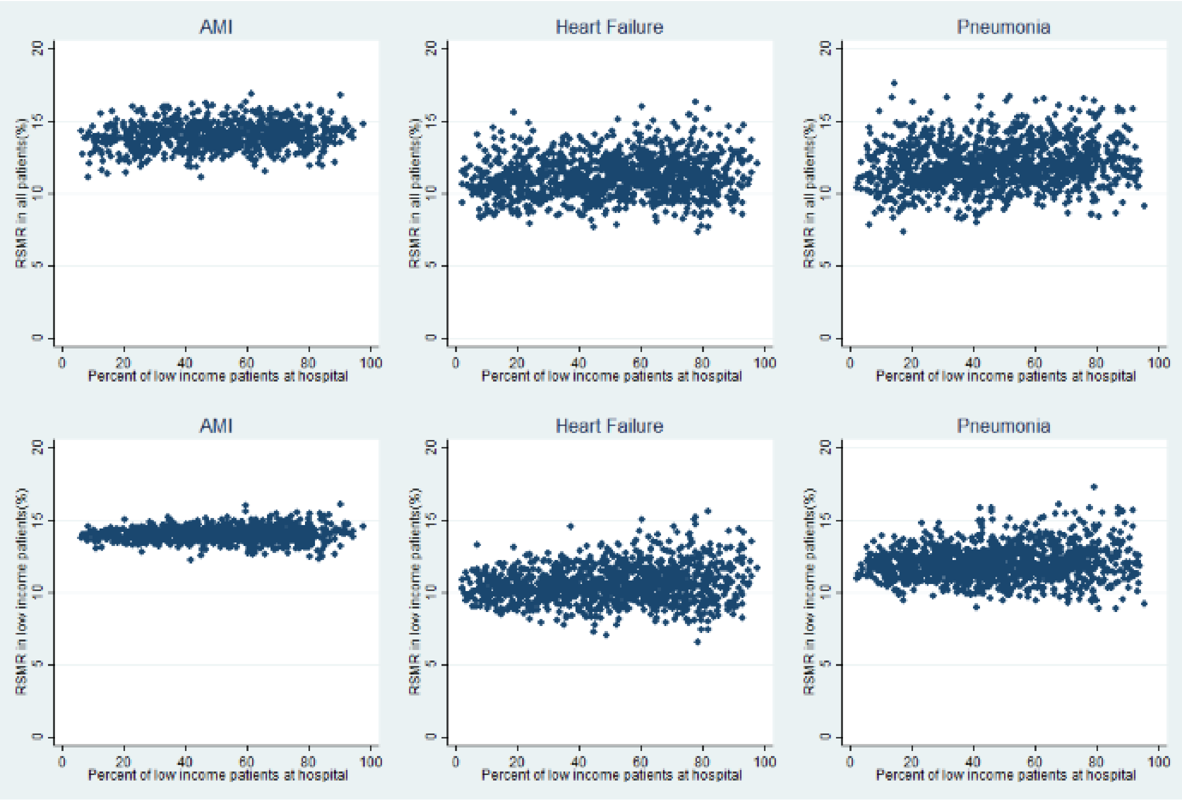

© 2018 Downing NS et al. *JAMA Network Open.*

Panel B: Risk-standardized readmission rates

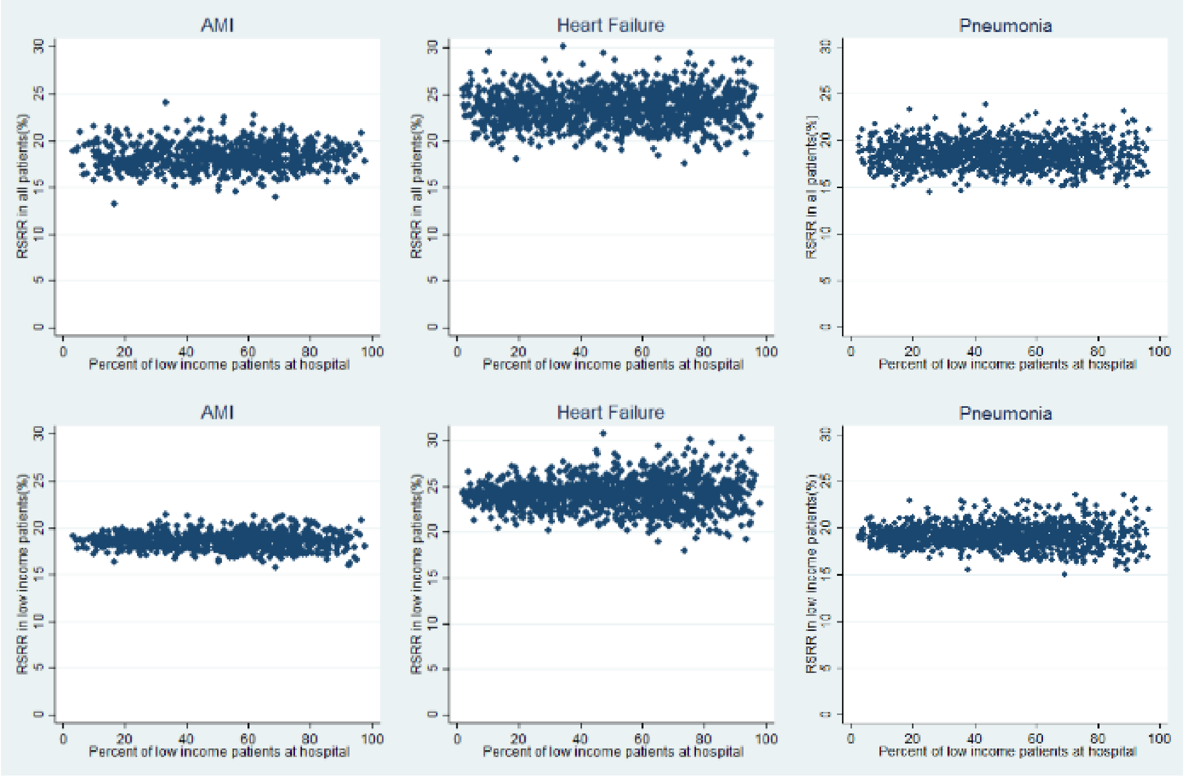

© 2018 Downing NS et al. *JAMA Network Open.*

APPENDIX FIGURE 6. Risk-standardized mortality rates (RSMRs) for all patients, patients from higher-income neighborhoods and patients from lower-income neighborhoods treated for acute myocardial infarction, heart failure and pneumonia by decile of the proportion of patients from lower-income neighborhoods treated at each hospital.

Panel A: Acute myocardial infarction – all patients

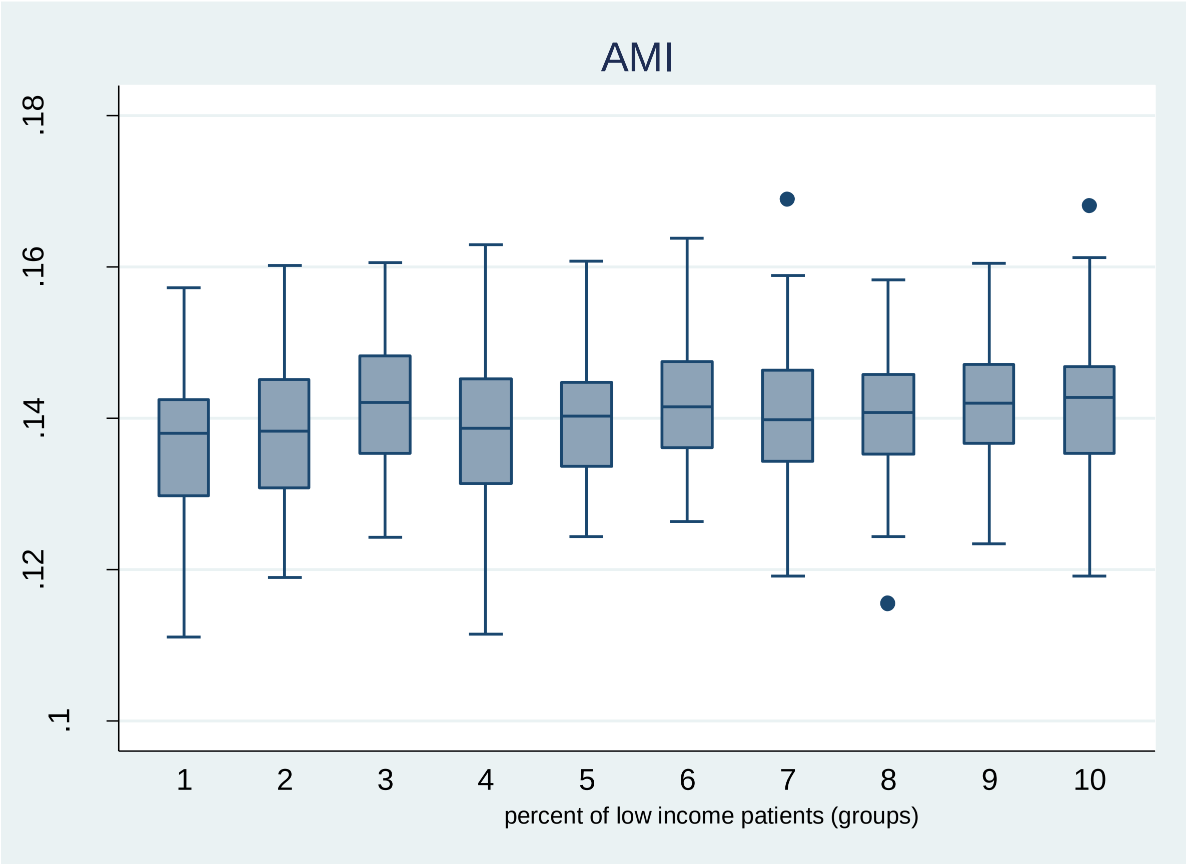

Kruskal-Wallis test P-value: 0.004
Pearson correlation coefficient: 0.12 (P=0.001)

© 2018 Downing NS et al. *JAMA Network Open.*

Panel B: Acute myocardial infarction – patients from higher-income neighborhoods

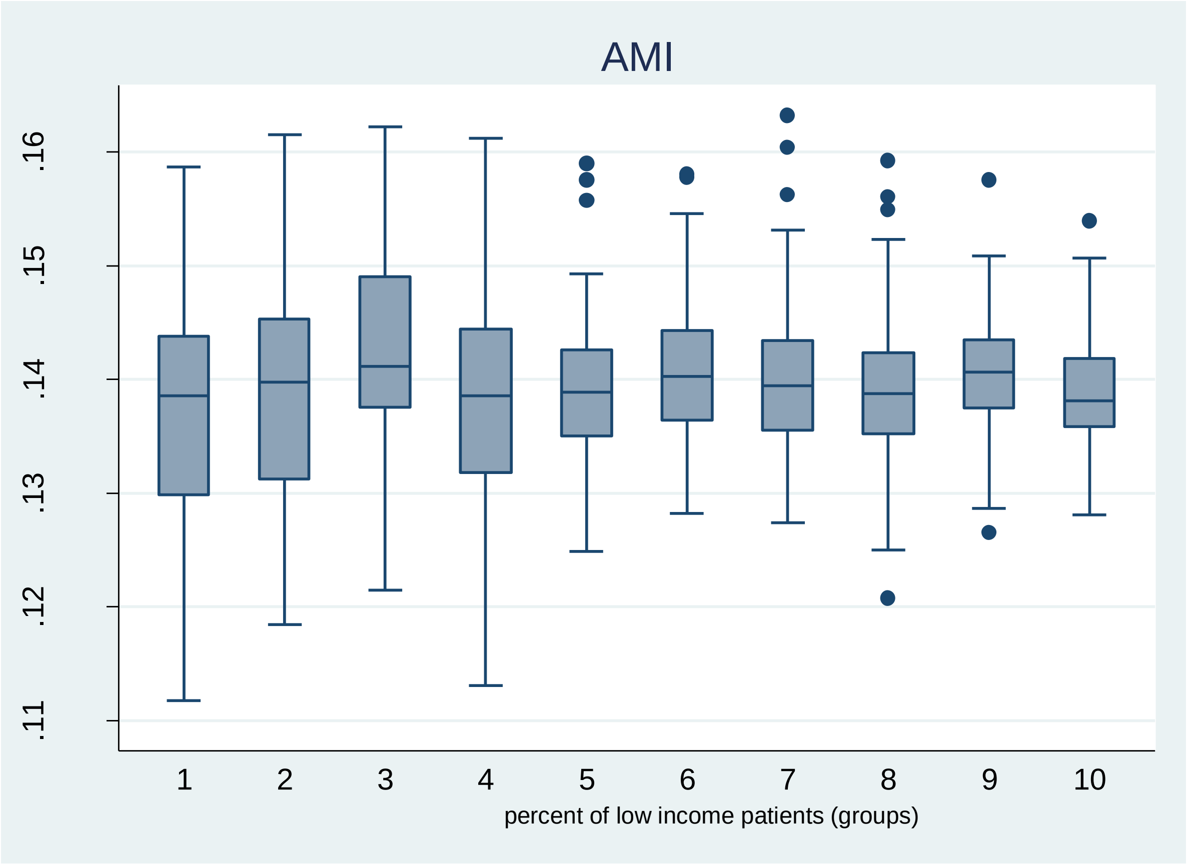

Kruskal-Wallis test P-value: 0.04
Pearson correlation coefficient: 0.03 (P=0.46)

© 2018 Downing NS et al. *JAMA Network Open.*

Panel C: Acute myocardial infarction – patients from lower-income neighborhoods

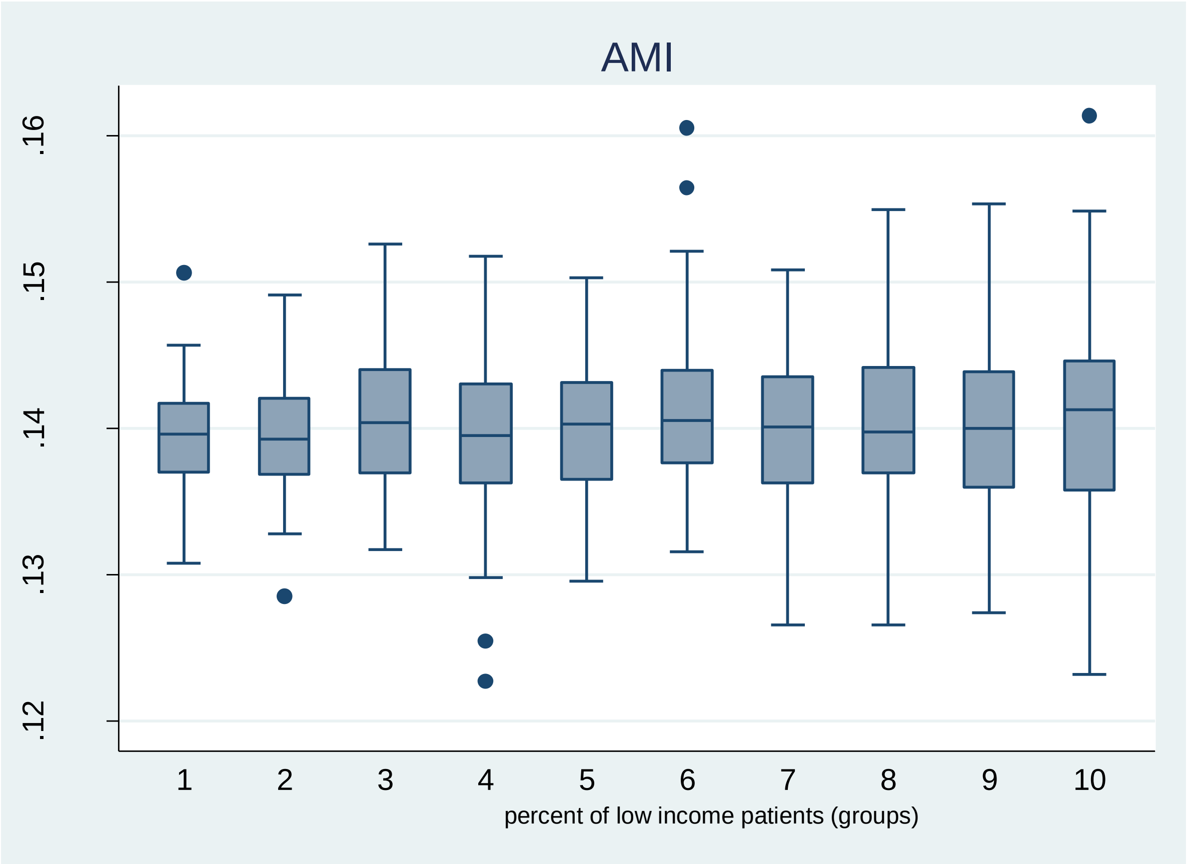

Kruskal-Wallis test P-value: 0.58
Pearson correlation coefficient: 0.07 (P=0.05)

© 2018 Downing NS et al. *JAMA Network Open.*

Panel D: Heart failure – all patients

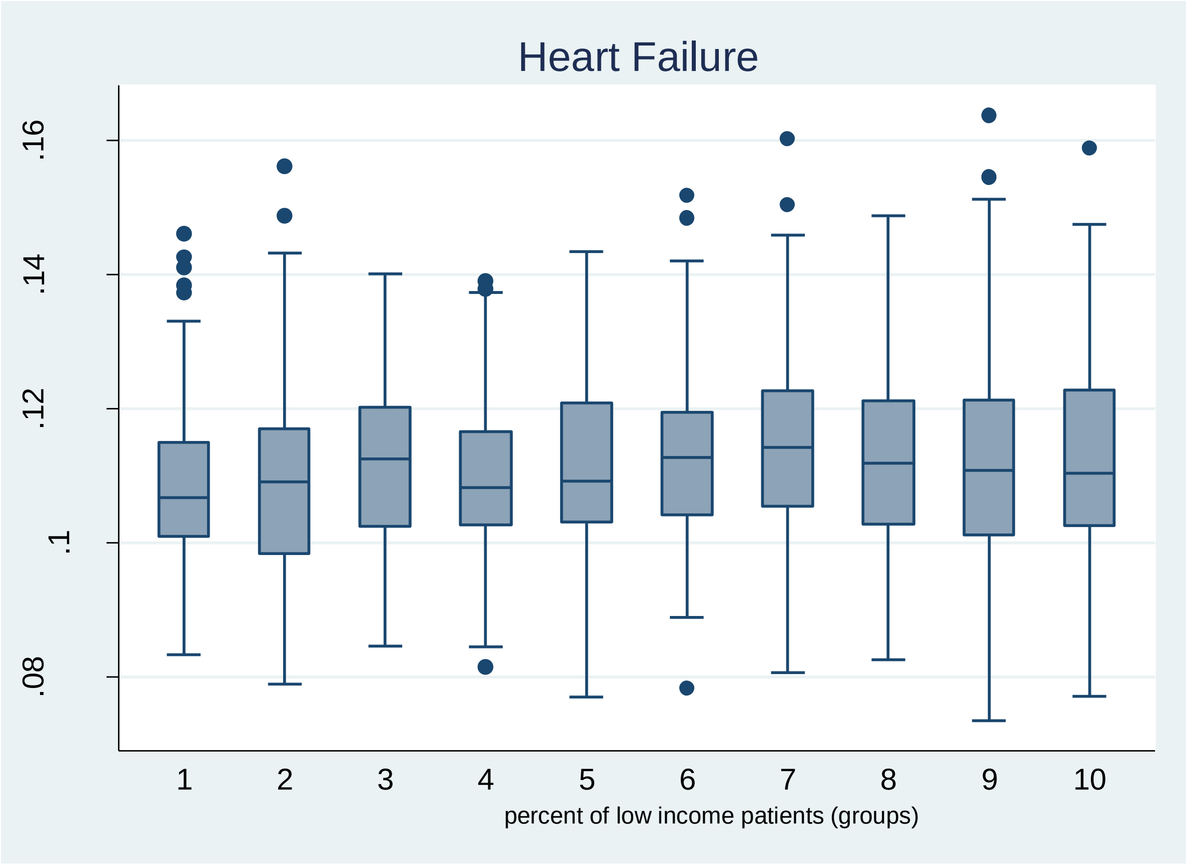

Kruskal-Wallis test P-value: 0.007
Pearson correlation coefficient: 0.12 (P<0.001)

© 2018 Downing NS et al. *JAMA Network Open.*

Panel E: Heart failure – patients from higher-income neighborhoods

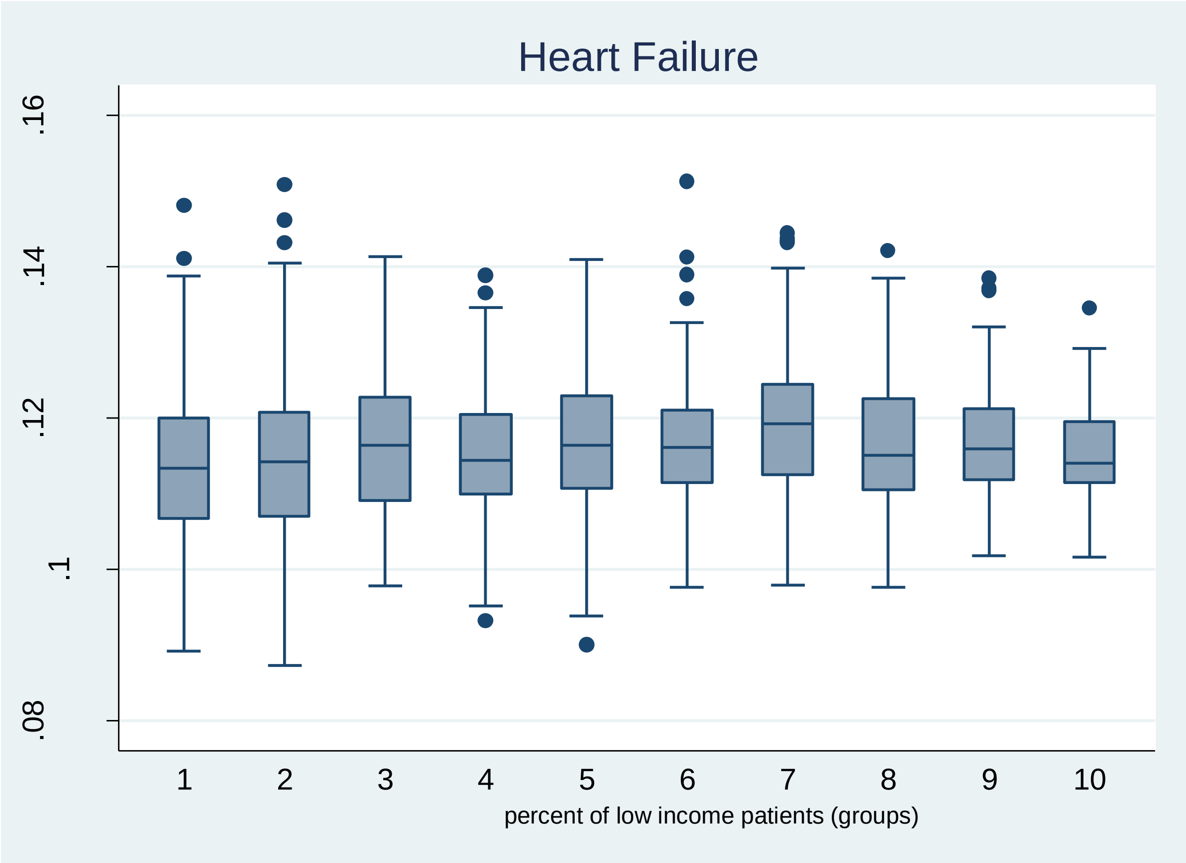

Kruskal-Wallis test P-value: 0.002
Pearson correlation coefficient: 0.07 (P=0.01)

© 2018 Downing NS et al. *JAMA Network Open.*

Panel F: Heart failure – patients from lower-income neighborhoods

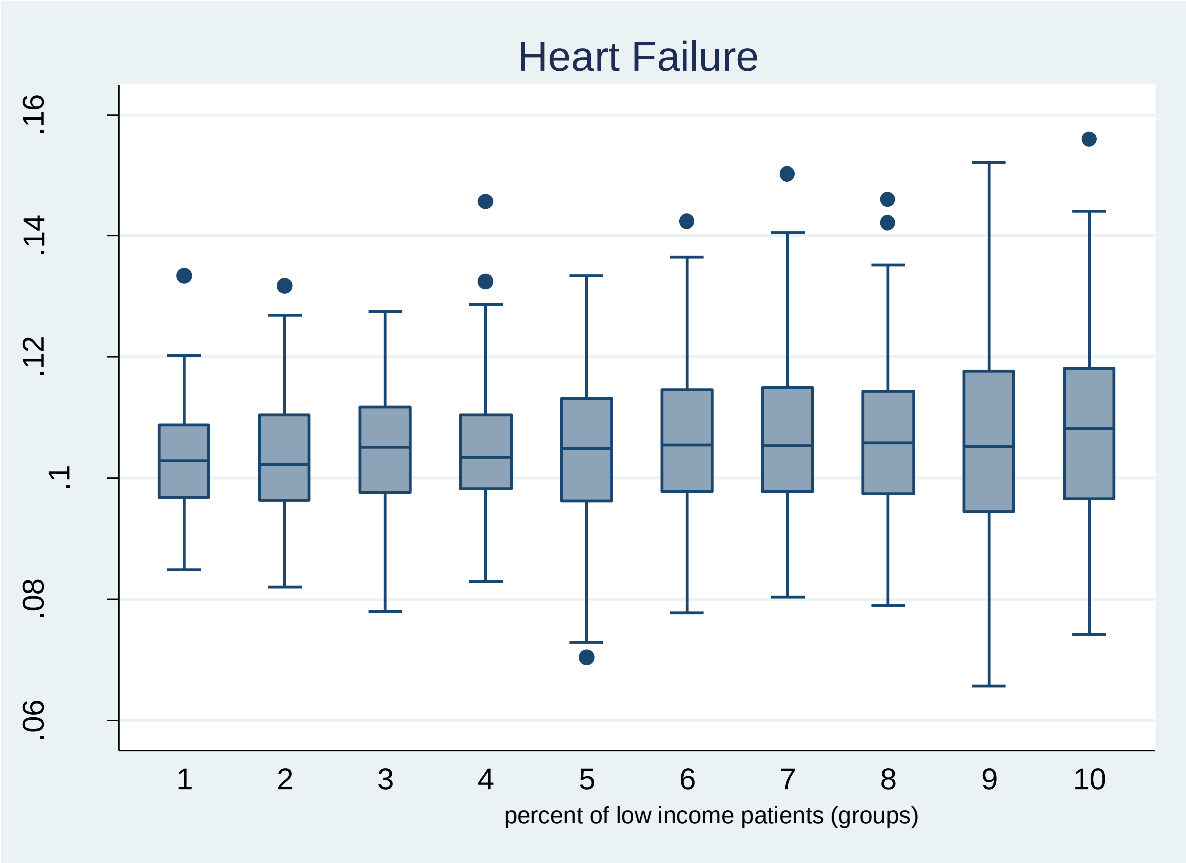

Kruskal-Wallis test P-value: 0.05
Pearson correlation coefficient: 0.13 (P<0.001)

© 2018 Downing NS et al. *JAMA Network Open.*

Panel G: Pneumonia – all patients

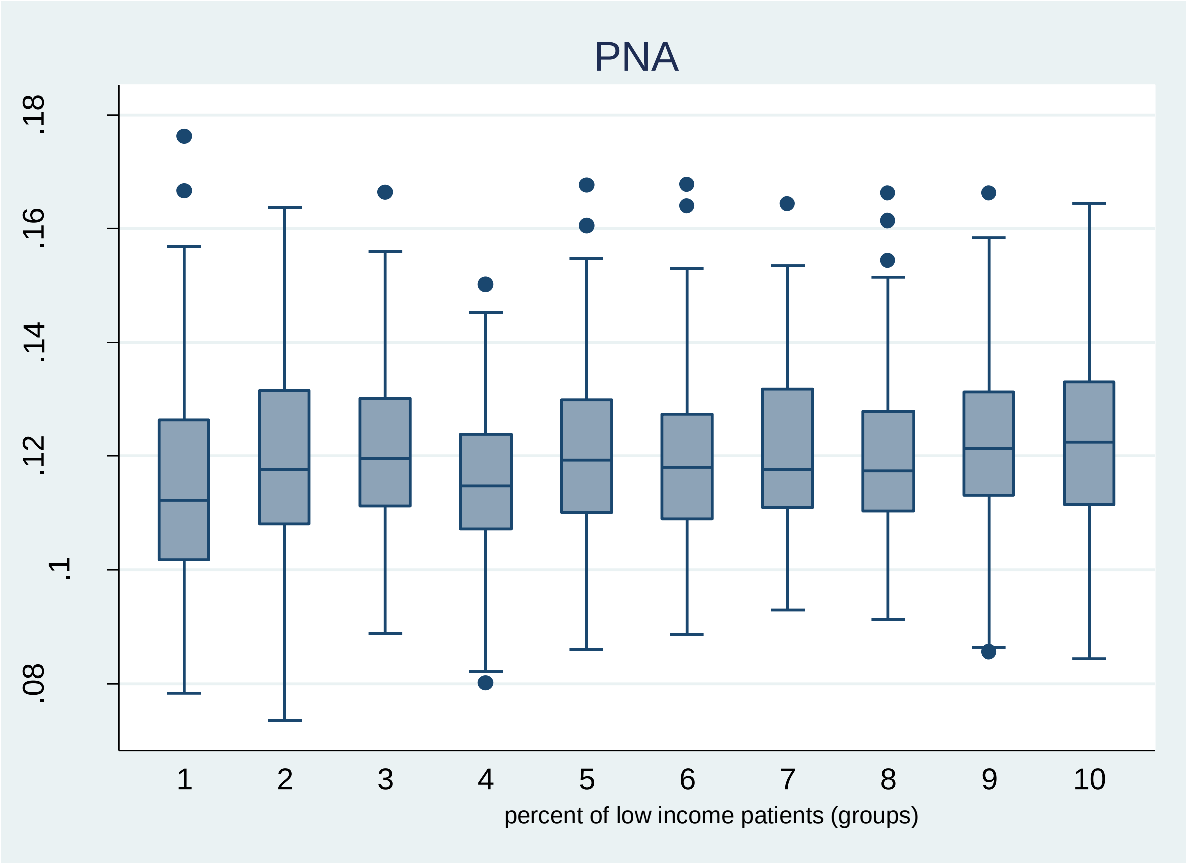

Kruskal-Wallis test P-value: <0.001
Pearson correlation coefficient: 0.13 (P<0.001)

© 2018 Downing NS et al. *JAMA Network Open.*

Panel H: Pneumonia – patients from higher-income neighborhoods

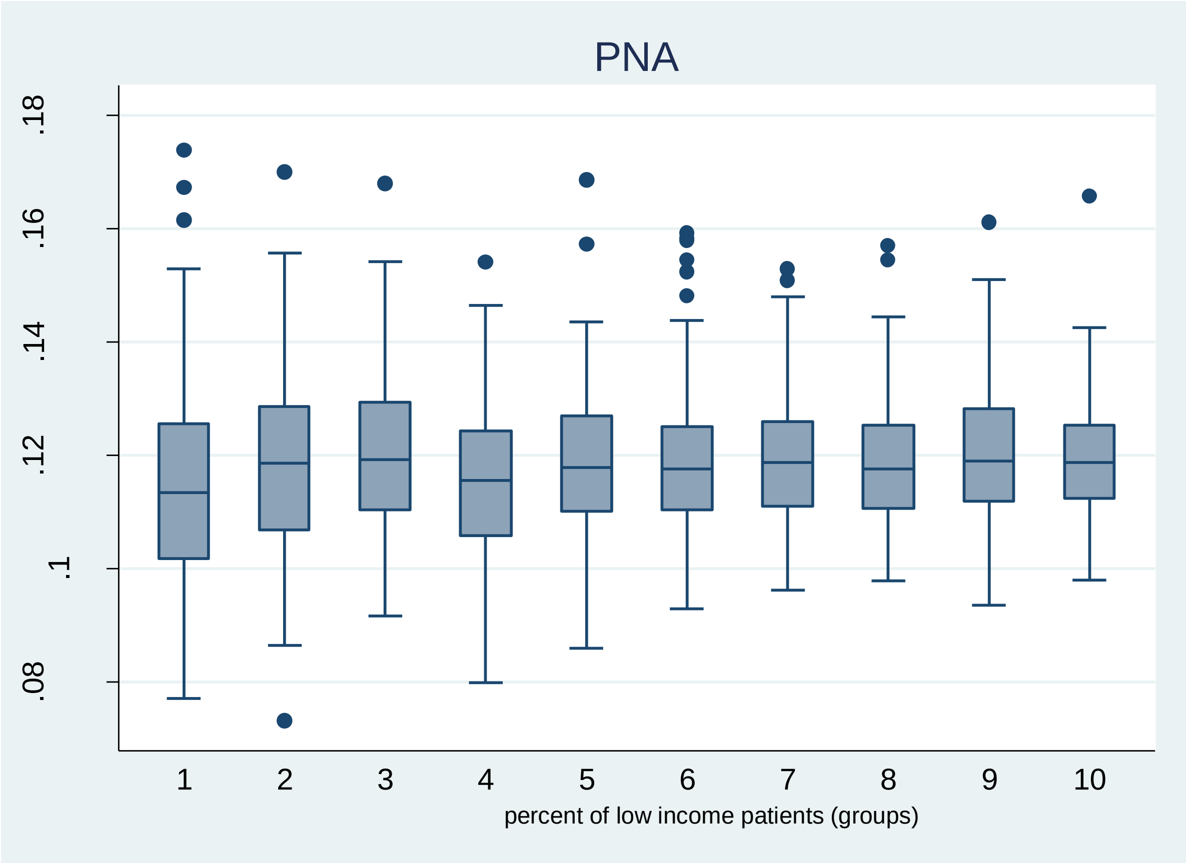

Kruskal-Wallis test P-value: 0.02
Pearson correlation coefficient: 0.08 (P=0.008)

© 2018 Downing NS et al. *JAMA Network Open.*

Panel I: Pneumonia – patients from lower-income neighborhoods

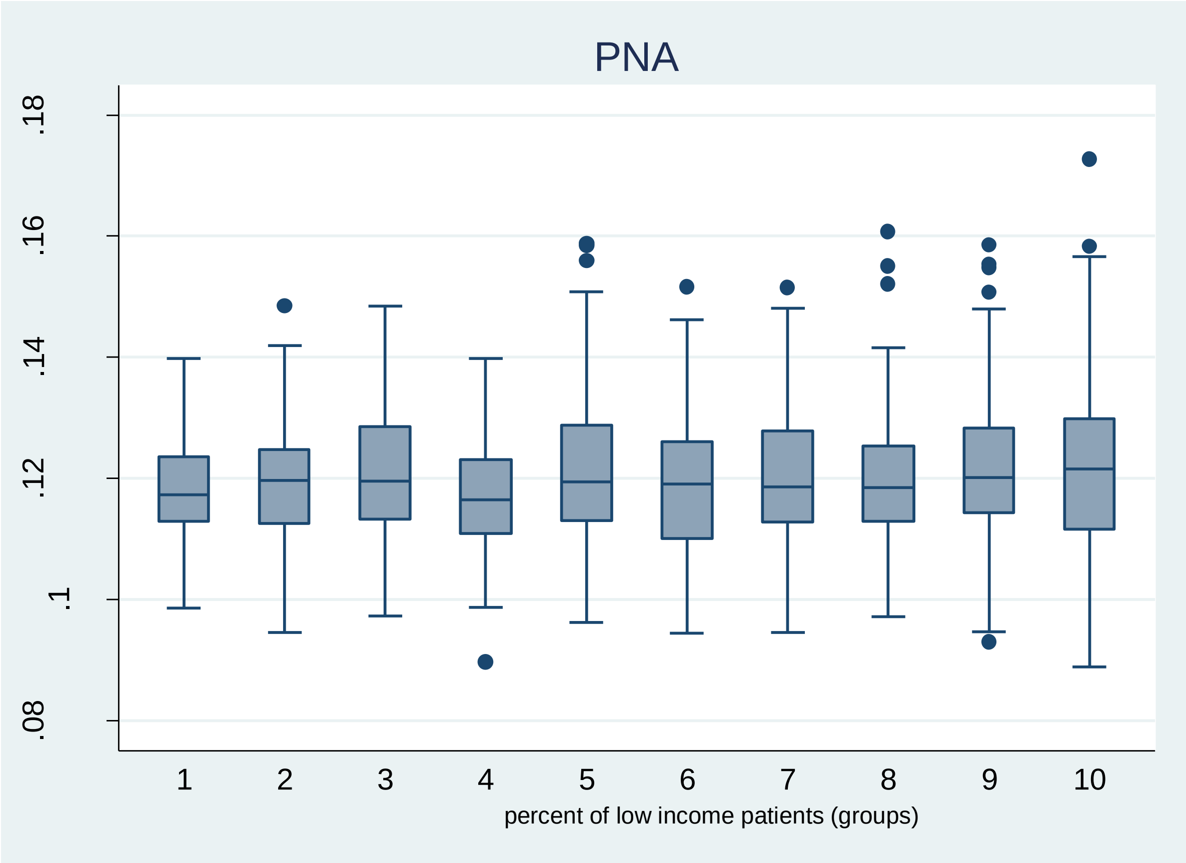

Kruskal-Wallis test P-value: 0.03
Pearson correlation coefficient: 0.10 (P=0.002)

© 2018 Downing NS et al. *JAMA Network Open.*

APPENDIX FIGURE 7. Risk-standardized readmission rates (RSRRs) for all patients, patients from higher-income neighborhoods, and patients from lower-income neighborhoods treated for acute myocardial infarction, heart failure and pneumonia by decile of the proportion of patients from lower-income neighborhoods treated at each hospital.

Panel A: Acute myocardial infarction – all patients

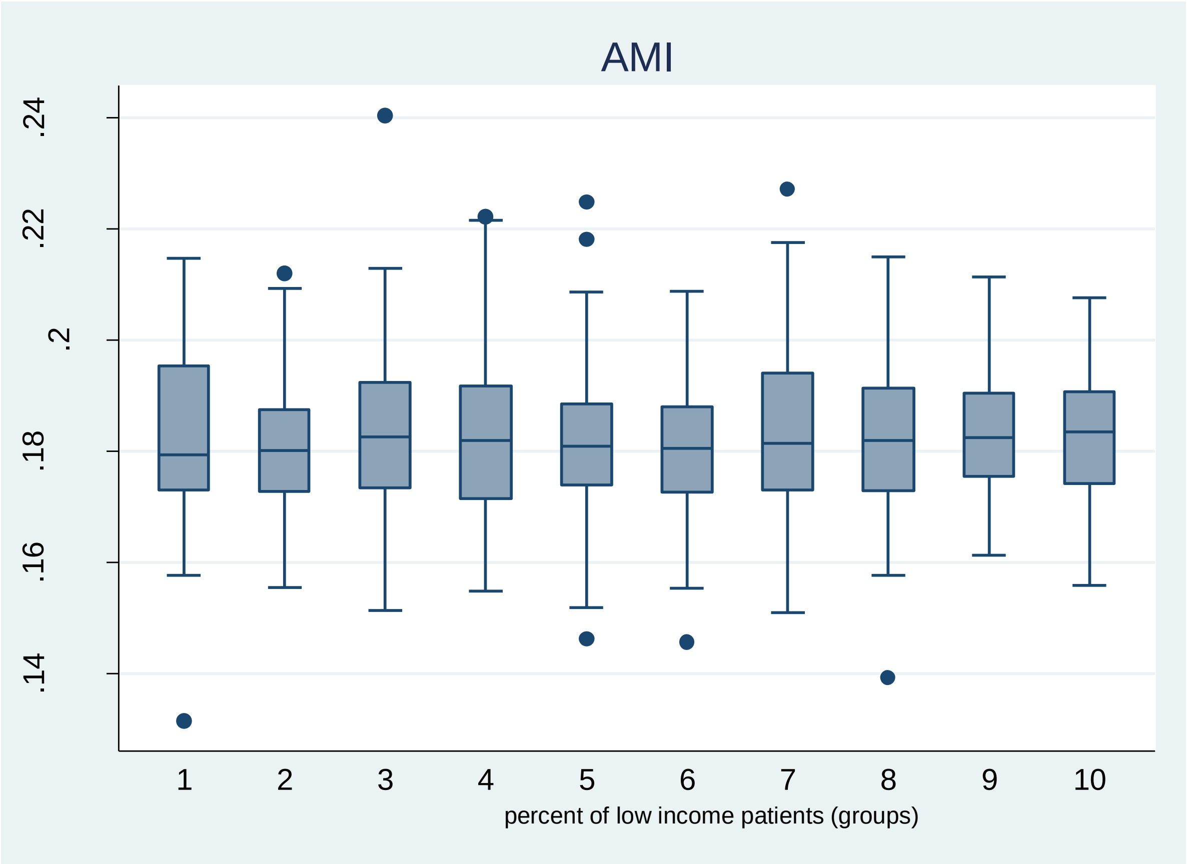

Kruskal-Wallis test P-value: 0.71
Pearson correlation coefficient: -0.02 (P=0.61)

© 2018 Downing NS et al. *JAMA Network Open.*

Panel B: Acute myocardial infarction – patients from higher-income neighborhoods

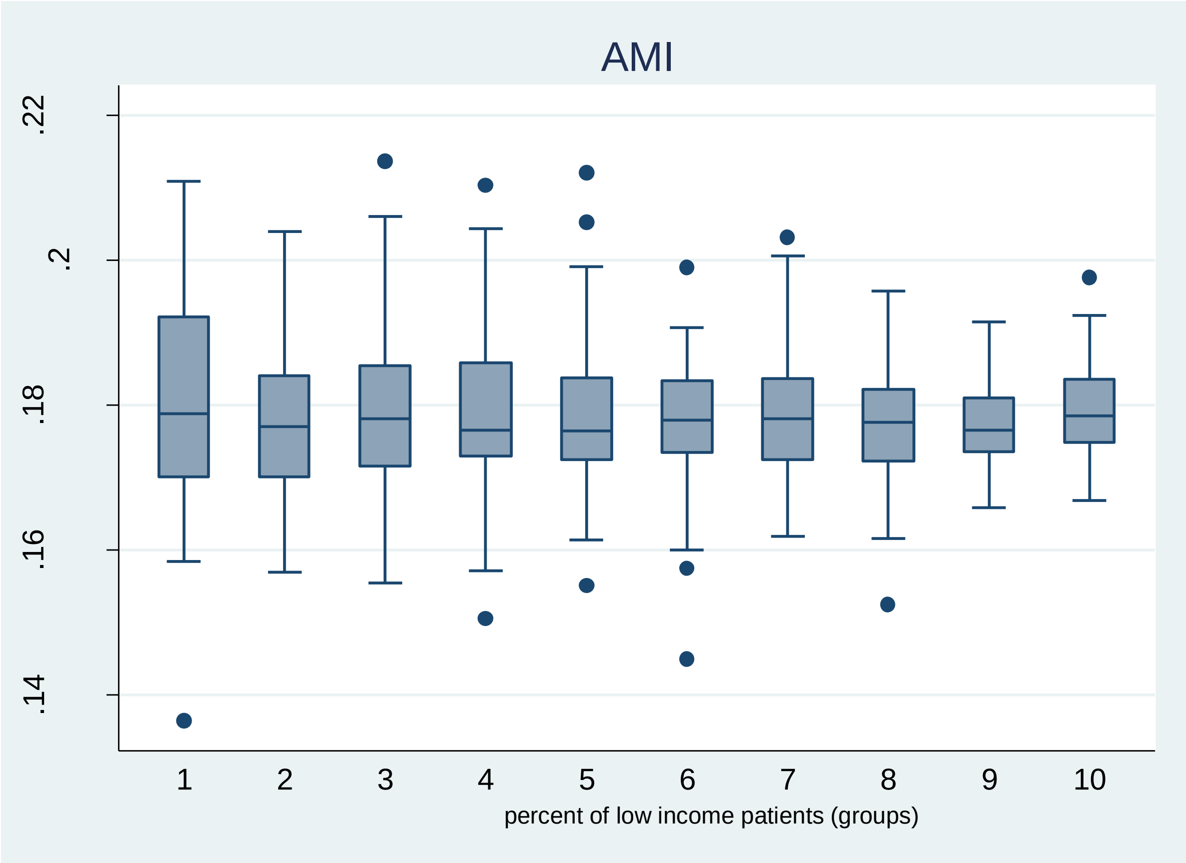

Kruskal-Wallis test P-value: 0.88
Pearson correlation coefficient: -0.03 (P=0.36)

© 2018 Downing NS et al. *JAMA Network Open.*

Panel C: Acute myocardial infarction – patients from lower-income neighborhoods

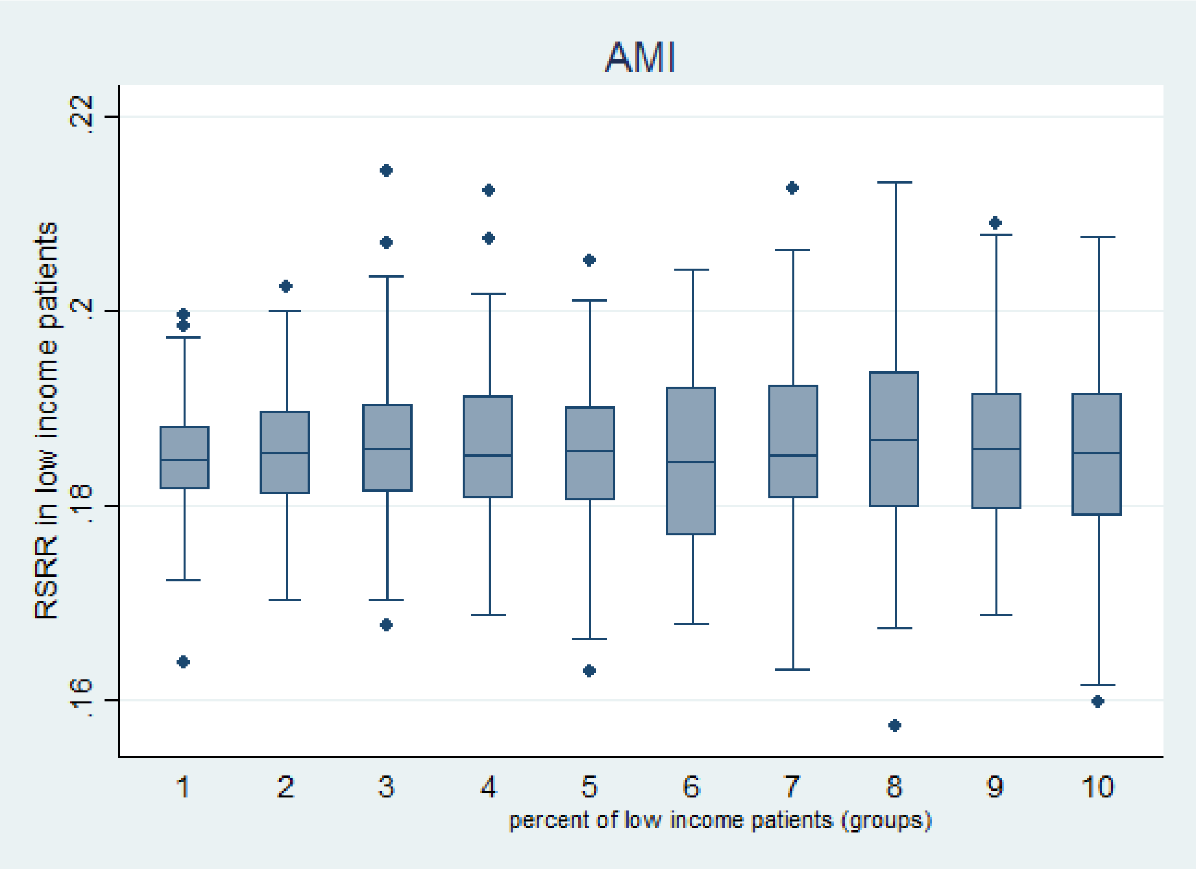

Kruskal-Wallis test P-value: 0.91

Pearson correlation coefficient: 0.01 (P=0.75)

© 2018 Downing NS et al. *JAMA Network Open.*

Panel D: Heart failure – all patients

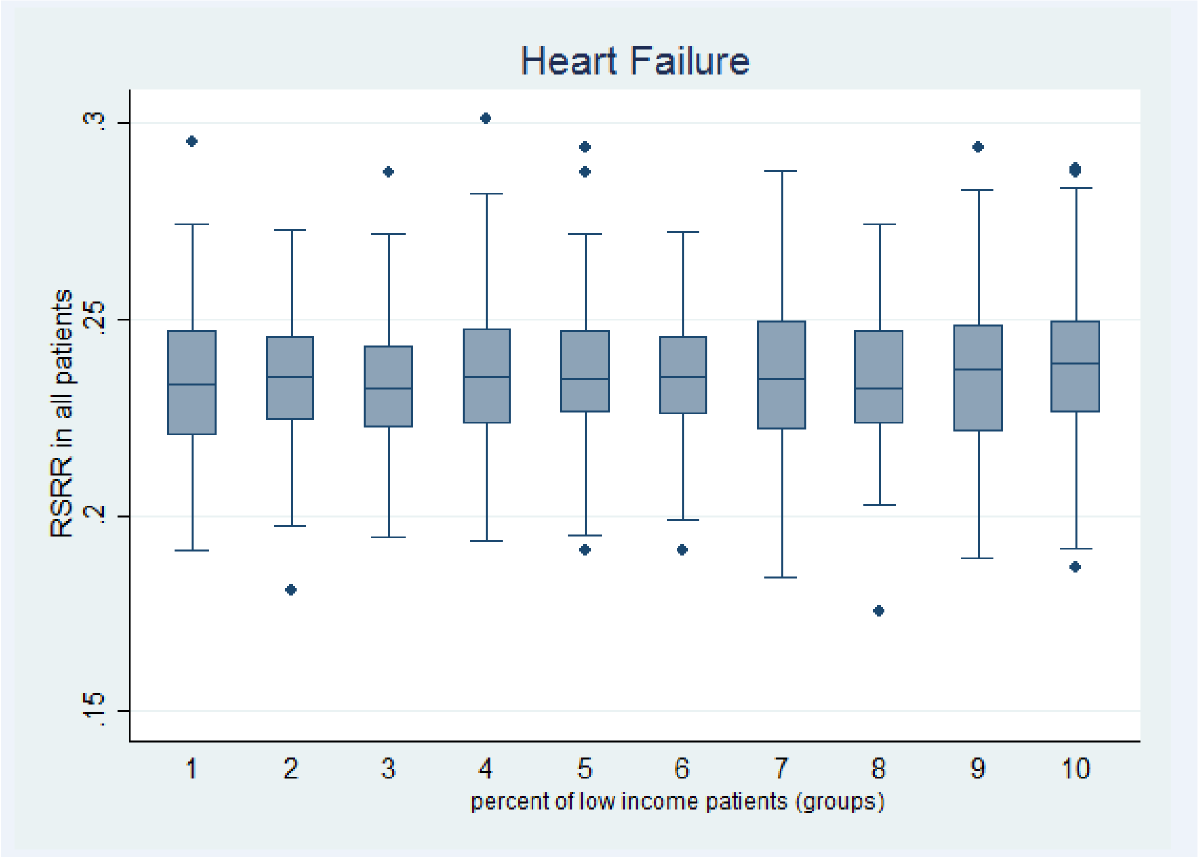

Kruskal-Wallis test P-value: 0.79

Pearson correlation coefficient: 0.03 (P=0.24)

© 2018 Downing NS et al. *JAMA Network Open.*

Panel E: Heart failure – patients from higher-income neighborhoods

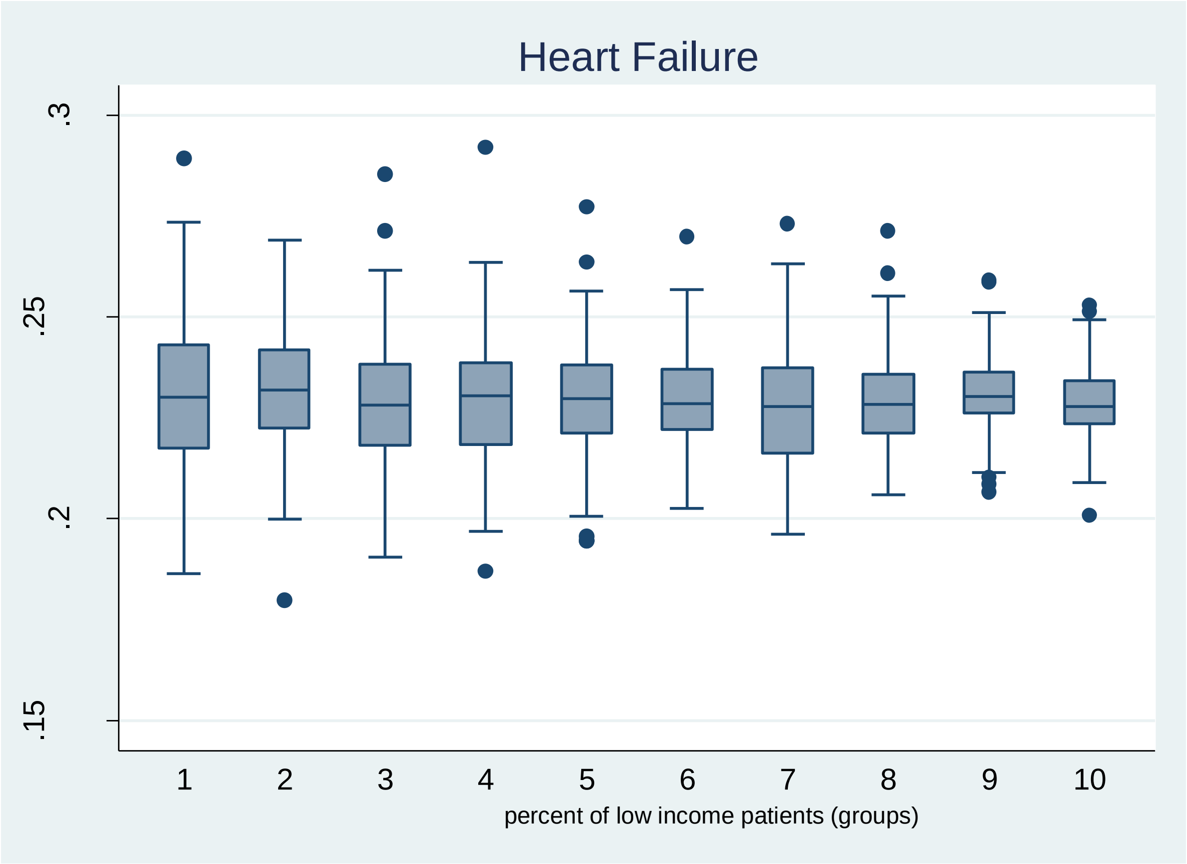

Kruskal-Wallis test P-value: 0.37
Pearson correlation coefficient: -0.03 (P=0.28)

© 2018 Downing NS et al. *JAMA Network Open.*

Panel F: Heart failure – patients from lower-income neighborhoods

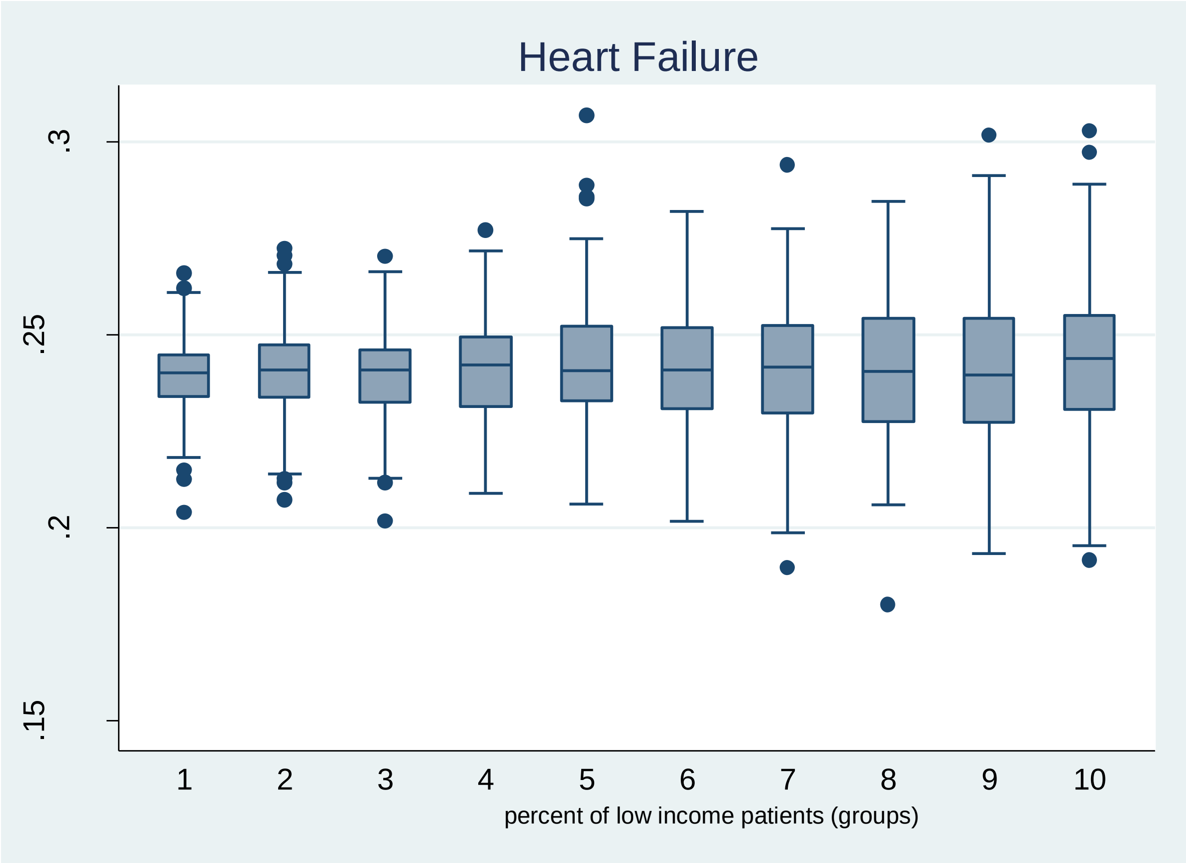

Kruskal-Wallis test P-value: 0.79
Pearson correlation coefficient: 0.04 (P=0.19)

© 2018 Downing NS et al. *JAMA Network Open.*

Panel G: Pneumonia – all patients

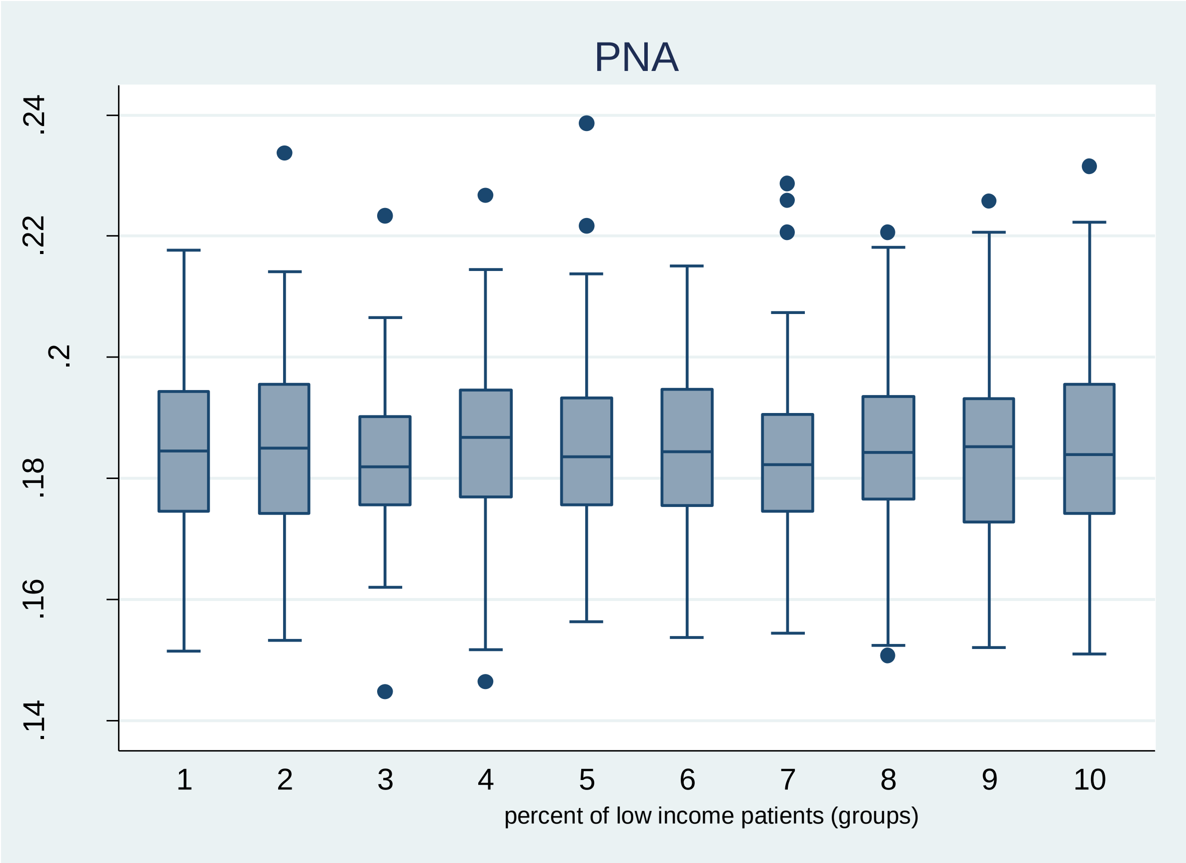

Kruskal-Wallis test P-value: 0.76
Pearson correlation coefficient: -0.008 (P=0.77)

© 2018 Downing NS et al. *JAMA Network Open.*

Panel H: Pneumonia – patients from higher-income neighborhoods

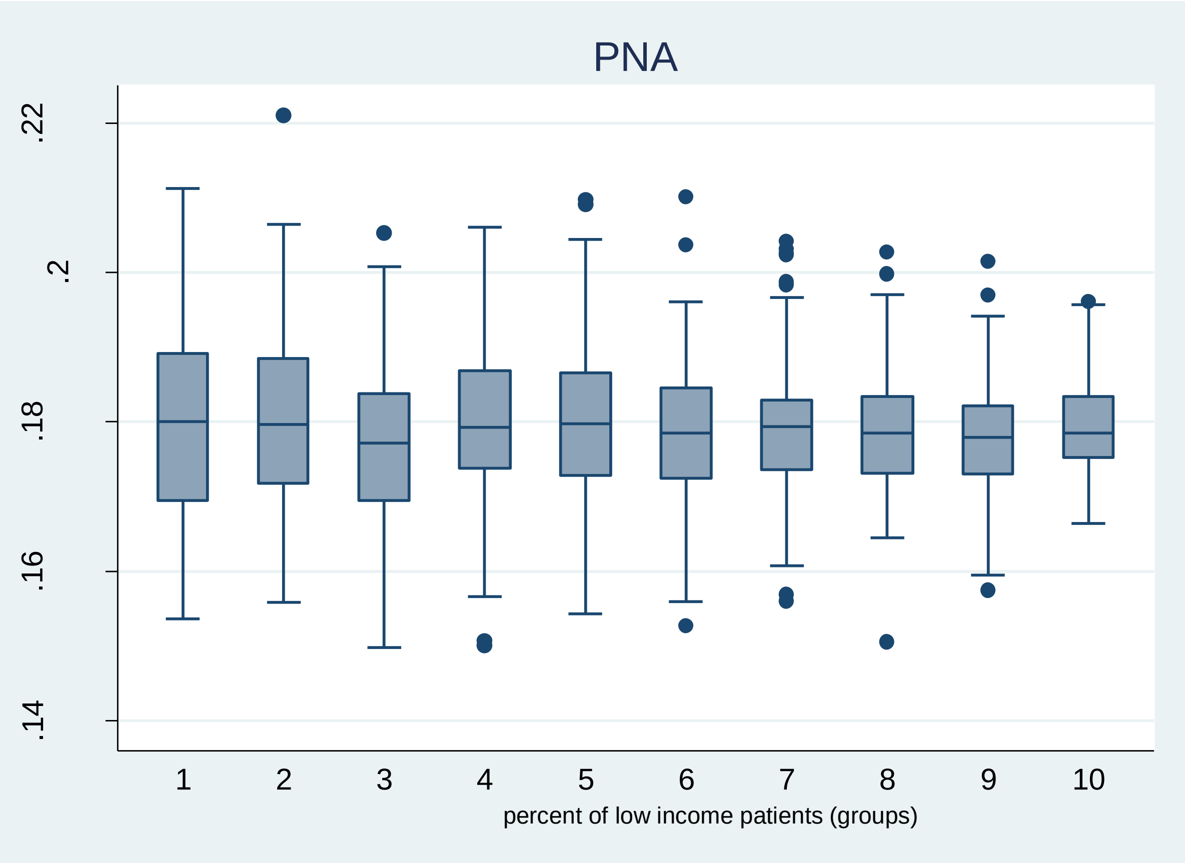

Kruskal-Wallis test P-value: 0.23
Pearson correlation coefficient: -0.004 (P=0.19)

© 2018 Downing NS et al. *JAMA Network Open.*

Panel I: Pneumonia – patients from lower-income neighborhoods

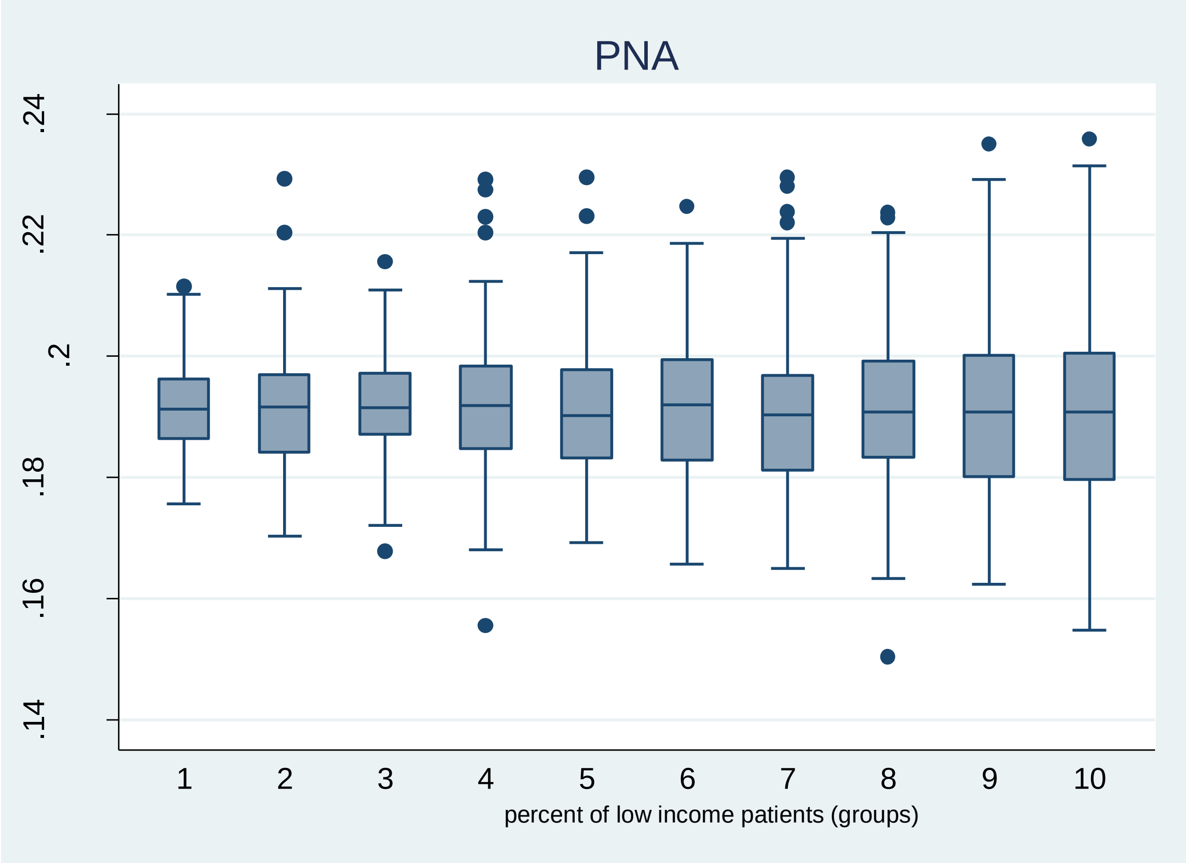

Kruskal-Wallis test P-value: 0.70
Pearson correlation coefficient: -0.03 (P=0.25)

© 2018 Downing NS et al. *JAMA Network Open.*

APPENDIX FIGURE 8: Between-hospital differences in risk-standardized mortality and readmission rates according to race in a sensitivity analysis in which any hospital with at least 10 eligible white patients and 10 eligible black patients was included.

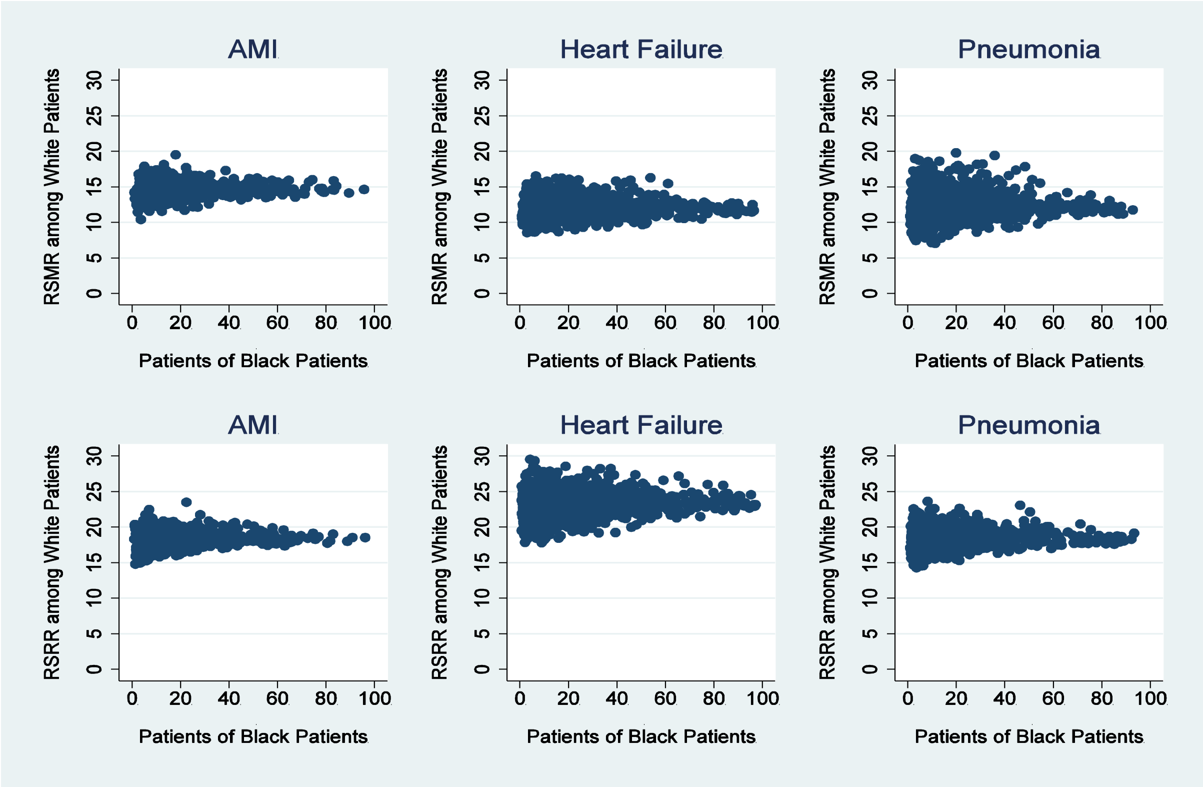

© 2018 Downing NS et al. *JAMA Network Open.*

APPENDIX FIGURE 9: Between-hospital differences in risk-standardized mortality and readmission rates according to neighborhood income in a sensitivity analysis in which any hospital with at least 10 eligible patients from higher-income neighborhoods and 10 eligible patients from lower-income neighborhoods was included.

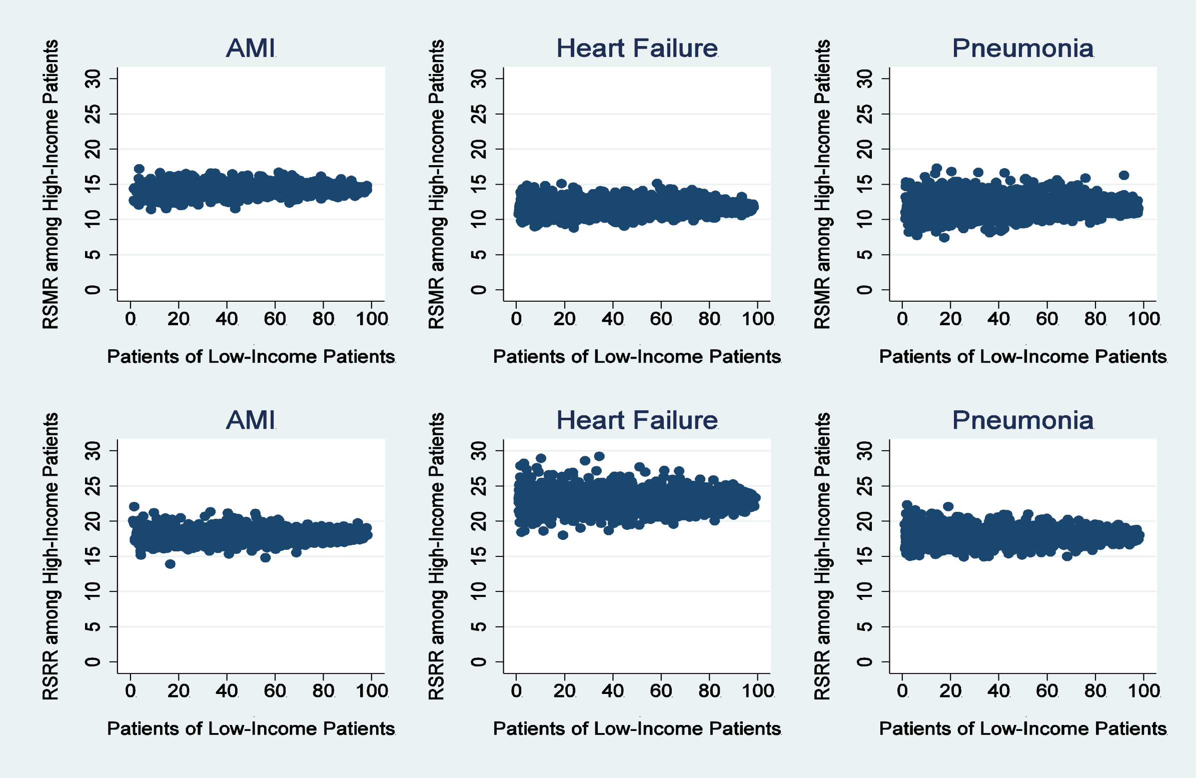

© 2018 Downing NS et al. *JAMA Network Open.*
